# Supplementary figures and images for: Cell-type-specific regulation of neuronal intrinsic excitability by macroautophagy
Source: eLife. 2020 Jan 8;9:e50843. doi: 10.7554/eLife.50843 (PMC6984822; doi:10.7554/eLife.50843)

# Full Length Blots corresponding to Figure 5D

**A** Kir2.1

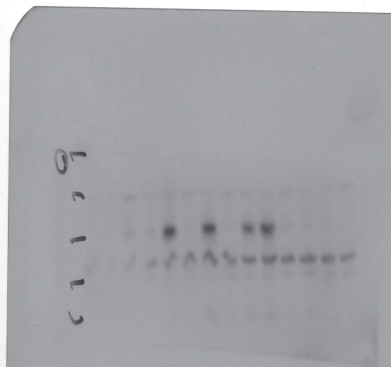

**B** Kir2.3

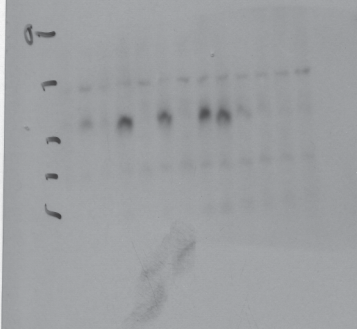

**C** p62

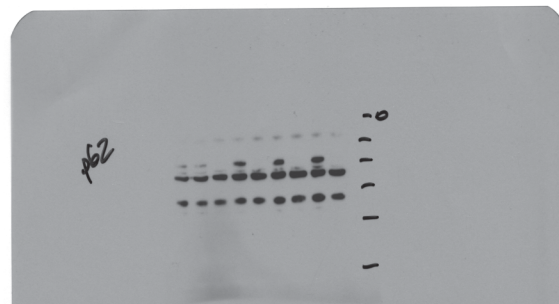

**D** DARPP32

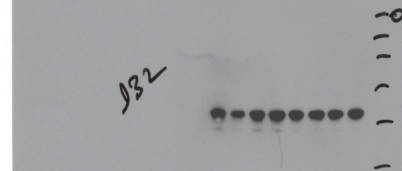

**E** Actin

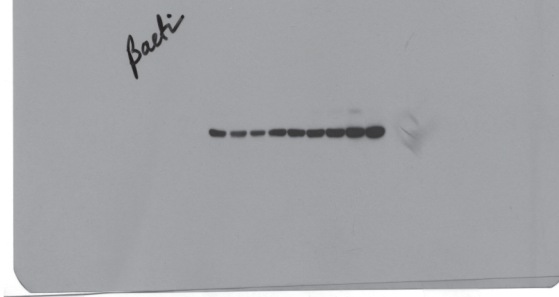

Supplement: Figure 5—source data 1. [file elife-50843-fig5-data1.pdf]

# Full Length Blots corresponding to Figure 5--Fig. Supp 1A

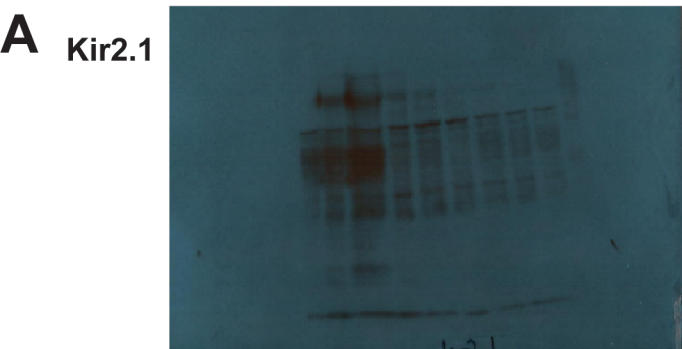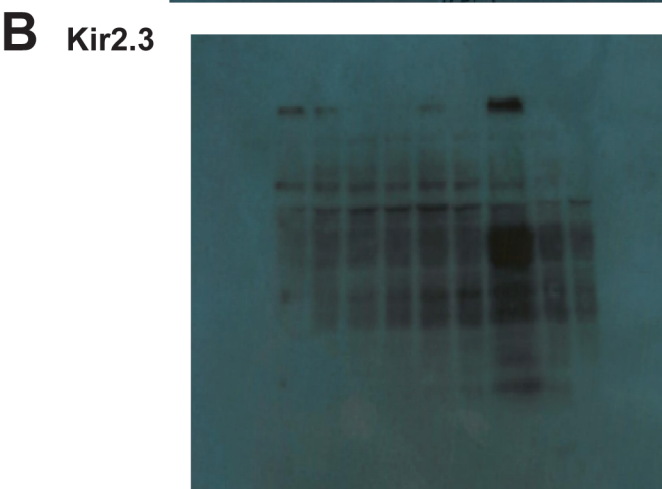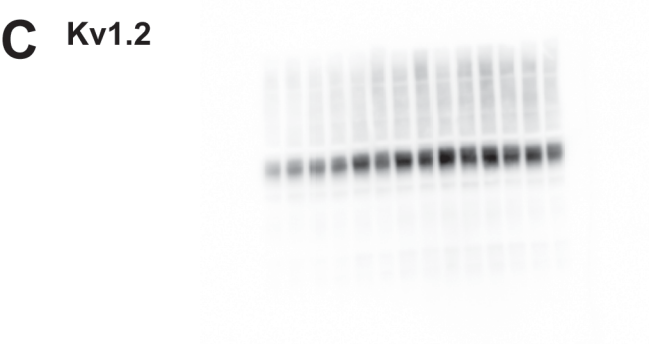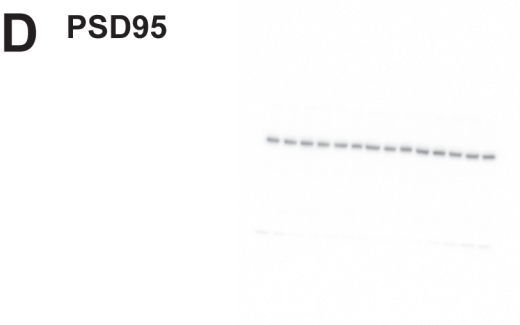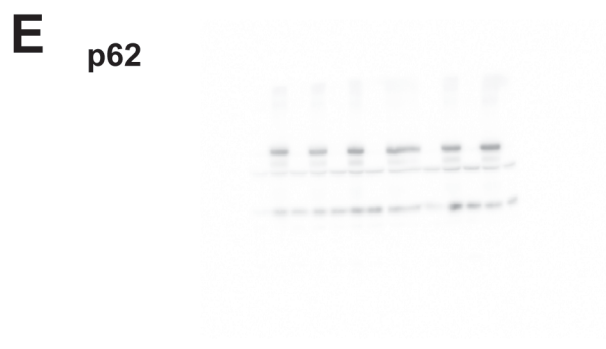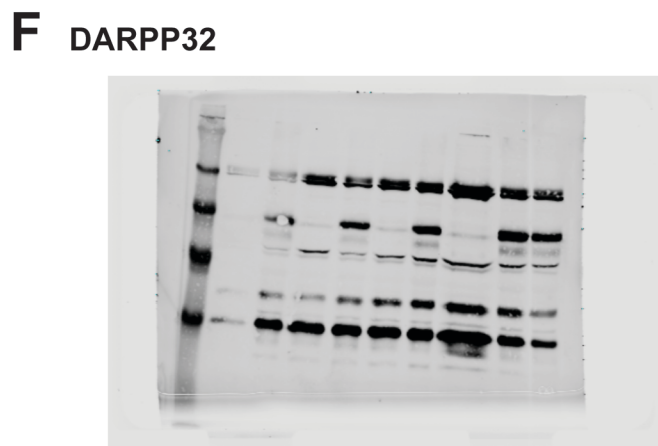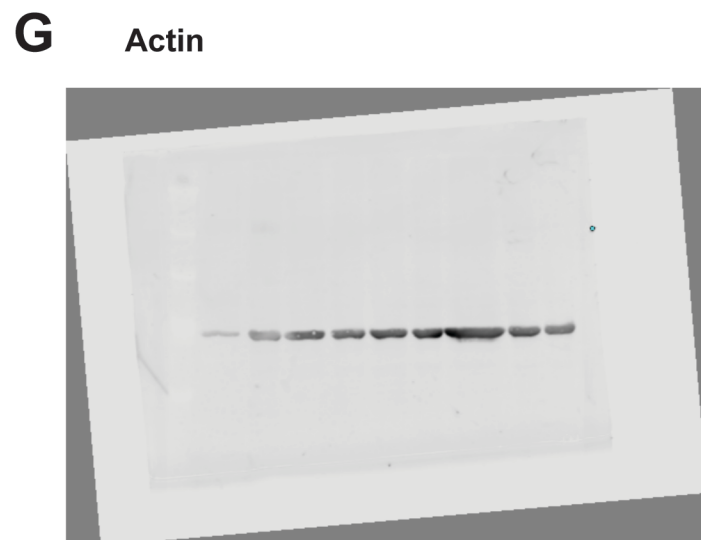

Supplement: Figure 5—figure supplement 1—source data 1. [file elife-50843-fig5-figsupp1-data1.pdf]

# Full Length Blots corresponding to Figure 5--Fig. Supp 1H

**A** Kv1.2

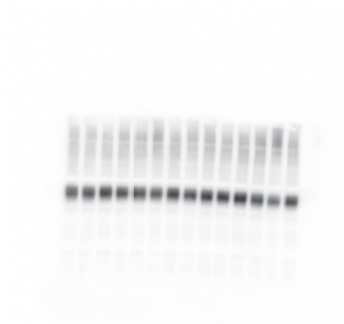

**B** PSD95

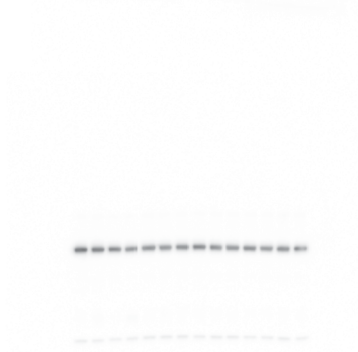

**C** Actin

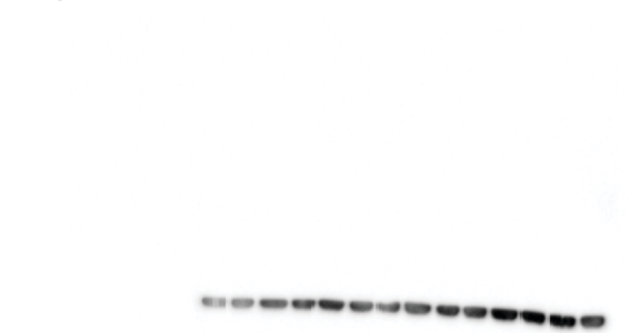

Supplement: Figure 5—figure supplement 1—source data 2. [file elife-50843-fig5-figsupp1-data2.pdf]

# Full Length Blots corresponding to Figure 5--Fig. Supp 2B

**A** Flag

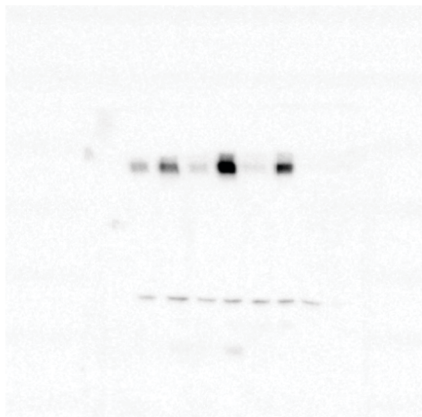

**C** LC3

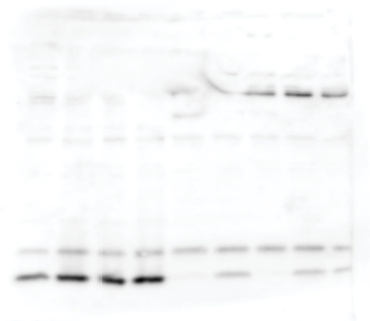

**B** Kir2.1

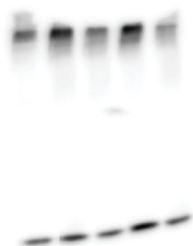

**D** Actin

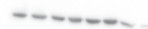

Supplement: Figure 5—figure supplement 2—source data 1. [file elife-50843-fig5-figsupp2-data1.pdf]

# Full Length Blots corresponding to Figure 5--Fig. Supp 3A

**A** Atg7

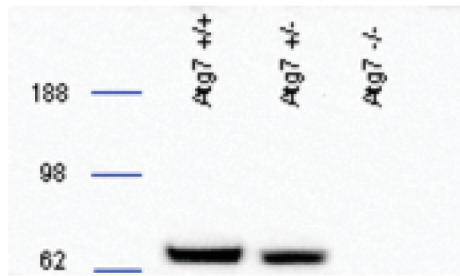

**B** Tubulin

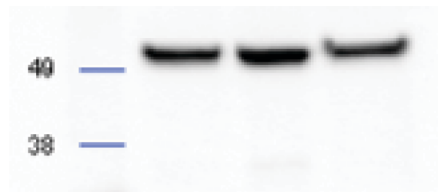

Supplement: Figure 5—figure supplement 3—source data 1. [file elife-50843-fig5-figsupp3-data1.pdf]

# Full Length Blots corresponding to Figure 7G-I

**A** PSD95

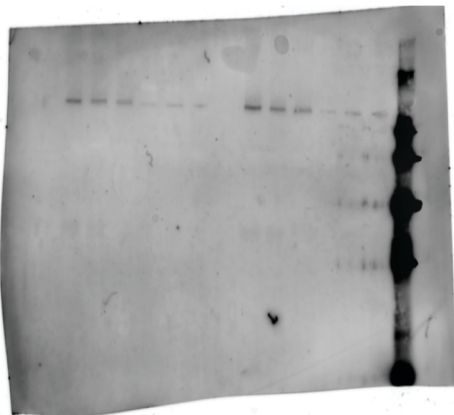

**B** DARPP32

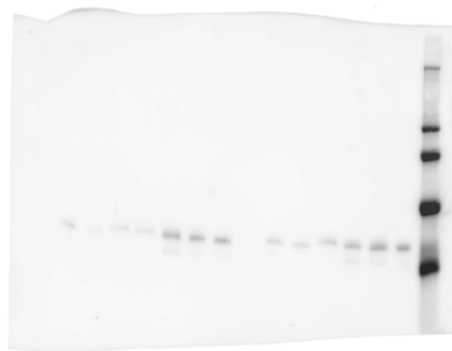

**C** Kir2.1

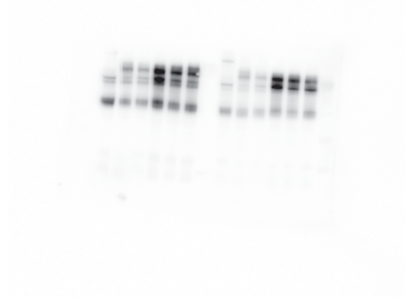

**D** Kir2.3

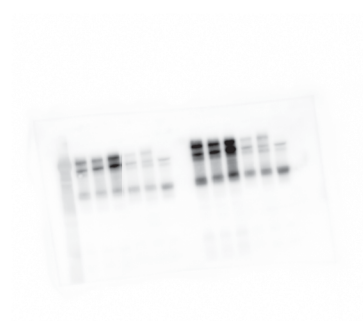

**E** p62

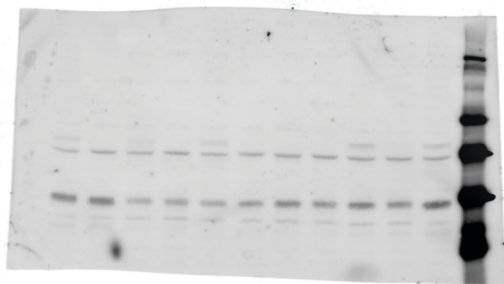

**F** Kv1.2

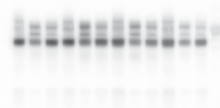

Supplement: Figure 7—source data 1. [file elife-50843-fig7-data1.pdf]

# Full Length Blots corresponding to Figure 8

**A** Ub

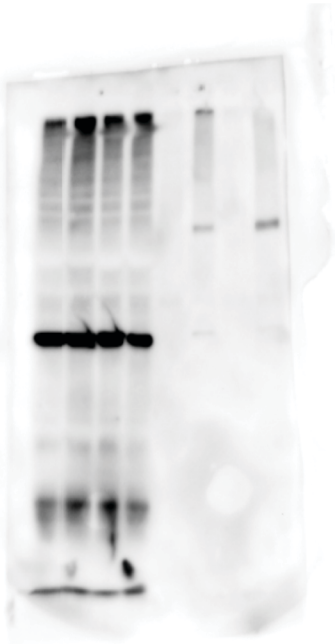

**B** AcLys

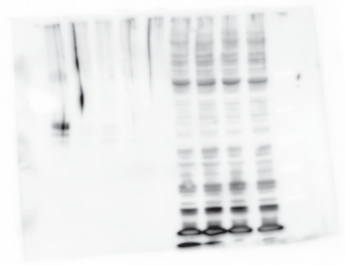

**C** Flag

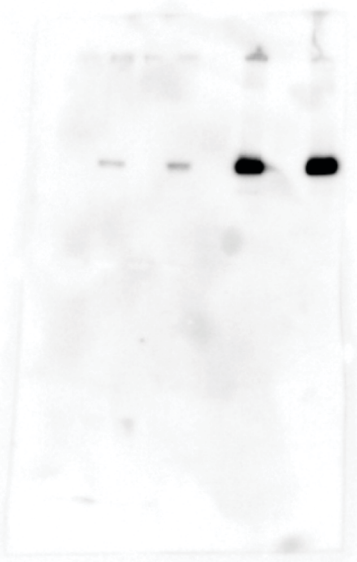

Supplement: Figure 8—source data 1. [file elife-50843-fig8-data1.pdf]
