# Supplementary material for: Cell-type-specific regulation of neuronal intrinsic excitability by macroautophagy
Source: eLife. 2020 Jan 8;9:e50843. doi: 10.7554/eLife.50843 (PMC6984822; doi:10.7554/eLife.50843)

Full Length Blots corresponding to Figure 5--Fig. Supp 2D

A Atg5^{WT} Flag

B Atg5^{WT} Actin

C Atg5^{KO} Flag

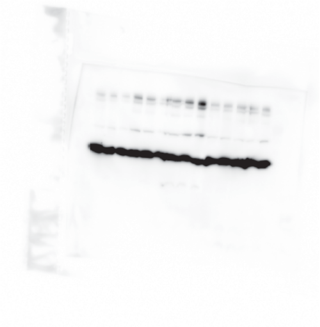

D Atg5^{KO} Actin

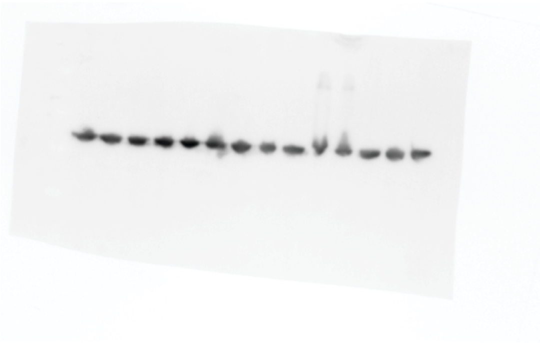

Supplement: Figure 5—figure supplement 2—source data 2. [file elife-50843-fig5-figsupp2-data2.pdf]
