# Supplementary material for: Cell-type-specific regulation of neuronal intrinsic excitability by macroautophagy
Source: eLife. 2020 Jan 8;9:e50843. doi: 10.7554/eLife.50843 (PMC6984822; doi:10.7554/eLife.50843)
Supplement: Source code 1. [file elife-50843-code1.rtf]

#pragma rtGlobals=1//	____________________________________________////			Amperometric Spike Analysis//			     Version 8.15 06/12/2005////				Eugene Mosharov, PhD//		Columbia University, Dpt. of Neurology//	em706@columbia.edu or johnmosh@yahoo.com//	____________________________________________//	To start: //	1. Hide or Minimize this window//	2. Choose * Prepare for Quanta Analysis *  from Macros menuMenu "Macros""-"	"* Prepare for Quanta Analysis! *",Prepare_for_Trace_Analysis()EndMacro Prepare_for_Trace_Analysis()	String Existing_windows=WinList("*", ";","WIN:83")	// A list of all windows, panels and notebooks	String One_win=StringFromList(0, Existing_windows)	If(strlen(One_win))		DoAlert 1,"Delete existing Windows?"		If(V_Flag==1)			Variable i=0			Do				One_win=StringFromList(i, Existing_windows)				If(strlen(One_win)==0)					break				endif				DoWindow/K $One_win				i+=1			while(1)		endif	endif	if (DataFolderExists("root:Quanta"))		SetDataFolder $"root:Quanta"	else		NewDataFolder/S root:Quanta	endif	//	Default values of global variables	if (CheckName("Bkg_noise_I", 3)==0)		Variable/G Bkg_noise_I=0					//	Standard Deviation of the noise on non-differentiated trace. Used during foot detection.	endif	if (CheckName("Bkg_noise_dI", 3)==0)		Variable/G Bkg_noise_dI=0				//	Standard Deviation of the noise on differentiated trace. Used for spike detection	endif	if (CheckName("Bkg_noise_Start", 3)==0)		Variable/G Bkg_noise_Start=0				//	Beginning of the segment used to calculate SD of the noise.	endif	if (CheckName("Bkg_noise_End", 3)==0)		Variable/G Bkg_noise_End=0				//	End of the segment used to calculate SD of the noise.	endif	if (CheckName("Detection_Mult", 3)==0)		Variable/G Detection_Mult=5				//	Theshold for spike detection	endif	if (CheckName("Detection_Foot_Mult", 3)==0)		Variable/G Detection_Foot_Mult=2			//	Theshold for foot detection	endif	if (CheckName("Smoothing_Factor", 3)==0)		Variable/G Smoothing_Factor=600			//	Cutoff of the filter used on non-differentiated trace. Can be changed from the main window.	endif	if (CheckName("Smoothing_Factor_diff1", 3)==0)		Variable/G Smoothing_Factor_diff1=300		//	Cutoff of Gaussian filter used on differentiated trace. Can be changed from Filters/Scales panel.	endif	if (CheckName("Smoothing_Factor_Add", 3)==0)		Variable/G Smoothing_Factor_Add=150	//	Cutoff of additional Gaussian filter used on non-differentiated trace. Can be changed from Filters/Scales panel.	endif	if (CheckName("Smooth_Derivative", 3)==0)		Variable/G Smooth_Derivative=1			//	Allows filtering of differentiated trace. 	endif	if (CheckName("Smooth_more", 3)==0)		Variable/G Smooth_more=0				//	Allows additional filtering of non-differentiated trace. 	endif	if (CheckName("Overall_Filter", 3)==0)		Variable/G Overall_Filter=0					//	Overall additive filtering (except additional) applied to non-differentiated trace. 	endif	if (CheckName("Spike_Min_Imax", 3)==0)		Variable/G Spike_Min_Imax=0				//	I(max) cutoff	endif	if (CheckName("Spike_Min_Imax_Last", 3)==0)		Variable/G Spike_Min_Imax_Last=3		//	Last used I(max) cutoff	endif	if (CheckName("Spike_Max_T05", 3)==0)		Variable/G Spike_Max_T05=0				//	t(1/2) cutoff	endif	if (CheckName("Spike_Max_T05_Last", 3)==0)		Variable/G Spike_Max_T05_Last=1			//	Last used t(1/2) cutoff	endif	if (CheckName("Spike_Max_Trise", 3)==0)		Variable/G Spike_Max_Trise=0				//	t(rise) cutoff		endif	if (CheckName("Spike_Max_Trise_Last", 3)==0)		Variable/G Spike_Max_Trise_Last=2		//	Last used t(rise) cutoff	endif	if (CheckName("Foot_Min_W", 3)==0)		Variable/G Foot_Min_W=0					//	T(foot) cutoff	endif	if (CheckName("Foot_Min_W_Last", 3)==0)		Variable/G Foot_Min_W_Last=2			//	Last used T(foot) cutoff	endif	if (CheckName("SSFoot_Do", 3)==0)		Variable/G SSFoot_Do=0					//	Delete feet without steady states	endif	if (CheckName("Native_Foot_Del", 3)==0)		Variable/G Native_Foot_Del=0				//	Delete feet longer that 0.33*t(rise 50-90%)	endif	if (CheckName("Foot_Min_H", 3)==0)		Variable/G Foot_Min_H=0					//	I(foot) cutoff	endif	if (CheckName("Foot_Min_H_Last", 3)==0)		Variable/G Foot_Min_H_Last=1			//	Last used I(foot) cutoff	endif	if (CheckName("Peak_ID", 3)==0)		Variable/G Peak_ID=0						//	The ID of the currently active spike	endif	if (CheckName("Total_peaks_number", 3)==0)		Variable/G Total_peaks_number=0			//	Total number of detected spikes	endif	if (CheckName("Rise_Low_Prc", 3)==0)		Variable/G Rise_Low_Prc=25				//	The lower point (% of Imax) on spike rising phase used to calculate r(rise).	endif	if (CheckName("Rise_Hi_Prc", 3)==0)		Variable/G Rise_Hi_Prc=75				//	The upper point (% of Imax) on spike rising phase used to calculate r(rise).	endif	if (CheckName("Fit_method", 4)==0)		String/G Fit_method="DblExp"				//	Function used to fit the falling phase of the spikes	endif	if (CheckName("Fall_ChiRatio_Cutoff", 3)==0)		variable/G Fall_ChiRatio_Cutoff=2			//	Chi^2(double exp) to Chi^2(single exp) ratio threshold for the DblExp fit. 	endif	if (CheckName("Gain", 3)==0)		variable/G Gain=1							//	Trace Y scaling gain.	endif	if (CheckName("Gain_Temp", 3)==0)		variable/G Gain_Temp=1					//	Trace Y scaling gain. Additional variable used to determine if the gain has been changed.	endif	if (CheckName("Zoom_On", 4)==0)		String/G Zoom_On="Off"					//	Zooms in and out of the currently active spike in the main window.	endif	if (CheckName("Show_Legend", 3)==0)		variable/G Show_Legend=1				//	Enables the legend on top of the main window.	endif	if (CheckName("T_Start_orig", 3)==0)		Variable/G T_Start_orig=0					//	X value of the first datapoint in the original trace.	endif	if (CheckName("T_Delta_orig", 3)==0)		variable/G T_Delta_orig=0.1				//	Sampling interval (microS) of the original trace.	endif	if (CheckName("T_Start", 3)==0)		Variable/G T_Start=0						//	X value of the first datapoint in the working trace copy.	endif	if (CheckName("T_End", 3)==0)		Variable/G T_End=0						//	X value of the last datapoint in the working trace copy.	endif	if (CheckName("T_Delta", 3)==0)		variable/G T_Delta=0.1					//	Sampling interval (microS) of the working trace copy.	endif	if (CheckName("X_min", 3)==0)		Variable/G X_min=0						//	X value of the first datapoint displayed in the main window.		endif	if (CheckName("X_max", 3)==0)		Variable/G X_max=0						//	X value of the last datapoint displayed in the main window.		endif	if (CheckName("Y_min", 3)==0)		Variable/G Y_min=0						//	Minimal Y value displayed in the main window.	endif	if (CheckName("Y_max", 3)==0)		Variable/G Y_max=0						//	Maximal Y value displayed in the main window.	endif	if (CheckName("Population_Center", 4)==0)		String/G Population_Center="Median"		//	Calculate a Mean or a Median of spike parameters during statistical analysis	endif	if (CheckName("Norm_point", 4)==0)		String/G Norm_point="Rise"				//	Uses spike Tmax ('Max') or the midpoint of the linear segment of the rising phase ('Rise') during spike averaging	endif	if (CheckName("Baseline_Drift", 3)==0)		Variable/G Baseline_Drift=50				//	Maximal allowed baseline drift. If higher, the spike is deleted.	endif	if (CheckName("Overlap_Prc", 3)==0)		Variable/G Overlap_Prc=50				//	Maximal allowed degree of spike overlap. If higher, both spikes are deleted.	endif	if (CheckName("Overlaps", 4)==0)		String/G Overlaps="Separate"				//	The remaining overlaps are either 'Ignored', 'Separated' or 'Split'.	endif	if (CheckName("File_list", 4)==0)		String/G File_list=""						//	The list of recently opened recordings	endif	if (CheckName("Loaded_file_path", 4)==0)		String/G Loaded_file_path=""				//	The name of the last opened recording	endif	if (CheckName("Fall_Tau_Extrap", 3)==0)		Variable/G Fall_Tau_Extrap=0				//	Exponential Tau used for falling phase extrapolasion of overlapping spikes.	endif	//	The list of parameters that will be displayed (not calculated) by default. To set new default settings change "0" to "1".	Variable/G Show_Time=1			//	T_Max	Variable/G Show_Base=0			//	Peak_Base	Variable/G Show_Width=1			//	Peak_t05	Variable/G Show_H=1				//	Peak_Imax	Variable/G Show_Q=0				//	Peak_Q	Variable/G Show_Molec=1			//	Peak_Molec	Variable/G Show_Rise_t=1		//	Rise_time	Variable/G Show_Rise_r=1		//	Rise_slope	Variable/G Show_Fall_t=1			//	Fall_time	Variable/G Show_Fall_r=1			//	Fall_slope	Variable/G Show_Ft_H=1			//	Foot_I	Variable/G Show_Ft_width=1		//	Foot_W	Variable/G Show_Ft_Q=0			//	Foot_Q	Variable/G Show_Ft_molec=1		//	Foot_Molec	String/G Values_to_show	String/G Stats_names	//	Build new windows	if (CheckName("Working_trace_copy", 1)==0)		Make/O/N=1 Working_trace_copy,Orig_trace_copy,diff1		Make/O/N=1 Zoomed_peak, Fall_phase,Rise_phase		Make/O/N=0 Avg_peak	endif	if (CheckName("Peak_Num", 1)==0)		Change_waves("Make",0)	endif	if (CheckName("File_Q", 6)==0)		File_Q()	else		Dowindow/F File_Q	endif	Variable Resolution=ScreenResolution	String scrn=StringByKey("SCREEN1", IgorInfo(0))	Variable Scrn_width=str2num(StringFromList(3, scrn,","))	Variable Scrn_hight=str2num(StringFromList(4, scrn,","))		Variable/G Scrn_width_Points=Scrn_width*0.74609375*96/Resolution	Variable/G Scrn_hight_Points= Scrn_hight*0.74609375*96/Resolution	Variable Zoom_Win_width=220*96/Resolution	Variable Table_Win_hight=180*96/Resolution	if (CheckName("Main_window", 6)==0)		execute "Main_window()"		SetVariable ID,limits={1,(Total_peaks_number),1}		PauseUpdate; Silent 1				Movewindow 0,80,Scrn_width_Points-Zoom_Win_width,Scrn_hight_Points-Table_Win_hight		AutoPositionWindow/E/M=1/R=File_Q Main_window		Dowindow/F File_Q		Show_Extras("Zoom_off")		MoveWindow /C Scrn_width_Points-Zoom_Win_width,320*96/Resolution,Scrn_width_Points,Scrn_hight_Points-75*96/Resolution	endif	if (CheckName("Peak_stats_Table1", 6)==0)		Change_Table ("ctrlName",1)		Dowindow/F Peak_stats_Table1		AutoPositionWindow/E/M=0/R=Main_window Peak_stats_Table1	endifEndmacro//___________________________________//_______Spike Detection and Analysis______//___________________________________Function Bkg_noise_Set(ctrlName) : ButtonControl	String ctrlname	SetDataFolder $"root:Quanta"	NVAR Bkg_noise_Start=Bkg_noise_Start	NVAR Bkg_noise_End=Bkg_noise_End	Wave Working_trace_copy=Working_trace_copy	If(numpnts(Working_trace_copy)<2)		abort "No trace loaded or the wave is too short."	endif	Bkg_noise_Start=min(xcsr(A), xcsr(B))	Bkg_noise_End=max(xcsr(A), xcsr(B))	Bkg_noise_Calc()EndFunction Bkg_noise_Calc()	SetDataFolder $"root:Quanta"	Wave Working_trace_copy=Working_trace_copy	NVAR Bkg_noise_I=Bkg_noise_I	NVAR Bkg_noise_dI=Bkg_noise_dI	NVAR Bkg_noise_Start=Bkg_noise_Start	NVAR Bkg_noise_End=Bkg_noise_End	NVAR Smoothing_Factor_diff1=Smoothing_Factor_diff1	NVAR Smooth_more=Smooth_more	NVAR Smooth_Derivative=Smooth_Derivative	NVAR Smoothing_Factor_Add=Smoothing_Factor_Add	If (Bkg_noise_Start==Bkg_noise_End)		String Note="Background level of noise is not set!\r" 		Note+="Use cursors A and B to select an area of your trace\r"		Note+="that does not have any spikes and press 'Bkg' button"		abort Note	endif	wavestats/Q/R = (Bkg_noise_Start, Bkg_noise_End ) Working_trace_copy	Bkg_noise_I=V_sdev	Duplicate/O/R=(Bkg_noise_Start,Bkg_noise_End) Working_trace_copy, Bkg_diff1	Variable Binomial_coeff	If (Smooth_more==1)		Binomial_coeff=Gaussian_to_Binomial_Calc(Smoothing_Factor_Add)		Smooth Binomial_coeff, Bkg_diff1	endif	Differentiate Bkg_diff1	If (Smooth_Derivative==1)		Binomial_coeff=Gaussian_to_Binomial_Calc(Smoothing_Factor_diff1)		Smooth Binomial_coeff, Bkg_diff1	endif	wavestats/Q Bkg_diff1	Bkg_noise_dI=V_sdev	Killwaves/Z Bkg_diff1	GroupBox Bkg_HiLt, win=Main_window,disable=1EndFunction Peak_finder(ctrlName) : ButtonControl	String ctrlname	SetDataFolder $"root:Quanta"	Wave Working_trace_copy=Working_trace_copy	NVAR Detection_Mult=Detection_Mult	NVAR Total_peaks_number=Total_peaks_number	NVAR Peak_ID=Peak_ID	NVAR Bkg_noise_dI=Bkg_noise_dI	NVAR Bkg_noise_Start=Bkg_noise_Start	NVAR Bkg_noise_End=Bkg_noise_End	NVAR Smoothing_Factor=Smoothing_Factor	NVAR Smoothing_Factor_diff1=Smoothing_Factor_diff1	NVAR Smooth_Derivative=Smooth_Derivative	NVAR Smooth_more=Smooth_more	NVAR Smoothing_Factor_Add=Smoothing_Factor_Add	NVAR Baseline_Drift=Baseline_Drift	Wave Peak_Num=Peak_Num	Wave T_Max=T_Max	Wave Peak_Imax=Peak_Imax	Wave Peak_Half_H2=Peak_Half_H2	Wave Peak_Q=Peak_Q	Wave T_Bkg1=T_Bkg1	Wave T_Bkg2=T_Bkg2	Wave Working_trace_copy=Working_trace_copy	If(numpnts(Working_trace_copy)<2)		abort "No trace loaded or the wave is too short."	endif		If(Total_peaks_number)		DoAlert 1, "All existing spikes will be deleted! \rPress 'Yes' to continue or 'No' to cancel."		If (V_Flag==1)			De_novo()		else			abort		endif			endif	Bkg_noise_Calc()	Getaxis/Q bottom	Variable Left_X=V_min	Variable Start_X=V_min	Variable End_X=min(V_max, pnt2x(Working_trace_copy,(numpnts(Working_trace_copy)-1)))	if (CheckName("Peak_stats_Table1", 6)==0)		execute "Peak_stats_Table()"	else		Dowindow/F Peak_stats_Table1		MoveWindow 1, 1, 1, 1	endif	Variable Binomial_coeff,Real_cutoff	If (Smooth_more==1)		Duplicate/O/R=(Start_X,End_X) Working_trace_copy, diff1, diff1_nosmooth		Binomial_coeff=Gaussian_to_Binomial_Calc(Smoothing_Factor_Add)		Smooth Binomial_coeff, diff1		Real_cutoff=Binomial_to_Gaussian_Calc(Binomial_coeff)		Print "Trace was additionally filtered with "+num2str(Real_cutoff)+"Hz (Binomial "+num2str(Binomial_coeff)+") -3dB Gaussian filter."	else		Duplicate/O/R=(Start_X,End_X) Working_trace_copy, diff1, diff1_nosmooth	endif	Differentiate diff1, diff1_nosmooth	If (Smooth_Derivative==1)		Binomial_coeff=Gaussian_to_Binomial_Calc(Smoothing_Factor_diff1)		Smooth Binomial_coeff, diff1, diff1_nosmooth	endif	Variable Detection_level_diff1=Bkg_noise_dI*Detection_Mult	Total_peaks_number=0		Variable Kapec=0	Variable dI_zero_L, dI_zero_R,Limit_R,Limit_L	do		If (cmpnum(Start_X,End_X,18)!=0)			FindPeak/Q/M=(Detection_level_diff1)/R=(Start_X,End_X) diff1		else			Kapec=1		endif		if ((V_Flag==0)%&(Kapec!=1))			If(V_TrailingEdgeLoc>0)				Total_peaks_number+=1				Change_waves("Redimension",Total_peaks_number)				Peak_Num[Total_peaks_number-1]=Total_peaks_number				Start_X=V_PeakLoc				dI_zero_R=V_TrailingEdgeLoc				dI_zero_L=V_PeakLoc				Limit_L=max(Left_X,T_Bkg2[Total_peaks_number-2])								//	temporary Tmax				FindLevel/Q/R=(Start_X,End_X) diff1, 0				T_Max[Total_peaks_number-1]=V_LevelX								//	next spike Tmax or the end of the trace				FindPeak/Q/M=(Detection_level_diff1)/R=(T_Max[Total_peaks_number-1],End_X) diff1				if (V_Flag==0)					Limit_R=V_PeakLoc				else					Limit_R=End_X				endif				//	final Tmax				FindLevel/Q/R=(T_Max[Total_peaks_number-1],Limit_R) Working_trace_copy, Working_trace_copy(dI_zero_L)				If(V_flag==0)					dI_zero_R=V_LevelX					wavestats/Q/R=(dI_zero_L,dI_zero_R) Working_trace_copy					T_Max[Total_peaks_number-1]=V_maxloc				endif				Cursor /W=Main_window A Working_trace_copy T_Max[Total_peaks_number-1]				//	temporary Tbkg1				FindLevel/Q/R=(dI_zero_L,Limit_L) diff1, 0				If(V_flag==1)					T_Bkg1[Total_peaks_number-1]=Limit_L				else					T_Bkg1[Total_peaks_number-1]=max(V_LevelX,T_Bkg2[Total_peaks_number-2])				endif				//	final Tbkg1				Variable SteadyState=Find_SteadyState(dI_zero_L, Limit_L, 2*(dI_zero_R-dI_zero_L))				If(SteadyState)					Variable SteadyState4=Find_SteadyState(dI_zero_L, Limit_L, 4*(dI_zero_R-dI_zero_L))					If(SteadyState4)						FindLevel/Q/R=(dI_zero_L,Limit_L) Working_trace_copy, SteadyState4					else						FindLevel/Q/R=(dI_zero_L,Limit_L) Working_trace_copy, SteadyState					endif					T_Bkg1[Total_peaks_number-1]=V_LevelX				endif								Start_X=dI_zero_R				//	Tbkg2				FindLevel /Q/R=(T_Max[Total_peaks_number-1],Limit_R) diff1, 0				If (V_Flag)					Peak_Q[Total_peaks_number-1]=0				else					T_Bkg2[Total_peaks_number-1]=V_LevelX										Variable Flat_Peak_End=Find_level_plus_2SD(T_Max[Total_peaks_number-1],Limit_R,T_Bkg1[Total_peaks_number-1])					If(Flat_Peak_End)						T_Bkg2[Total_peaks_number-1]=max(Flat_Peak_End,T_Bkg2[Total_peaks_number-1])					else						wavestats/Q/R=(T_Max[Total_peaks_number-1],Limit_R) Working_trace_copy						T_Bkg2[Total_peaks_number-1]=V_minloc					endif					If ((T_Max[Total_peaks_number-1]-T_Max[Total_peaks_number-2]>0)%|(Total_peaks_number==1))						Calc_Peak_Parameters(Total_peaks_number,T_Bkg1[Total_peaks_number-1],T_Bkg2[Total_peaks_number-1])					else						Peak_Q[Total_peaks_number-1]=0					endif					Wavestats/Q Peak_Q					If(V_numNans!=0)						Peak_Q[Total_peaks_number-1]=0					endif					If ((T_Bkg1[Total_peaks_number-1]>=T_Max[Total_peaks_number-1])%|(T_Bkg2[Total_peaks_number-1]<=T_Max[Total_peaks_number-1]))						Peak_Q[Total_peaks_number-1]=0					endif										//	Check for spikes on uneven baseline					Variable Spike_Min_Imax=Working_trace_copy(T_Max[Total_peaks_number-1])-max(Working_trace_copy(T_Bkg1[Total_peaks_number-1]),Working_trace_copy(T_Bkg2[Total_peaks_number-1]))					Variable Spike_Max_Imax=Working_trace_copy(T_Max[Total_peaks_number-1])-min(Working_trace_copy(T_Bkg1[Total_peaks_number-1]),Working_trace_copy(T_Bkg2[Total_peaks_number-1]))					If(Spike_Min_Imax<(Spike_Max_Imax*(100-Baseline_Drift)/100))						Peak_Q[Total_peaks_number-1]=0					endif				endif				Variable Next_Start_X=0				If(Detection_limits(Total_peaks_number))					If(Total_peaks_number>1)						Next_Start_X=Peak_Half_H2[Total_peaks_number-1]					endif					Change_waves("Delete",(Total_peaks_number-1))					Total_peaks_number=Total_peaks_number-1				endif				Start_X=max(Start_X,Next_Start_X)			else				Start_X=V_PeakLoc			endif		else				SVAR Overlaps			if((cmpstr(Overlaps,"Ignore")!=0)&(Total_peaks_number>1))				Check_for_Overlaps()			endif			if(Total_peaks_number==0)				Peak_ID=0				SetVariable ID,limits={0,0,0},win=Main_window				if (CheckName("Zoom_Win", 6)!=0)					SetVariable ID,limits={0,0,0},win=Zoom_Win				endif				SetDrawLayer /K UserFront				abort "No amperometric events found!"			else				Peak_ID=1				SetVariable ID,limits={1,(Total_peaks_number),1},win=Main_window				if (CheckName("Zoom_Win", 6)!=0)					SetVariable ID,limits={1,(Total_peaks_number),1},win=Zoom_Win				endif				Peak_locator(0)				print "Found "+num2str(Total_peaks_number)+" spikes."				Abort 			endif		endif	while (1)EndFunction Check_for_Overlaps()	SetDataFolder $"root:Quanta"	Wave Working_trace_copy=Working_trace_copy	Wave T_Max=T_Max	Wave Peak_Num=Peak_Num	Wave Peak_Split1=Peak_Split1	Wave Peak_Split2=Peak_Split2	Wave T_Bkg1=T_Bkg1	Wave T_Bkg2=T_Bkg2	Wave Peak_t05=Peak_t05	Wave/T Fall_fit=Fall_fit	NVAR Overlap_Prc=Overlap_Prc	SVAR Overlaps=Overlaps	NVAR Bkg_noise_I=Bkg_noise_I	NVAR Total_peaks_number=Total_peaks_number	//	Two spikes are considered overlapping if the distance between the end of the 1st spike and the beginning of the 2nd one is less than the average t1/2 * 2.	Variable i=1, ii=1	Variable Last_overlapping_peak	Variable Peak_limit	Variable Same_level	Make/O/N=(Total_peaks_number) Peak_Delta	Peak_Delta[1,]=((T_Bkg1[p]-T_Bkg2[p-1])-2*(mean(Peak_t05, 0, Total_peaks_number)/1000))*1000	do		if((Working_trace_copy(T_Bkg2[i-1])>(Working_trace_copy(T_Bkg1[i-1])+2*Bkg_noise_I))&(Peak_Delta[i]<0))			FindLevel /P/Q/R=[i,Total_peaks_number] Peak_Delta, 0			If(V_LevelX)				Last_overlapping_peak=trunc(V_LevelX)+1			else				Last_overlapping_peak=Total_peaks_number			endif			If((Last_overlapping_peak+1)<=Total_peaks_number)				Peak_limit=T_Bkg1[Last_overlapping_peak]			else				Getaxis/W=Main_Window/Q bottom				Peak_limit=min(V_max, pnt2x(Working_trace_copy,(numpnts(Working_trace_copy)-1)))			endif			Same_level=Find_level_plus_2SD(T_Max[Last_overlapping_peak-1],Peak_limit,T_Bkg1[i-1])			Variable Final_level			if(Same_level)				Final_level=Same_level			else				Final_level=T_Bkg2[Last_overlapping_peak-1]			endif			T_Bkg2[i-1,Last_overlapping_peak-1]=Final_level			ii=i-1			Variable Seddle, Min_Tmax, Kill_it=0			do				Wavestats/Q/R=(T_max[ii],T_max[ii+1]) Working_trace_copy				Seddle=V_min-Working_trace_copy(Final_level)				Min_Tmax=min(Working_trace_copy(T_max[ii])-Working_trace_copy(Final_level),Working_trace_copy(T_max[ii+1])-Working_trace_copy(Final_level))				If((Seddle/Min_Tmax*100)>Overlap_Prc)					Kill_it=1				endif				ii+=1			while (ii<Last_overlapping_peak-1)			if((cmpstr(Overlaps,"Delete")==0)%|(Kill_it))				print Last_overlapping_peak-(i-1),"overlapping spikes at",T_Max[i-1],"were deleted"				ii=i-1				Do					Change_waves("Delete",i-1)					DeletePoints i-1,1,Peak_Delta					Peak_Num[i-1,]-=1					ii+=1					Total_peaks_number-=1				while(ii<Last_overlapping_peak)				i-=Last_overlapping_peak-(i-1)			endif			if((cmpstr(Overlaps,"Separate")==0)&(Kill_it==0))				ii=i//				Fall_fit_Extrap[ii-1]="1"				Do					Fall_fit[ii]=""//					Wave/T Fall_fit_Extrap=Fall_fit_Extrap//					Fall_fit_Extrap[ii]=num2str(2+ii-i)					WaveStats/Q/R = (T_Max[ii-1],T_Max[ii]) Working_trace_copy					T_Bkg1[ii]=V_minloc					ii+=1				while(ii<=Last_overlapping_peak-1)								Calc_Separated_peak_param(i-1,Last_overlapping_peak-1)				print "Overlapping spikes",i,"to",Last_overlapping_peak,"were Separated."				i=Last_overlapping_peak			endif			if((cmpstr(Overlaps,"Split")==0)&(Kill_it==0))				T_Bkg1[i-1,Last_overlapping_peak-1]=T_Bkg1[i-1]				ii=i				Do					Fall_fit[ii]=""					Fall_fit[ii-1]=""					WaveStats/Q/R = (T_Max[ii-1],T_Max[ii]) Working_trace_copy					Peak_Split2[ii-1]=V_minloc					Peak_Split1[ii]=V_minloc					Calc_split_peak_param(ii-1)					Calc_split_peak_param(ii)					ii+=1				while(ii<Last_overlapping_peak)				print "Overlapping spikes",i,"to",Last_overlapping_peak,"were Split."				i=Last_overlapping_peak			endif		endif		i+=1	while(i<=Total_peaks_number)	Killwaves/Z Peak_Delta	String Extrap_	String Extrap_waves=WaveList("Extrap*",";","")	ii=0	Do		Extrap_=StringFromList(ii,Extrap_waves)		If(strlen(Extrap_)==0)			break		endif		AppendToGraph/W=Main_window $Extrap_		ModifyGraph lstyle($Extrap_)=2,lsize($Extrap_)=0.5,rgb($Extrap_)=(0,15872,65280)		ii+=1	while(1)	if(numpnts(Peak_Num))		Wavestats/Q Peak_Num		Total_peaks_number=V_npnts	else		Total_peaks_number=0	endifEndFunction Find_level_plus_2SD(StartX,LimitX,LevelY)	Variable StartX,LimitX,LevelY	SetDataFolder $"root:Quanta"	Wave Working_trace_copy=Working_trace_copy	NVAR Bkg_noise_I=Bkg_noise_I	NVAR Smoothing_Factor=Smoothing_Factor	Duplicate/O/R=(StartX,LimitX) Working_trace_copy, TEMP_wave	Variable Binomial_coeff=Gaussian_to_Binomial_Calc(Smoothing_Factor/2)	Smooth Binomial_coeff, TEMP_wave	FindLevel/Q/R=(StartX,LimitX) TEMP_wave, Working_trace_copy(LevelY)	if (V_LevelX)		FindLevel/Q/R=(V_LevelX,LimitX) Working_trace_copy, Working_trace_copy(LevelY)	endif	if (V_Flag)		FindLevel/Q/R=(StartX,LimitX) TEMP_wave, (Working_trace_copy(LevelY)+Bkg_noise_I)		if (V_Flag)			FindLevel/Q/R=(StartX,LimitX) TEMP_wave, (Working_trace_copy(LevelY)+2*Bkg_noise_I)		endif	endif	KillWaves/Z TEMP_wave	if (V_Flag)		Return 0	else			Return V_LevelX	endifEndFunction Add_Peak_Manually(ctrlName) : ButtonControl	String ctrlname	SetDataFolder $"root:Quanta"	NVAR Total_peaks_number=Total_peaks_number	NVAR Peak_ID=Peak_ID	Wave Peak_Num=Peak_Num	Wave T_Max=T_Max	Wave Working_Trace_Copy=Working_Trace_Copy	If(numpnts(Working_trace_copy)<2)		abort "No trace loaded or the wave is too short."	endif	Variable Start_X=min(xcsr(A),xcsr(B))	Variable End_X=max(xcsr(A),xcsr(B))	Variable New_T_Max=Find_One_Peak(Start_X,End_X)	If (New_T_Max==0)		abort	endif		Variable New_peak_pnt=New_peak_position(New_T_Max)	If (New_peak_pnt<0)		If(New_peak_pnt==-0.5)			New_peak_pnt=0		else			New_peak_pnt=abs(New_peak_pnt)		endif		If (Total_peaks_number!=1)					//	it is not the very first peak			Peak_ID=New_peak_pnt+1		endif		abort "Spike with this time at maximum already exists!"	endif	If (T_Max[Total_peaks_number-1]!=0)				//	it is not the very first peak		Change_waves("Insert",New_peak_pnt)	endif	Peak_Num[]=p+1	T_Max[New_peak_pnt]=New_T_Max	Wavestats/Q Peak_Num	Total_peaks_number=V_npnts	FindLevel /Q/R=(T_Max[New_peak_pnt],Start_X ) Working_Trace_Copy, Working_Trace_Copy(Start_X)	If(V_flag==0)		Start_X=V_LevelX	endif	Calc_Peak_Parameters(New_peak_pnt,Start_X,End_X)	Peak_ID=New_peak_pnt+1	SetVariable ID,limits={1,(Total_peaks_number),1},win=Main_window	if (CheckName("Zoom_Win", 6)!=0)		SetVariable ID,limits={1,(Total_peaks_number),1},win=Zoom_Win	endif	Draw_lines_All(New_peak_pnt)EndFunction Find_One_Peak(Start_X, End_X)	Variable Start_X	Variable End_X	Variable Delta_X=End_X-Start_X	SetDataFolder $"root:Quanta"	NVAR Detection_Mult=Detection_Mult	NVAR Smoothing_Factor_diff1=Smoothing_Factor_diff1	NVAR Bkg_noise_dI=Bkg_noise_dI	NVAR Smooth_more=Smooth_more	NVAR Smooth_Derivative=Smooth_Derivative	NVAR Smoothing_Factor_Add=Smoothing_Factor_Add	Wave diff1=diff1	Wave Working_trace_copy=Working_trace_copy	Bkg_noise_Calc()	Variable Binomial_coeff	If (Smooth_more==1)		If((x2pnt(Working_trace_copy,(End_X+3*Delta_X))-x2pnt(Working_trace_copy,(Start_X-3*Delta_X)))<Smoothing_Factor_Add)			DoAlert 0, "Smoothing of the trace requires more datapoints.\rReduce Additional Smoothing factor (Detection panel)."			Return 0		endif		Duplicate/O/R=((Start_X-3*Delta_X),(End_X+3*Delta_X)) Working_trace_copy, diff1, diff1_nosmooth		Binomial_coeff=Gaussian_to_Binomial_Calc(Smoothing_Factor_Add)		Smooth Binomial_coeff, diff1	else		Duplicate/O/R=((Start_X-3*Delta_X),(End_X+3*Delta_X)) Working_trace_copy, diff1, diff1_nosmooth	endif	Differentiate diff1, diff1_nosmooth	If (Smooth_Derivative==1)		Binomial_coeff=Gaussian_to_Binomial_Calc(Smoothing_Factor_diff1)		Smooth Binomial_coeff, diff1, diff1_nosmooth	endif		Variable Detection_level_diff1=Bkg_noise_dI*Detection_Mult	Variable New_T_Max	wavestats/Q/R=(Start_X,End_X) Working_trace_copy	New_T_Max=V_maxloc	FindPeak/Q/M=(Detection_level_diff1)/R=(Start_X,End_X) diff1	if (V_Flag)		String Message="No events found between the coursors. \rTry reducing detection threshold and then repeate the detection.\rIf you want to add this spike anyway, press 'Yes'. "		DoAlert 1,Message		If (V_Flag==1)			Return New_T_Max		else			Return 0		endif			else		FindLevel /Q/R=(V_PeakLoc,xcsr(B)) diff1, 0		if (V_Flag)			Message="This spike does not return to the baseline level. \rReduce the 'Smooth for 1st derrivative' factor under 'Options' menu.\rIf you want to add the spike anyway, press 'Yes'. "			DoAlert 1,Message			If (V_Flag==1)				Return New_T_Max			else				Return 0			endif		else			Return New_T_Max		endif	endifEndFunction Split(ctrlName) : ButtonControl	String ctrlname	SetDataFolder $"root:Quanta"	NVAR Total_peaks_number=Total_peaks_number	If(Total_peaks_number==0)		abort 	endif			Wave Peak_Num=Peak_Num	Wave T_Max=T_Max	Wave T_Bkg1=T_Bkg1	Wave T_Bkg2=T_Bkg2	Wave Peak_Split1=Peak_Split1	Wave Peak_Split2=Peak_Split2	NVAR Peak_ID=Peak_ID	Variable Peak_pnt=Peak_ID-1	NVAR Detection_Mult=Detection_Mult	NVAR Smoothing_Factor_diff1=Smoothing_Factor_diff1	Wave Working_trace_copy=Working_trace_copy	Variable L=max(T_Bkg1[Peak_pnt], Peak_Split1[Peak_pnt])	Variable R	If (Peak_Split2[Peak_pnt]!=0)		R=Peak_Split2[Peak_pnt]	else		R=T_Bkg2[Peak_pnt]	endif		If ((xcsr(A)<=L)%|(xcsr(A)>=R))		abort "Splitting point (round coursor) has to be between the Start and the End of the spike."	endif	Variable Split_pnt=xcsr(A)	String Split_Where	Variable Common_bkg1=T_Bkg1[Peak_pnt]	Variable Common_bkg2=T_Bkg2[Peak_pnt]	Variable Start_X, End_X	If (T_Max[Peak_pnt]<Split_pnt)		If (Peak_Split2[Peak_pnt]!=0)			Split_Where="Right_Mid"			End_X=Peak_Split2[Peak_pnt]		else			Split_Where="Right_Last"			End_X=T_Bkg2[Peak_pnt]		endif		Start_X=Split_pnt	else			If (Peak_Split1[Peak_pnt]!=0)			Split_Where="Left_Mid"			Start_X=Peak_Split1[Peak_pnt]		else			Split_Where="Left_First"			Start_X=T_Bkg1[Peak_pnt]		endif		End_X=Split_pnt	endif	Variable New_T_Max	WaveStats /Q /R = (Start_X, End_X ) Working_trace_copy	New_T_Max=V_maxloc	If(New_T_Max==0)		abort	endif		Variable New_peak_pnt=New_peak_position(New_T_Max)	If (New_peak_pnt<0)		If(New_peak_pnt==-0.5)			New_peak_pnt=0		else			New_peak_pnt=abs(New_peak_pnt)		endif		Peak_ID=New_peak_pnt+1		abort "Spike with this time already exists!"	endif	If (T_Max[Peak_pnt]<Split_pnt)		Peak_Split2[Peak_pnt]=Split_pnt	else			Peak_Split1[Peak_pnt]=Split_pnt	endif	Change_waves("Insert",New_peak_pnt)	Peak_Num[]=p+1	T_Max[New_peak_pnt]=New_T_Max	T_Bkg1[New_peak_pnt]=Common_bkg1	T_Bkg2[New_peak_pnt]=Common_bkg2		If (strsearch(Split_Where,"Right",0)!=-1)		Peak_Split1[New_peak_pnt]=Split_pnt		If (cmpstr(Split_Where,"Right_Mid")==0)			Peak_Split2[New_peak_pnt]=Peak_Split1[New_peak_pnt+1]		endif	endif	If (strsearch(Split_Where,"Left",0)!=-1)		Peak_Split2[New_peak_pnt]=Split_pnt		If (cmpstr(Split_Where,"Left_Mid")==0)			Peak_Split1[New_peak_pnt]=Peak_Split2[New_peak_pnt-1]		endif	endif	//	recalculate parameters for the original spike	If (strsearch(Split_Where,"Right",0)!=-1)		Calc_split_peak_param(New_peak_pnt-1)	else		Calc_split_peak_param(New_peak_pnt+1)	endif	//	calculate parameters for the new spike	Calc_split_peak_param(New_peak_pnt)	Peak_ID=New_peak_pnt+1	Wavestats/Q Peak_Num	Total_peaks_number=V_npnts	SetVariable ID,limits={1,(Total_peaks_number),1},win=Main_window	if (CheckName("Zoom_Win", 6)!=0)		SetVariable ID,limits={1,(Total_peaks_number),1},win=Zoom_Win	endif	Draw_lines_All(New_peak_pnt)EndFunction Calc_Split_Peak_Param(Peak_pnt)	Variable Peak_pnt	SetDataFolder $"root:Quanta"	Wave Peak_Half_H1=Peak_Half_H1	Wave Peak_Half_H2=Peak_Half_H2	Wave T_Max=T_Max	Wave Peak_Base=Peak_Base	Wave Peak_Imax=Peak_Imax	Wave T_Bkg1=T_Bkg1	Wave T_Bkg2=T_Bkg2	Wave Peak_Split1=Peak_Split1	Wave Peak_Split2=Peak_Split2	Wave Peak_t05=Peak_t05	Wave Peak_Q=Peak_Q	Wave Peak_Molec=Peak_Molec	Wave Working_trace_copy=Working_trace_copy	Variable Start_X=T_Bkg1[Peak_pnt]	Variable End_X=T_Bkg2[Peak_pnt]	Variable Max_X=T_Max[Peak_pnt]	Variable Bkg_under_the_Max=Working_trace_copy(Start_X)+(Working_trace_copy(End_X)-Working_trace_copy(Start_X))*(Max_X-Start_X)/(End_X-Start_X)	Variable Half_Height=Working_trace_copy(Max_X) - (Working_trace_copy(Max_X)-Bkg_under_the_Max)/2	Peak_Imax[Peak_pnt]=Working_trace_copy(Max_X)-Bkg_under_the_Max	Start_X=max(T_Bkg1[Peak_pnt], Peak_Split1[Peak_pnt])	FindLevel /Q/R=(Start_X,Max_X) Working_trace_copy, Half_Height	If(V_Flag==1)		Peak_Half_H1[Peak_pnt]=Peak_Split1[Peak_pnt]	else		Peak_Half_H1[Peak_pnt]=min(V_LevelX,Max_X)	endif						If (Peak_Split2[Peak_pnt]!=0)		End_X=Peak_Split2[Peak_pnt]	else		End_X=T_Bkg2[Peak_pnt]	endif	FindLevel /Q/R=(Max_X, End_X) Working_trace_copy, Half_Height	If (V_flag==1)		Peak_Half_H2[Peak_pnt]=End_X	else		Peak_Half_H2[Peak_pnt]=max(V_LevelX,Max_X)	endif	Peak_Base[Peak_pnt]=(End_X-Start_X)*1000	Peak_t05[Peak_pnt]=(Peak_Half_H2[Peak_pnt]-Peak_Half_H1[Peak_pnt])*1000	Variable Area_total=area(Working_trace_copy,Start_X,End_X)	Variable Bkg1_Y, Bkg2_Y	If (Peak_Split1[Peak_pnt]!=0)		Bkg1_Y=Y_offset(T_Bkg1[Peak_pnt], T_Bkg2[Peak_pnt], Peak_Split1[Peak_pnt])	else		Bkg1_Y=Working_trace_copy(T_Bkg1[Peak_pnt])	endif	If (Peak_Split2[Peak_pnt]!=0)		Bkg2_Y=Y_offset(T_Bkg1[Peak_pnt], T_Bkg2[Peak_pnt], Peak_Split2[Peak_pnt])	else		Bkg2_Y=Working_trace_copy(T_Bkg2[Peak_pnt])	endif	Variable Area_bkg=(End_X-Start_X)*(Bkg1_Y+Bkg2_Y)/2	Peak_Q[Peak_pnt]=Area_total-Area_bkg	Peak_Molec[Peak_pnt]=(Peak_Q[Peak_pnt])*3.121*10^6	Calc_Rise_Fall(Peak_pnt)EndFunction/S Check_for_Separated_peaks(Peak_pnt)	Variable Peak_pnt	SetDataFolder $"root:Quanta"	Wave T_Max=T_Max	Wave Peak_Imax=Peak_Imax	Wave T_Bkg1=T_Bkg1	Wave T_Bkg2=T_Bkg2	Wave Peak_Split1=Peak_Split1	Wave Peak_Split2=Peak_Split2	Wave Working_trace_copy=Working_trace_copy	NVAR Total_peaks_number=Total_peaks_number	Variable Start_X=T_Bkg1[Peak_pnt]	Variable End_X=T_Bkg2[Peak_pnt]	Variable Max_X=T_Max[Peak_pnt]		Variable First_Overlapp_peak=0,Last_Overlapp_peak=0	If ((Peak_Split2[Peak_pnt]!=0)%|(Peak_Split1[Peak_pnt]!=0))		return "0;0"	endif	If ((T_Max[Peak_pnt]!=T_Max[Peak_pnt+1])&(T_Max[Peak_pnt+1])<(T_Bkg2[Peak_pnt]))		First_Overlapp_peak=Peak_pnt	endif	Variable i=Peak_pnt-1	Do 		If ((T_Max[Peak_pnt]!=T_Max[i])&(T_Max[Peak_pnt])<(T_Bkg2[i]))			First_Overlapp_peak=i		else			break		endif		i-=1	while (i>=0)	Variable ii	ii=First_Overlapp_peak+1	Do 		If (T_Max[ii]<T_Bkg2[First_Overlapp_peak])			Last_Overlapp_peak=ii		else			break		endif		ii+=1	while (ii<=Total_peaks_number-1)		if(Total_peaks_number<2)		return "0;0"	endif		If((First_Overlapp_peak!=Last_Overlapp_peak)&(T_Bkg2[First_Overlapp_peak]>T_Max[Peak_pnt]))		String First_and_Last=num2str(First_Overlapp_peak)+";"+num2str(Last_Overlapp_peak)		return First_and_Last	else		return "0;0"	endifEndFunction Calc_Separated_Peak_Param(First_Overlapp_pnt,Last_Overlapp_pnt)	Variable First_Overlapp_pnt	Variable Last_Overlapp_pnt	SetDataFolder $"root:Quanta"	Wave Peak_Half_H1=Peak_Half_H1	Wave Peak_Half_H2=Peak_Half_H2	Wave T_Max=T_Max	Wave Peak_Base=Peak_Base	Wave Peak_Imax=Peak_Imax	Wave T_Bkg1=T_Bkg1	Wave T_Bkg2=T_Bkg2	Wave Peak_Split1=Peak_Split1	Wave Peak_Split2=Peak_Split2	Wave Peak_t05=Peak_t05	Wave Peak_Q=Peak_Q	Wave Peak_Molec=Peak_Molec	Wave Fall_slope=Fall_slope	Wave Fall_slope2=Fall_slope2	Wave Working_trace_copy=Working_trace_copy	Wave/T Fall_Fit=Fall_Fit	NVAR Total_peaks_number=Total_peaks_number	SVAR Fit_method=Fit_method	Wave/T Fall_fit_Extrap=Fall_fit_Extrap		String Extrapolation_name=""	Variable A0, A1, tau1,Slope	String Extrap_Exists	Variable Half_Height, Max_X, Start_X, End_X, End_Common	Variable Area_total, Area_bkg, Area_Extra=0	End_Common=T_Bkg2[First_Overlapp_pnt]		Variable i=First_Overlapp_pnt								//	First peak pnt	Do		If(cmpstr(Fall_fit_Extrap[i],"")==0)			Fall_fit_Extrap[i]="N:;Total:;Fit:;Tau:"		endif				If(cmpstr(StringByKey("N", Fall_fit_Extrap[i]),"")==0)			Fall_fit_Extrap[i] = ReplaceStringByKey("N", Fall_fit_Extrap[i], num2str(1+i-First_Overlapp_pnt))			Fall_fit_Extrap[i] = ReplaceStringByKey("Total", Fall_fit_Extrap[i], num2str(1+Last_Overlapp_pnt-First_Overlapp_pnt))		endif		Max_X=T_Max[i]		Start_X=T_Bkg1[i]		T_Bkg2[i]=End_Common		If(i==Last_Overlapp_pnt)			End_X=T_Bkg2[i]		else			End_X=T_Bkg1[i+1]		endif				If(i==First_Overlapp_pnt)			Variable Bkg_under_the_Max=Working_trace_copy(T_Bkg1[i])			Bkg_under_the_Max=Bkg_under_the_Max+(Working_trace_copy(T_Bkg2[i])-Working_trace_copy(T_Bkg1[i]))*(T_Max[i]-T_Bkg1[i])/(T_Bkg2[i]-T_Bkg1[i])			Peak_Imax[i]=Working_trace_copy(T_Max[i])-Bkg_under_the_Max		else			Extrapolation_name="Extrap_"+num2str(i)				//	extrap curve for the previous peak			Wave Extrap_prev=$Extrapolation_name			Peak_Imax[i]=Working_trace_copy(Max_X)-Extrap_prev(Max_X)		endif		Half_Height=Working_trace_copy(Max_X)-Peak_Imax[i]/2		FindLevel /Q/R=(Max_X,Start_X) Working_trace_copy, Half_Height		Peak_Half_H1[i]=min(V_LevelX,Max_X)		If(i!=First_Overlapp_pnt)			FindLevel /Q/R=(Max_X,Start_X) Extrap_prev, Half_Height			if(V_Flag==0)				Peak_Half_H1[i]=max(V_LevelX,Peak_Half_H1[i])			endif		endif		Calc_Rise_Fall(i)		Extrapolation_name="Extrap_"+num2str(i+1)		If(exists(Extrapolation_name)==1)			if (CheckName("Zoom_Win", 6)!=0)				RemoveFromGraph/Z/W=Zoom_Win $Extrapolation_name			endif			RemoveFromGraph/Z/W=Main_window $Extrapolation_name			KillWaves /Z $Extrapolation_name		endif		If(i!=Last_Overlapp_pnt)			Duplicate/O/R=(T_Bkg1[i+1],T_Bkg2[i]) Working_trace_copy qqq			SetScale/I x 0,(T_Bkg2[i]-T_Bkg1[i+1]),"s", qqq			A1=Working_trace_copy(T_Bkg1[i+1])-Working_trace_copy(T_Bkg2[i])			A0=Working_trace_copy(T_Bkg2[i])						String FitFunction=StringByKey("Fit", Fall_fit_Extrap[i])			If(strlen(FitFunction)==0)				FitFunction=Fall_Fit[i]				Fall_fit_Extrap[i] = ReplaceStringByKey("Fit", Fall_fit_Extrap[i], FitFunction)			endif						If((A1>0)&(cmpstr(FitFunction,"Line")!=0))				String ExpTau=StringByKey("Tau", Fall_fit_Extrap[i])				If(strlen(ExpTau)!=0)					tau1=str2num(ExpTau)				else					tau1=max(Fall_slope[i],Fall_slope2[i])				endif				Fall_fit_Extrap[i] = ReplaceStringByKey("Tau", Fall_fit_Extrap[i], num2str(tau1))				tau1=1/tau1*1000				qqq=A0+A1*exp(-x*tau1)				SetScale/I x T_Bkg1[i+1],T_Bkg2[i],"s", qqq				Duplicate/O qqq $Extrapolation_name			else				Slope=A1/(T_Bkg2[i]-T_Bkg1[i+1])				qqq=Working_trace_copy(T_Bkg1[i+1])-Slope*x				SetScale/I x T_Bkg1[i+1],T_Bkg2[i],"s", qqq				Duplicate/O qqq $Extrapolation_name//				If(cmpstr(StringByKey("Fit", Fall_fit_Extrap[i]),"")==0)					Fall_fit_Extrap[i] = ReplaceStringByKey("Fit", Fall_fit_Extrap[i], "Line")					Fall_fit_Extrap[i] = ReplaceStringByKey("Tau", Fall_fit_Extrap[i], num2str(-1))//				endif			endif			KillWaves /Z qqq		endif		FindLevel /Q/R=(Max_X, End_X) Working_trace_copy, Half_Height		If (V_flag==1)			If(i==Last_Overlapp_pnt)				Peak_Half_H2[i]=End_X			else				Extrapolation_name="Extrap_"+num2str(i+1)		//	extrap curve for the current peak				Wave Extrap_current=$Extrapolation_name				FindLevel /Q/R=(T_Bkg1[i+1], T_Bkg2[i]) Extrap_current, Half_Height				If (V_flag==0)					Peak_Half_H2[i]=V_LevelX				else					Peak_Half_H2[i]=End_X				endif			endif		else			Peak_Half_H2[i]=max(V_LevelX,Max_X)		endif		Peak_Base[i]=(T_Bkg2[i]-T_Bkg1[i])*1000		Peak_t05[i]=(Peak_Half_H2[i]-Peak_Half_H1[i])*1000		i+=1	while (i<=Last_Overlapp_pnt)	i=Last_Overlapp_pnt										//	Last peak pnt	Do		Area_total=area(Working_trace_copy,T_Bkg1[i],T_Bkg2[i])		If(i==First_Overlapp_pnt)			Area_bkg=(T_Bkg2[i]-T_Bkg1[i])*(Working_trace_copy(T_Bkg1[i])+Working_trace_copy(T_Bkg2[i]))/2			Peak_Q[i]=Area_total-Area_bkg-Area_Extra		else			Extrapolation_name="Extrap_"+num2str(i)			//	extrap curve for the previous peak			Wave Extrap_prev=$Extrapolation_name			Area_bkg=area(Extrap_prev,T_Bkg1[i],T_Bkg2[i])			Peak_Q[i]=Area_total-Area_bkg-Area_Extra			Area_Extra+=Peak_Q[i]		endif		Peak_Molec[i]=(Peak_Q[i])*3.121*10^6		i-=1	while (i>=First_Overlapp_pnt)EndFunction New_Peak_Position(New_T_Max)	Variable New_T_Max	SetDataFolder $"root:Quanta"	Wave T_Max=T_Max	NVAR Total_peaks_number=Total_peaks_number	If (Total_peaks_number==1)		If (T_Max[Total_peaks_number-1]==0)					//	no peaks exists yet			Change_waves("Make",0)			return 0		endif		endif	Variable Delta=CmpNum((T_Max[Total_peaks_number-1]),New_T_Max,18)	If (Delta==0)												//	peak already exists		If (Total_peaks_number==1)							// 	and an existing peak is the only one			return -1		else			return (-(Total_peaks_number-1))		endif	endif	If (Delta==-1)			return (Total_peaks_number)							//	new peak will be the last one	endif	Variable Peak_pnt=0	Do		Delta=CmpNum((T_Max[Peak_pnt]),New_T_Max,18)		If (Delta>=0)			If (Delta==0)										//	peak already exists				if (Peak_pnt==0)					return (-0.5)				else					return (-Peak_pnt)				endif			else				return (Peak_pnt)			endif		endif		Peak_pnt+=1	while(Total_peaks_number)EndFunction Calc_Peak_Parameters(Peak_pnt,Start_X,End_X)	Variable Peak_pnt	Variable Start_X	Variable End_X	Variable Max_X	SetDataFolder $"root:Quanta"	Wave T_Max=T_Max	Wave Peak_Imax=Peak_Imax	Wave Peak_t05=Peak_t05	Wave Peak_Q=Peak_Q	Wave Peak_Base=Peak_Base	Wave Peak_Half_H1=Peak_Half_H1	Wave Peak_Half_H2=Peak_Half_H2	Wave T_Bkg1=T_Bkg1	Wave T_Bkg2=T_Bkg2	Wave Working_trace_copy=Working_trace_copy	Wave Peak_Split1=Peak_Split1	Wave Peak_Split2=Peak_Split2	Wave/T Fall_fit_Extrap=Fall_fit_Extrap	T_Bkg1[Peak_pnt]=Start_X	T_Bkg2[Peak_pnt]=End_X	//	check for split overlapping spikes	If((Peak_Split1[Peak_pnt]!=0)%|(Peak_Split2[Peak_pnt]!=0))		Calc_split_peak_param(Peak_pnt)		return 0	endif	//	check for separated overlapping spikes	String First_and_Last=Check_for_Separated_peaks(Peak_pnt)	Variable First_separated_pnt=str2num(StringFromList(0,First_and_Last))	Variable Last_separated_pnt=str2num(StringFromList(1,First_and_Last))	If(First_separated_pnt!=Last_separated_pnt)		Variable i=First_separated_pnt		Do			Fall_fit_Extrap[i]=""			i+=1		while(i<=Last_separated_pnt)		Calc_Separated_peak_param(First_separated_pnt,Last_separated_pnt)		return 0	endif	//	find Imax, pA	Max_X=T_Max[Peak_pnt]	Variable Bkg_under_the_Max=Working_trace_copy(T_Bkg1[Peak_pnt])+(Working_trace_copy(T_Bkg2[Peak_pnt])-Working_trace_copy(T_Bkg1[Peak_pnt]))*(Max_X-T_Bkg1[Peak_pnt])/(T_Bkg2[Peak_pnt]-T_Bkg1[Peak_pnt])	Peak_Imax[Peak_pnt]=Working_trace_copy(Max_X)-Bkg_under_the_Max	//	find t(1/2), ms	Variable Half_Height=Working_trace_copy(Max_X) - (Working_trace_copy(Max_X)-Bkg_under_the_Max)/2	FindLevel /Q/R=(Max_X,Start_X) Working_trace_copy, Half_Height	Peak_Half_H1[Peak_pnt]=min(V_LevelX,Max_X)	FindLevel /Q/R=(Max_X, End_X) Working_trace_copy, Half_Height	If (V_flag==1)		Peak_Half_H2[Peak_pnt]=End_X	else		Peak_Half_H2[Peak_pnt]=max(V_LevelX,Max_X)	endif	Peak_t05[Peak_pnt]=(Peak_Half_H2[Peak_pnt]-Peak_Half_H1[Peak_pnt])*1000	Peak_Base[Peak_pnt]=(T_Bkg2[Peak_pnt]-T_Bkg1[Peak_pnt])*1000	//	find Q, pC, molecules	Calc_Peak_Q(Peak_pnt,T_Bkg1[Peak_pnt],T_Bkg2[Peak_pnt])	//	find rise and fall parameters	If ((Peak_Q[Peak_pnt]>0)%|(Peak_t05[Peak_pnt]>0))		Calc_Rise_Fall(Peak_pnt)	endif//	If ((Detection_limits(Peak_pnt)!=1))//		Calc_Rise_Fall(Peak_pnt)//	endifEndFunction Fall_fit_PopMenu(theTag,popNum,popStr) : PopupMenuControl	String theTag	Variable popNum	String popStr	SetDataFolder $"root:Quanta"	SVAR Fit_method=Fit_method	NVAR Peak_ID=Peak_ID//	Wave/T Fall_fit=Fall_fit	Variable Current_Peak=Peak_ID		If(cmpstr(popStr,Fit_method)!=0)		Fit_method=popStr		Change_Table ("q",0)		If(cmpstr(Fit_method,"DblExp")==0)			SetVariable Results_Fall_Chi, win=Options_Tab_Panels, disable=0		else			SetVariable Results_Fall_Chi, win=Options_Tab_Panels, disable=1		endif	endifEndFunction Fall_Extrap_PopMenu_Single(theTag,popNum,popStr) : PopupMenuControl	String theTag	Variable popNum	String popStr	SetDataFolder $"root:Quanta"	NVAR Peak_ID=Peak_ID	Wave/T Fall_fit_Extrap=Fall_fit_Extrap	Variable Peak_pnt=Peak_ID-1	String First_and_Last=Check_for_Separated_peaks(Peak_pnt)	Variable First_separated_pnt=str2num(StringFromList(0,First_and_Last))	Variable Last_separated_pnt=str2num(StringFromList(1,First_and_Last))	Fall_fit_Extrap[Peak_pnt] = ReplaceStringByKey("Fit", Fall_fit_Extrap[Peak_pnt], popStr)	Fall_fit_Extrap[Peak_pnt] = ReplaceStringByKey("Tau", Fall_fit_Extrap[Peak_pnt], "")	Calc_Separated_peak_param(First_separated_pnt,Last_separated_pnt)	Draw_lines_All(Peak_pnt)EndFunction Fall_fit_PopMenu_Single(theTag,popNum,popStr) : PopupMenuControl	String theTag	Variable popNum	String popStr	SetDataFolder $"root:Quanta"	NVAR Total_peaks_number=Total_peaks_number	NVAR Peak_ID=Peak_ID	Wave Fall_ChiRatio=Fall_ChiRatio	Wave Fall_time=Fall_time	Wave Fall_slope=Fall_slope	Wave Fall_slope2=Fall_slope2	Wave/T Fall_fit=Fall_fit	Wave/T Fall_fit_Extrap=Fall_fit_Extrap	Variable Peak_pnt=Peak_ID-1	String Fit_Function=Fall_fit[Peak_pnt]	If((cmpstr(popStr,Fit_Function)!=0)&(Total_peaks_number!=0))			Fall_fit[Peak_pnt]=popStr		String Slope_coeffs=Fit_Fall_Decay(Peak_pnt,popStr)		If(cmpstr(popStr,"DblExp")==0)			String Slope_coeffs_Exp=Fit_Fall_Decay(Peak_pnt,"Exp")			Variable Ratio=str2num(StringFromList(3,Slope_coeffs_Exp))/str2num(StringFromList(3,Slope_coeffs))			Fall_ChiRatio[Peak_pnt]=Ratio		else			Fall_ChiRatio[Peak_pnt]=1		endif		Fall_time[Peak_pnt]=str2num(StringFromList(0,Slope_coeffs))		Fall_slope[Peak_pnt]=str2num(StringFromList(1,Slope_coeffs))		Fall_slope2[Peak_pnt]=str2num(StringFromList(2,Slope_coeffs))		String First_and_Last=Check_for_Separated_peaks(Peak_pnt)		Variable First_separated_pnt=str2num(StringFromList(0,First_and_Last))		Variable Last_separated_pnt=str2num(StringFromList(1,First_and_Last))		If(First_separated_pnt!=Last_separated_pnt)			Fall_fit_Extrap[Peak_pnt] = ReplaceStringByKey("Fit", Fall_fit_Extrap[Peak_pnt], "")			Fall_fit_Extrap[Peak_pnt] = ReplaceStringByKey("Tau", Fall_fit_Extrap[Peak_pnt], "")			Calc_Separated_peak_param(First_separated_pnt,Last_separated_pnt)		endif		Draw_lines_All(Peak_pnt)	endifEndFunction Calc_Rise_Fall(Peak_pnt)	Variable Peak_pnt	SetDataFolder $"root:Quanta"	Wave T_Max=T_Max	Wave T_Bkg1=T_Bkg1	Wave T_Bkg2=T_Bkg2	Wave Peak_Split1=Peak_Split1	Wave Peak_Split2=Peak_Split2	Wave Peak_Half_H1=Peak_Half_H1	Wave Peak_Imax=Peak_Imax	Wave Rise_time=Rise_time	Wave Rise_Midpoint=Rise_Midpoint	Wave Rise_Lowpnt_X=Rise_Lowpnt_X	Wave Rise_Hipnt_X=Rise_Hipnt_X	Wave Rise_slope=Rise_slope	Wave Fall_time=Fall_time	Wave Fall_slope=Fall_slope	Wave Fall_slope2=Fall_slope2	Wave Fall_ChiRatio=Fall_ChiRatio	Wave Working_trace_copy=Working_trace_copy	Wave Foot_W=Foot_W	Wave Foot_end=Foot_end	Wave diff1_nosmooth=diff1_nosmooth	NVAR Rise_Low_Prc=Rise_Low_Prc	NVAR Rise_Hi_Prc=Rise_Hi_Prc	NVAR Total_peaks_number=Total_peaks_number	NVAR Fall_ChiRatio_Cutoff=Fall_ChiRatio_Cutoff	SVAR Fit_method=Fit_method	Variable Start_X=T_Bkg1[Peak_pnt]	Variable End_X=T_Bkg2[Peak_pnt]	Variable dI_max_Y,dI_max_X	Variable Half_Hight_L=(Working_trace_copy(T_Max[Peak_pnt])-Working_trace_copy(Start_X))/2	FindLevel/Q/R=(T_Max[Peak_pnt],Start_X) Working_trace_copy, (Working_trace_copy(Start_X)+Half_Hight_L )	Variable Half_Hight_L_X=V_LevelX	//	Check for overlaps	If ((T_Max[Peak_pnt-1]!=T_Max[Peak_pnt])&(T_Max[Peak_pnt-1])>(T_Bkg1[Peak_pnt]))		Start_X=T_Bkg2[Peak_pnt-1]	endif	If ((T_Max[Peak_pnt+1]!=T_Max[Peak_pnt])&(T_Max[Peak_pnt+1])<(T_Bkg2[Peak_pnt]))		End_X=T_Bkg1[Peak_pnt+1]	endif	If(Peak_Split1[Peak_pnt])		Start_X=Peak_Split1[Peak_pnt]	endif	If(Peak_Split2[Peak_pnt])		End_X=Peak_Split2[Peak_pnt]	endif	If(Rise_Midpoint[Peak_pnt]==0)		If((pnt2x(diff1_nosmooth,0)>Start_X)%|(pnt2x(diff1_nosmooth,numpnts(diff1_nosmooth))<End_X))			Duplicate/O Working_trace_copy, diff1_nosmooth			Differentiate diff1_nosmooth			NVAR Smoothing_Factor_diff1=Smoothing_Factor_diff1			Variable Binomial_coeff=Gaussian_to_Binomial_Calc(Smoothing_Factor_diff1)			Smooth/E=1 Binomial_coeff, diff1_nosmooth		endif		wavestats/Q/R=(T_Max[Peak_pnt],Half_Hight_L_X), diff1_nosmooth		Rise_Midpoint[Peak_pnt]=V_maxloc		dI_max_X=V_maxloc		dI_max_Y=max(Working_trace_copy(Start_X)+Half_Hight_L, Working_trace_copy(dI_max_X))	else		dI_max_X=Rise_Midpoint[Peak_pnt]		dI_max_Y=Working_trace_copy(Rise_Midpoint[Peak_pnt])	endif	//	calculate the upper and lower points for the linear fit	Variable Rise_Delta=(Working_trace_copy(T_Max[Peak_pnt])-dI_max_Y)/2	Variable Three_fouth_Height_L_Y=dI_max_Y+Rise_Delta	Variable One_fouth_Height_L_Y=dI_max_Y-Rise_Delta	Variable One_fouth_Height_L_X, Three_fouth_Height_L_X	FindLevel/P/Q/R=(T_Max[Peak_pnt],Start_X) Working_trace_copy, Three_fouth_Height_L_Y	Three_fouth_Height_L_X=pnt2x(Working_trace_copy, V_LevelX )	Rise_Hipnt_X[Peak_pnt]=Three_fouth_Height_L_X	FindLevel/P/Q/R=(T_Max[Peak_pnt],Start_X) Working_trace_copy, One_fouth_Height_L_Y	One_fouth_Height_L_X=pnt2x(Working_trace_copy, V_LevelX )	Rise_Lowpnt_X[Peak_pnt]=One_fouth_Height_L_X	//	find Rise Slope	If(Rise_Lowpnt_X[Peak_pnt]==Rise_Hipnt_X[Peak_pnt])		return 0	else		Variable Rise_B_coeff=Fit_Rise(Rise_Lowpnt_X[Peak_pnt],Rise_Hipnt_X[Peak_pnt])	endif	Rise_slope[Peak_pnt]=Rise_B_coeff/1000	//	find Foot end at the extrapolation of the linear fit to spike baseline	Foot_End[Peak_pnt]=Extrapolate_to_Baseline(Peak_pnt)	//	find Risetime	Variable Rise_Segment=(Rise_Hi_Prc-Rise_Low_Prc)/100	Rise_time[Peak_pnt]=(T_max[Peak_pnt]-Foot_end[Peak_pnt])*1000*Rise_Segment	//	find foot width, hight and charge 	Calc_Foot_Parameters(Peak_pnt)	//	recalculate rising phase parameters for spikes without the feet	if(Foot_W[Peak_pnt]==0)		Rise_Midpoint[Peak_pnt]=Peak_Half_H1[Peak_pnt]		Variable Hight_L=Working_trace_copy(T_Max[Peak_pnt])-Working_trace_copy(T_Bkg1[Peak_pnt])		Variable Upper_Y=Working_trace_copy(T_Bkg1[Peak_pnt])+Hight_L*Rise_Hi_Prc/100		Variable Lower_Y=Working_trace_copy(T_Bkg1[Peak_pnt])+Hight_L*Rise_Low_Prc/100		FindLevel/P/Q/R=(T_Max[Peak_pnt],T_Bkg1[Peak_pnt]) Working_trace_copy, Upper_Y		Rise_Hipnt_X[Peak_pnt]=pnt2x(Working_trace_copy, V_LevelX )		FindLevel/P/Q/R=(T_Max[Peak_pnt],T_Bkg1[Peak_pnt]) Working_trace_copy, Lower_Y		Rise_Lowpnt_X[Peak_pnt]=pnt2x(Working_trace_copy, V_LevelX )		Rise_B_coeff=Fit_Rise(Rise_Lowpnt_X[Peak_pnt],Rise_Hipnt_X[Peak_pnt])		Rise_slope[Peak_pnt]=Rise_B_coeff/1000		Rise_time[Peak_pnt]=(T_max[Peak_pnt]-Foot_end[Peak_pnt])*1000*Rise_Segment	endif	//	find falling phase parameters	Wave/t Fall_fit=Fall_fit	Variable Change_Fit=0	If(cmpstr(Fall_fit[Peak_pnt],"")==0)		Fall_fit[Peak_pnt]=Fit_method		Change_Fit=1	endif	String Fit_Function=Fall_fit[Peak_pnt]	String Slope_coeffs=Fit_Fall_Decay(Peak_pnt,Fit_Function)		If(cmpstr(Fit_method,"DblExp")==0)		String Slope_coeffs_Exp=Fit_Fall_Decay(Peak_pnt,"Exp")		Variable Ratio=str2num(StringFromList(3,Slope_coeffs_Exp))/str2num(StringFromList(3,Slope_coeffs))		If(Ratio<Fall_ChiRatio_Cutoff)			if(Change_Fit==1)				Slope_coeffs=Slope_coeffs_Exp				Fall_fit[Peak_pnt]="Exp"			endif		endif				Fall_ChiRatio[Peak_pnt]=Ratio	else		Fall_ChiRatio[Peak_pnt]=1	endif	Fall_time[Peak_pnt]=str2num(StringFromList(0,Slope_coeffs))	Fall_slope[Peak_pnt]=str2num(StringFromList(1,Slope_coeffs))	Fall_slope2[Peak_pnt]=str2num(StringFromList(2,Slope_coeffs))EndFunction Fit_Rise(Start_X,End_X)	Variable Start_X	Variable End_X	SetDataFolder $"root:Quanta"	Make/O/N=0 W_coef		If(x2pnt(Working_trace_copy,End_X)-x2pnt(Working_trace_copy,Start_X)<2)		return 0	endif	Duplicate/O/R=(Start_X,End_X) Working_trace_copy, Rise_phase,qqq	SetScale/I x 0,(End_X-Start_X),"s", Rise_phase	CurveFit/Q/N line Rise_phase  /D=Rise_phase 	SetScale/I x Start_X,End_X,"s", Rise_phase	KillWaves /Z qqq	return W_coef[1]EndFunction Extrapolate_to_Baseline(Peak_pnt)	Variable Peak_pnt	Wave T_Bkg1=T_Bkg1	Wave Rise_Midpoint=Rise_Midpoint	Wave Rise_slope=Rise_slope	Wave Working_trace_copy=Working_trace_copy	Variable Midpoint_Y=Working_trace_copy(Rise_Midpoint[Peak_pnt])	Variable T_Bkg1_Y=Working_trace_copy(T_Bkg1[Peak_pnt])	Variable Rise_B_Coeff=Rise_slope[Peak_pnt]*1000		Variable Foot_X=(1/Rise_B_Coeff)*(Midpoint_Y-T_Bkg1_Y)	Foot_X=(Rise_Midpoint[Peak_pnt]-Foot_X)	Return Foot_XEndFunction New_Rise(ctrlName) : ButtonControl	String ctrlName	SetDataFolder $"root:Quanta"	NVAR Peak_ID=Peak_ID	Wave T_Max=T_Max	Wave T_Bkg1=T_Bkg1	Wave Foot_End=Foot_End	Wave Rise_Lowpnt_X=Rise_Lowpnt_X	Wave Rise_Hipnt_X=Rise_Hipnt_X	Wave Rise_time=Rise_time	Wave Rise_Midpoint=Rise_Midpoint	Wave Rise_slope=Rise_slope	Wave Working_trace_copy=Working_trace_copy	NVAR Rise_Low_Prc=Rise_Low_Prc	NVAR Rise_Hi_Prc=Rise_Hi_Prc	Variable Peak_pnt=Peak_ID-1	Make/O/N=0 W_coef	Variable Start_X, 	End_X	If (xcsr(A)<xcsr(B))		Start_X=xcsr(A)		End_X=xcsr(B)	else		Start_X=xcsr(B)		End_X=xcsr(A)	endif	If ((Start_X>=T_Max[Peak_pnt])%|(End_X<=T_Bkg1[Peak_pnt]))		abort "The coursors have to be between spike's Start and Maximum!"	endif		Variable Rise_B_coeff=Fit_Rise(Start_X,End_X)	Rise_slope[Peak_pnt]=Rise_B_coeff/1000	Rise_Lowpnt_X[Peak_pnt]=Start_X	Rise_Hipnt_X[Peak_pnt]=End_X	Rise_Midpoint[Peak_pnt]=pnt2x(Rise_phase, round(numpnts(Rise_phase)/2))	Foot_End[Peak_pnt]=Extrapolate_to_Baseline(Peak_pnt)	Variable Rise_Segment=(Rise_Hi_Prc-Rise_Low_Prc)/100	Rise_time[Peak_pnt]=(T_max[Peak_pnt]-Foot_end[Peak_pnt])*1000*Rise_Segment	Calc_Foot_Parameters(Peak_pnt)	Draw_lines_All(Peak_pnt)EndFunction/S Fit_Fall_Decay(Peak_pnt,Fit_Function)	Variable Peak_pnt	String Fit_Function	SetDataFolder $"root:Quanta"	Wave T_Max=T_Max	Wave T_Bkg1=T_Bkg1	Wave T_Bkg2=T_Bkg2	Wave Peak_Split1=Peak_Split1	Wave Peak_Split2=Peak_Split2	Wave Fall_time=Fall_time	Wave Fall_slope=Fall_slope	Wave Fall_slope2=Fall_slope2	Wave Working_trace_copy=Working_trace_copy	NVAR Total_peaks_number=Total_peaks_number	NVAR Fall_ChiRatio_Cutoff=Fall_ChiRatio_Cutoff	Make/O/N=0 W_coef		Variable End_X=T_Bkg2[Peak_pnt]	If ((T_Max[Peak_pnt+1]!=T_Max[Peak_pnt])&(T_Max[Peak_pnt+1])<(T_Bkg2[Peak_pnt]))		End_X=T_Bkg1[Peak_pnt+1]	endif	If(Peak_Split2[Peak_pnt])		End_X=Peak_Split2[Peak_pnt]	endif	Variable Hight_R=Working_trace_copy(T_Max[Peak_pnt])-Working_trace_copy(End_X)	Variable One_fouth_Height_Y_R=Working_trace_copy(End_X)+Hight_R*0.25	Variable Three_fouth_Height_Y_R=Working_trace_copy(End_X)+Hight_R*0.75	Variable One_fouth_Height_X_R, Three_fouth_Height_X_R	FindLevel /Q/R=(T_Max[Peak_pnt],End_X) Working_trace_copy, Three_fouth_Height_Y_R	Three_fouth_Height_X_R=V_LevelX	FindLevel /Q/R=(T_Max[Peak_pnt],End_X) Working_trace_copy, One_fouth_Height_Y_R	One_fouth_Height_X_R=V_LevelX	Variable Fall_DeltaT=(One_fouth_Height_X_R-Three_fouth_Height_X_R)*1000	Variable Fall_Slope_coeff, Fall_Slope_coeff2=0,Fall_Chi=1	If(x2pnt(Working_trace_copy,End_X)-x2pnt(Working_trace_copy,Three_fouth_Height_X_R)<5)		return "0,0,0,0"	endif	If(cmpstr(Fit_Function,"DblExp")==0)		PauseUpdate		Duplicate/O/R=(Three_fouth_Height_X_R,End_X) Working_trace_copy, Fall_phase		SetScale/I x 0,(End_X-Three_fouth_Height_X_R),"s", Fall_phase		CurveFit/Q/N dblexp Fall_phase /D=Fall_phase 		Fall_Chi=V_chisq		Fall_Slope_coeff=min((1/(W_coef[4]/1000)),(1/(W_coef[2]/1000)))		Fall_Slope_coeff2=max((1/(W_coef[4]/1000)),(1/(W_coef[2]/1000)))		SetScale/I x Three_fouth_Height_X_R,End_X,"s", Fall_phase	else		If(cmpstr(Fit_Function,"Exp")==0)			PauseUpdate			Duplicate/O/R=(Three_fouth_Height_X_R,End_X) Working_trace_copy, Fall_phase			SetScale/I x 0,(End_X-Three_fouth_Height_X_R),"s", Fall_phase			CurveFit/Q/N exp Fall_phase /D=Fall_phase 			Fall_Chi=V_chisq			SetScale/I x Three_fouth_Height_X_R,End_X,"s", Fall_phase			Fall_Slope_coeff=1/(W_coef[2]/1000)		else			PauseUpdate			Duplicate/O/R=(Three_fouth_Height_X_R,One_fouth_Height_X_R) Working_trace_copy, Fall_phase			CurveFit/Q/N line Fall_phase  /D=Fall_phase 			Fall_Slope_coeff=-W_coef[1]/1000		endif	endif	String Formated_String, Slope_coeffs	sprintf Formated_String, "%.12f", Fall_DeltaT	Slope_coeffs=Formated_String+";"	sprintf Formated_String, "%.12f", Fall_Slope_coeff	Slope_coeffs=Slope_coeffs+Formated_String+";"	sprintf Formated_String, "%.12f", Fall_Slope_coeff2	Slope_coeffs=Slope_coeffs+Formated_String+";"	sprintf Formated_String, "%.18f", Fall_Chi	Slope_coeffs=Slope_coeffs+Formated_String+";"	return Slope_coeffsEndFunction Calc_Peak_Q(Peak_pnt,Start_X,End_X)	Variable Peak_pnt	Variable Start_X	Variable End_X	SetDataFolder $"root:Quanta"	Wave Peak_Q=Peak_Q	Wave Peak_Molec=Peak_Molec	Wave Working_trace_copy=Working_trace_copy	Variable Area_total=area(Working_trace_copy,Start_X,End_X)	Variable Area_bkg=(End_X-Start_X)*(Working_trace_copy(Start_X)+Working_trace_copy(End_X))/2	Peak_Q[Peak_pnt]=Area_total-Area_bkg	Peak_Molec[Peak_pnt]=(Peak_Q[Peak_pnt])*3.121*10^6EndFunction Calc_Foot_Parameters(Peak_pnt) : ButtonControl	Variable Peak_pnt	SetDataFolder $"root:Quanta"	Wave T_Max=T_Max	Wave T_Bkg1=T_Bkg1	Wave T_Bkg2=T_Bkg2	Wave Foot_Q=Foot_Q	Wave Foot_Molec=Foot_Molec	Wave Foot_W=Foot_W	Wave Foot_end=Foot_end	Wave Rise_Lowpnt_X=Rise_Lowpnt_X	Wave Foot_I= Foot_I	Wave Peak_Split1=Peak_Split1	Wave Rise_time=Rise_time	Wave Working_trace_copy=Working_trace_copy	NVAR Bkg_noise_I=Bkg_noise_I	NVAR Detection_Foot_Mult=Detection_Foot_Mult	NVAR SSFoot_Do=SSFoot_Do	NVAR Native_Foot_Del=Native_Foot_Del	NVAR Foot_Min_W=Foot_Min_W	NVAR Rise_Hi_Prc=Rise_Hi_Prc	NVAR Rise_Low_Prc=Rise_Low_Prc		Variable Start_X=T_Bkg1[Peak_pnt]	Variable Foot_End_X_low=Foot_end[Peak_pnt]	Variable Foot_End_X_hi=Rise_Lowpnt_X[Peak_pnt]	If((Peak_Split1(Peak_pnt)!=0)%|(Foot_End_X_low<=Start_X))		Foot_Q[Peak_pnt]=0		Foot_Molec[Peak_pnt]=0		Foot_W[Peak_pnt]=0		Foot_I[Peak_pnt]=0		return 0	endif	//	calculate foot duration	FindLevel/Q/R=(Start_X,T_Max[Peak_pnt]) Working_trace_copy, (Working_trace_copy(Start_X)+Detection_Foot_Mult*Bkg_noise_I)	If((Foot_End_X_low-V_LevelX)>0)		Foot_W[Peak_pnt]=(Foot_End_X_low-Start_X)*1000	else		Foot_W[Peak_pnt]=0	endif	//	delete 'native' PSF		If((Native_Foot_Del==1)&(Foot_W[Peak_pnt]>Foot_Min_W))		Variable Native_foot_W=Rise_time[Peak_pnt]*13.2/(Rise_Hi_Prc-Rise_Low_Prc)		If (Foot_W[Peak_pnt]<=Native_foot_W)			Foot_W[Peak_pnt]=0		endif	endif	//	calculate foot current	If((SSFoot_Do==1)&(Foot_W[Peak_pnt]>Foot_Min_W))		//	find PSF with steady states		Variable SteadyState=Find_SteadyState(Foot_End_X_low, Start_X, Foot_Min_W/1000)		If(SteadyState)			Foot_I[Peak_pnt]=SteadyState-Working_trace_copy(Start_X)		endif	else		Foot_I[Peak_pnt]=mean(Working_trace_copy, Start_X, Foot_End_X_low)-Working_trace_copy(Start_X)	endif	//	calculate foot charge	If(Foot_W[Peak_pnt]>0)		Variable Area_total=area(Working_trace_copy,Start_X,Foot_End_X_hi)		Variable Ft_Bkg2_Y=Y_offset(Start_X, T_Bkg2[Peak_pnt], Foot_End_X_hi)		Variable Foot_W_all=Foot_End_X_hi-Start_X		Variable Area_bkg=Foot_W_all*(Working_trace_copy(Start_X)+Ft_Bkg2_Y)/2		Area_total=Area_total-Area_bkg		Variable Tr_Area=(Foot_End_X_hi-Foot_End_X_low)*(Working_trace_copy(Foot_End_X_hi)-Ft_Bkg2_Y)/2		Foot_Q[Peak_pnt]=Area_total-Tr_Area		Foot_Molec[Peak_pnt]=(Foot_Q[Peak_pnt])*3.121*10^6	endif	//	check the validity of PSF values	If(Detection_limits_Foot(Peak_pnt))		Foot_W[Peak_pnt]=0		Foot_I[Peak_pnt]=0		Foot_Q[Peak_pnt]=0		Foot_Molec[Peak_pnt]=0	endifEnd//	All in secondsFunction Find_SteadyState (Start_X, End_X, Itteration)	Variable Start_X	Variable End_X	Variable Itteration	Wave Working_trace_copy=Working_trace_copy	NVAR Bkg_noise_I=Bkg_noise_I	Variable Delta=Start_X-End_X	If(Delta<0)	//	The search is toward trace's end		Itteration=0-Itteration	endif	If(abs(Delta)<abs(Itteration))		return 0	endif	Variable Step_back_1=Start_X	Variable Steady_State1=mean(Working_trace_copy, Step_back_1, Step_back_1-Itteration/2)	Variable Steady_State2=mean(Working_trace_copy, Step_back_1-Itteration/2, Step_back_1-Itteration)	Variable Upper_lim=Steady_State1+Bkg_noise_I	Variable Lower_lim=Steady_State1-Bkg_noise_I	Variable Steady_State	do		If((Steady_State2<Upper_lim)&(Steady_State2>Lower_lim))			Steady_State=mean(Working_trace_copy, Step_back_1, Step_back_1-Itteration)			return Steady_State			break		endif		Step_back_1-=Itteration/2		Steady_State1=mean(Working_trace_copy, Step_back_1, Step_back_1-Itteration/2)		Steady_State2=mean(Working_trace_copy, Step_back_1-Itteration/2, Step_back_1-Itteration)		Upper_lim=Steady_State1+Bkg_noise_I		Lower_lim=Steady_State1-Bkg_noise_I	while(abs(Step_back_1-End_X)>abs(Itteration))EndFunction New_Foot_I(ctrlName) : ButtonControl	String ctrlName	SetDataFolder $"root:Quanta"	NVAR Peak_ID=Peak_ID	Wave T_Bkg1=T_Bkg1	Wave Foot_I=Foot_I	Wave Working_trace_copy=Working_trace_copy	Variable Peak_pnt=Peak_ID-1	Foot_I[Peak_pnt]=Working_trace_copy(xcsr(A))-Working_trace_copy(T_Bkg1[Peak_pnt])	Draw_lines_All(Peak_pnt)EndFunction Delete_Foot(ctrlName) : ButtonControl	String ctrlName	SetDataFolder $"root:Quanta"	NVAR Peak_ID=Peak_ID	Wave T_Max=T_Max	Wave T_Bkg1=T_Bkg1	Wave Peak_Half_H1=Peak_Half_H1	Wave Foot_Q=Foot_Q	Wave Foot_Molec=Foot_Molec	Wave Foot_W=Foot_W	Wave Foot_end=Foot_end	Wave Rise_Lowpnt_X=Rise_Lowpnt_X	Wave Rise_Hipnt_X=Rise_Hipnt_X	Wave Foot_I=Foot_I	Wave Rise_Midpoint=Rise_Midpoint	Wave Rise_slope=Rise_slope	Wave Rise_time=Rise_time	Wave Working_Trace_Copy=Working_Trace_Copy	NVAR Rise_Hi_Prc=Rise_Hi_Prc	NVAR Rise_Low_Prc=Rise_Low_Prc	Variable Peak_pnt=Peak_ID-1	Rise_Midpoint[Peak_pnt]=Peak_Half_H1[Peak_pnt]	Variable Hight_L=Working_trace_copy(T_Max[Peak_pnt])-Working_trace_copy(T_Bkg1[Peak_pnt])	Variable Upper_Y=Working_trace_copy(T_Bkg1[Peak_pnt])+Hight_L*Rise_Hi_Prc/100	Variable Lower_Y=Working_trace_copy(T_Bkg1[Peak_pnt])+Hight_L*Rise_Low_Prc/100	FindLevel/P/Q/R=(T_Max[Peak_pnt],T_Bkg1[Peak_pnt]) Working_trace_copy, Upper_Y	Rise_Hipnt_X[Peak_pnt]=pnt2x(Working_trace_copy, V_LevelX )	FindLevel/P/Q/R=(T_Max[Peak_pnt],T_Bkg1[Peak_pnt]) Working_trace_copy, Lower_Y	Rise_Lowpnt_X[Peak_pnt]=pnt2x(Working_trace_copy, V_LevelX )	Variable Rise_B_coeff=Fit_Rise(Rise_Lowpnt_X[Peak_pnt],Rise_Hipnt_X[Peak_pnt])	Rise_slope[Peak_pnt]=Rise_B_coeff/1000	Foot_End[Peak_pnt]=Extrapolate_to_Baseline(Peak_pnt)	Variable Rise_Segment=(Rise_Hi_Prc-Rise_Low_Prc)/100	Rise_time[Peak_pnt]=(T_max[Peak_pnt]-Foot_end[Peak_pnt])*1000*Rise_Segment	Foot_Q[Peak_pnt]=0	Foot_W[Peak_pnt]=0	Foot_Molec[Peak_pnt]=0	Foot_I[Peak_pnt]=0		Draw_lines_All(Peak_pnt)EndFunction New_Baseline(ctrlName) : ButtonControl	String ctrlName	SetDataFolder $"root:Quanta"	NVAR Peak_ID=Peak_ID	NVAR Total_peaks_number=Total_peaks_number	Wave T_Max=T_Max	Wave T_Bkg1=T_Bkg1	Wave T_Bkg2=T_Bkg2	Wave Peak_Split1=Peak_Split1	Wave Peak_Split2=Peak_Split2	Wave Rise_time=Rise_time	Wave Rise_Midpoint=Rise_Midpoint	Wave Rise_Lowpnt_X=Rise_Lowpnt_X	Wave Rise_Hipnt_X=Rise_Hipnt_X	Wave Rise_slope=Rise_slope	Wave Foot_end=Foot_end	Wave/T Fall_fit=Fall_fit	Wave Working_Trace_Copy=Working_Trace_Copy	Variable Peak_pnt=Peak_ID-1	If(Total_peaks_number==0)		abort	endif	Variable Start_X=min(xcsr(A),xcsr(B))	Variable End_X=max(xcsr(A),xcsr(B))		If((T_Bkg1[Peak_pnt]>T_Bkg2[Peak_pnt-1])%|(Peak_pnt==0))		FindLevel /Q/R=(T_Max[Peak_pnt],Start_X ) Working_Trace_Copy, Working_Trace_Copy(Start_X)		If(V_flag==0)			Start_X=V_LevelX		endif	endif		If ((Start_X>=T_Max[Peak_pnt])%|(End_X<=T_Max[Peak_pnt]))		abort "Spike Maximum has to be between the coursors!"	endif	If ((Peak_Split1[Peak_pnt]!=0)%|(Peak_Split2[Peak_pnt]!=0))		T_Bkg1[Peak_pnt]=Start_X		T_Bkg2[Peak_pnt]=End_X		Calc_split_peak_param(Peak_pnt)	else		Fall_fit[Peak_pnt]=""		Rise_Lowpnt_X[Peak_pnt]=0		Rise_Hipnt_X[Peak_pnt]=0		Foot_end[Peak_pnt]=0		Rise_slope[Peak_pnt]=0		Rise_Midpoint[Peak_pnt]=0		Calc_Peak_Parameters(peak_pnt,Start_X,End_X)	endif			Draw_lines_All(Peak_pnt)EndFunction New_T_Max(ctrlName) : ButtonControl	String ctrlName	SetDataFolder $"root:Quanta"	NVAR Peak_ID=Peak_ID	NVAR Total_peaks_number=Total_peaks_number	Variable Peak_pnt=Peak_ID-1	Wave T_Max=T_Max	Wave Peak_Split1=Peak_Split1	Wave Peak_Split2=Peak_Split2	Wave T_Bkg1=T_Bkg1	Wave T_Bkg2=T_Bkg2	Wave Rise_Midpoint=Rise_Midpoint	Wave Rise_Lowpnt_X=Rise_Lowpnt_X	Wave Rise_Hipnt_X=Rise_Hipnt_X	Wave Rise_slope=Rise_slope	Wave Foot_End=Foot_End	Wave/T Fall_fit=Fall_fit	If(Total_peaks_number==0)		abort	endif	If ((xcsr(A)<=T_Bkg1[Peak_pnt])%|(xcsr(A)>=T_Bkg2[Peak_pnt]))		abort "C'mon, the Maximum has to be between the Start and the End on a spike!"	endif	T_Max[Peak_pnt]=xcsr(A)	Fall_fit[Peak_pnt]=""	Rise_Lowpnt_X[Peak_pnt]=0	Rise_Hipnt_X[Peak_pnt]=0	Rise_slope[Peak_pnt]=0	Rise_Midpoint[Peak_pnt]=0	Foot_end[Peak_pnt]=0	Calc_Peak_Parameters(Peak_pnt,T_Bkg1[Peak_pnt],T_Bkg2[Peak_pnt])	Draw_lines_All(Peak_pnt)EndFunction Delete_Peak(ctrlName) : ButtonControl	String ctrlName	SetDataFolder $"root:Quanta"	NVAR Peak_ID=Peak_ID	NVAR Total_peaks_number=Total_peaks_number	Variable Peak_pnt=Peak_ID-1	Wave Peak_Split1=Peak_Split1	Wave Peak_Split2=Peak_Split2	Wave Peak_Num=Peak_Num	Wave T_Bkg1=T_Bkg1	Wave T_Bkg2=T_Bkg2	Wave T_Max=T_Max	Wave/T Fall_fit_Extrap=Fall_fit_Extrap	If(Total_peaks_number==0)		abort	endif		Wavestats/Q Peak_Num	If( V_npnts==1)		De_novo()	else		Variable Over=0		String First_and_Last=Check_for_Separated_peaks(Peak_pnt)		Variable First_separated_pnt=str2num(StringFromList(0,First_and_Last))		Variable Last_separated_pnt=str2num(StringFromList(1,First_and_Last))		If(First_separated_pnt!=Last_separated_pnt)			Variable i=First_separated_pnt			Do				Fall_fit_Extrap[i]=""				i+=1			while(i<=Last_separated_pnt)			Over=1		endif		String TagName="Max"+num2str(Total_peaks_number-1)		Tag/K/N=$TagName				If (Peak_Split1[Peak_pnt])			Peak_Split2[Peak_pnt-1]=Peak_Split2[Peak_pnt]			Over=1		endif		If ((Peak_Split1[Peak_pnt]==0)&(Peak_Split2[Peak_pnt]!=0))			Peak_Split1[Peak_pnt+1]=0			Over=1		endif				Change_waves("Delete",Peak_pnt)		Wavestats/Q Peak_Num		Total_peaks_number=V_npnts		If(Over==1)			Calc_Peak_Parameters(Peak_pnt,T_Bkg1[Peak_pnt],T_Bkg2[Peak_pnt])			Calc_Peak_Parameters(Peak_pnt-1,T_Bkg1[Peak_pnt-1],T_Bkg2[Peak_pnt-1])		endif	endif	If (Peak_ID>=Total_peaks_number)		Peak_ID=Total_peaks_number	else		Peak_Num[Peak_pnt,]-=1	endif	SetVariable ID,limits={1,(Total_peaks_number),1},win=Main_window	if (CheckName("Zoom_Win", 6)!=0)		SetVariable ID,limits={1,(Total_peaks_number),1},win=Zoom_Win	endif	if(Total_peaks_number)		Draw_lines_All(Peak_ID-1)	endifEndFunction Delete_All_Peaks(ctrlName) : ButtonControl	String ctrlName	SetDataFolder $"root:Quanta"	NVAR Total_peaks_number=Total_peaks_number	If(Total_peaks_number==0)		abort	endif	DoAlert 1, "Do you really want to kill them all!"	If (V_Flag==1)		De_novo()	else		abort	endif		End///___________________________________//____________Displaying the data________//___________________________________Function Peak_surf(ctrlName) : ButtonControl	String ctrlName	SetDataFolder $"root:Quanta"	NVAR Peak_ID=Peak_ID	NVAR Total_peaks_number=Total_peaks_number	If(Total_peaks_number>0)		If(cmpstr(ctrlName, "Next")==0)			Peak_ID+=1			If (Peak_ID>Total_peaks_number)				Peak_ID=Total_peaks_number			endif		else			Peak_ID-=1			If (Peak_ID<1)				Peak_ID=1			endif		endif	else		abort	endif	Peak_locator(Peak_ID-1)EndFunction Peak_locator_ID(ctrlName,varNum,varStr,varName) : SetVariableControl	String ctrlName	Variable varNum	String varStr	String varName	SetDataFolder $"root:Quanta"	NVAR Peak_ID=Peak_ID	Peak_locator(Peak_ID-1)EndFunction Peak_locator(Peak_pnt)	Variable Peak_pnt	SetDataFolder $"root:Quanta"	SVAR Zoom_On=Zoom_On	Wave Peak_Half_H1=Peak_Half_H1	Wave T_Max=T_Max	Wave Peak_Imax=Peak_Imax	Wave Peak_Half_H2=Peak_Half_H2	Wave T_Bkg1=T_Bkg1	Wave T_Bkg2=T_Bkg2	Wave Working_trace_copy=Working_trace_copy	Variable Max_location=T_Max[Peak_pnt]	NVAR Total_peaks_number=Total_peaks_number	If(Total_peaks_number==0)		abort	endif	Variable Bkg1_location=T_Bkg1[Peak_pnt]	Variable Tail_location=T_Bkg2[Peak_pnt]	If ((T_Max[Peak_pnt])<(T_Bkg2[Peak_pnt-1]))		Bkg1_location=T_Bkg1[Peak_pnt-1]	endif	If ((T_Max[Peak_pnt])>(T_Bkg1[Peak_pnt+1]))		Tail_location=T_Bkg2[Peak_pnt+1]	endif	Variable Bottom_Y=min(Working_trace_copy(Bkg1_location),Working_trace_copy(Tail_location))	wavestats/Q/R=(Bkg1_location,Tail_location) Working_trace_copy	Variable Highest_point_X=V_maxloc	Variable Highest_point_Y=Working_trace_copy(Highest_point_X)-Bottom_Y		If(cmpstr(Zoom_On,"On")==0)		SetAxis left (Bottom_Y-0.2*Highest_point_Y),(Working_trace_copy(Highest_point_X)+0.2*Highest_point_Y)		SetAxis bottom (Bkg1_location-1*(Tail_location-Highest_point_X)),(Tail_location+1*(Tail_location-Highest_point_X))	endif	Cursor/W=Main_window A Working_trace_copy T_Bkg1[Peak_pnt]	Cursor/W=Main_window B Working_trace_copy T_Bkg2[Peak_pnt]	Draw_lines_All(Peak_pnt)	Slider_Reset("qqq")EndFunction Generate_annotation(Current_Peak)	Variable Current_Peak	SetDataFolder $"root:Quanta"	Wave T_Max=T_Max	Wave Peak_Base=Peak_Base	Wave Peak_t05=Peak_t05	Wave Peak_Imax=Peak_Imax	Wave Peak_Q=Peak_Q	Wave Peak_Molec=Peak_Molec	Wave Rise_time=Rise_time	Wave Rise_slope=Rise_slope	Wave/T Fall_fit=Fall_fit	Wave Fall_time=Fall_time	Wave Fall_slope=Fall_slope	Wave Fall_slope2=Fall_slope2	Wave Foot_I=Foot_I	Wave Foot_W=Foot_W	Wave Foot_Q=Foot_Q	Wave Foot_Molec=Foot_Molec	NVAR Rise_Low_Prc=Rise_Low_Prc	NVAR Rise_Hi_Prc=Rise_Hi_Prc	NVAR Total_peaks_number=Total_peaks_number	SVAR Values_to_show=Values_to_show	String Peak_Parameters_list="",Peak_Parameters_One	String One_wave	If(Total_peaks_number==0)		return 0	endif	Variable i=1	do		One_wave=StringFromList(i, Values_to_show ,",")		if(strlen(One_wave) == 0 )			break		endif		If(cmpstr(One_wave,"T_Max")==0)			sprintf Peak_Parameters_One, "\Z09\F'Arial'\K(0,0,0) Time= %.2W1Ps\K(0,0,52224)", T_Max[Current_Peak]			Peak_Parameters_list+=Peak_Parameters_One		endif		If(cmpstr(One_wave,"Peak_Base")==0)			sprintf Peak_Parameters_One, "\Z09\F'Arial'\rBase= %.1W1Ps", Peak_Base[Current_Peak]/1000			Peak_Parameters_list+=Peak_Parameters_One		endif		If(cmpstr(One_wave,"Peak_t05")==0)			sprintf Peak_Parameters_One, "\Z09\F'Arial'\rt1/2= %.1W1Ps", Peak_t05[Current_Peak]/1000			Peak_Parameters_list+=Peak_Parameters_One		endif		If(cmpstr(One_wave,"Peak_Imax")==0)			sprintf Peak_Parameters_One, "\Z09\F'Arial'\rImax= %.1W1PA", Peak_Imax[Current_Peak]/1000000000000			Peak_Parameters_list+=Peak_Parameters_One		endif		If(cmpstr(One_wave,"Peak_Q")==0)			sprintf Peak_Parameters_One, "\Z09\F'Arial'\rQ= %.1W1PC", Peak_Q[Current_Peak]/1000000000000			Peak_Parameters_list+=Peak_Parameters_One		endif		If(cmpstr(One_wave,"Peak_Molec")==0)			sprintf Peak_Parameters_One, "\Z09\F'Arial'\rQ= %.3g Molec", Peak_Molec[Current_Peak]			Peak_Parameters_list+=Peak_Parameters_One		endif		If(cmpstr(One_wave,"Rise_time")==0)			sprintf Peak_Parameters_One, "\Z09\F'Arial'\rrise ("+num2str(Rise_Low_Prc)+"-"+num2str(Rise_Hi_Prc)+")= %.1W1Ps", Rise_time[Current_Peak]/1000			Peak_Parameters_list+=Peak_Parameters_One		endif		If(cmpstr(One_wave,"Rise_slope")==0)			sprintf Peak_Parameters_One, "\Z09\F'Arial'\rrise= %.1W1PA/ms", Rise_slope[Current_Peak]/1000000000000			Peak_Parameters_list+=Peak_Parameters_One		endif		If(cmpstr(One_wave,"Fall_time")==0)			sprintf Peak_Parameters_One, "\Z09\F'Arial'\rfall (75-25)= %.1W1Ps", Fall_time[Current_Peak]/1000			Peak_Parameters_list+=Peak_Parameters_One		endif		If(cmpstr(One_wave,"Fall_slope")==0)			SVAR Fit_method=Fit_method			Peak_Parameters_list+="\K(0,0,0)\r Fit: "+Fall_fit[Current_Peak]+"\K(0,0,52224)"			If (cmpstr(Fit_method,"Line")==0)				sprintf Peak_Parameters_One, "\Z09\F'Arial'\rfall= %.1W1PA/ms", Fall_slope[Current_Peak]/1000000000000			endif			If (cmpstr(Fit_method,"Exp")==0)				sprintf Peak_Parameters_One, "\Z09\F'Arial'\rfall (\F'Symbol't\F'Arial'1)= %.1W1Ps", Fall_slope[Current_Peak]/1000			endif			If (cmpstr(Fit_method,"DblExp")==0)				sprintf Peak_Parameters_One, "\Z09\F'Arial'\rfall (\F'Symbol't\F'Arial'1)= %.1W1Ps \rfall (\F'Symbol't\F'Arial'2)= %.1W1Ps", Fall_slope[Current_Peak]/1000, Fall_slope2[Current_Peak]/1000			endif			Peak_Parameters_list+=Peak_Parameters_One		endif		If(cmpstr(One_wave,"Foot_I")==0)			sprintf Peak_Parameters_One, "\Z09\F'Arial'\rFoot H= %.1W1PA", Foot_I[Current_Peak]/1000000000000			Peak_Parameters_list+=Peak_Parameters_One		endif		If(cmpstr(One_wave,"Foot_W")==0)			sprintf Peak_Parameters_One, "\Z09\F'Arial'\rFoot W= %.1W1Ps", Foot_W[Current_Peak]/1000			Peak_Parameters_list+=Peak_Parameters_One		endif		If(cmpstr(One_wave,"Foot_Q")==0)			sprintf Peak_Parameters_One, "\Z09\F'Arial'\rFoot Q= %.1W1PC", Foot_Q[Current_Peak]/1000000000000			Peak_Parameters_list+=Peak_Parameters_One		endif		If(cmpstr(One_wave,"Foot_Molec")==0)			sprintf Peak_Parameters_One, "\Z09\F'Arial'\rFoot Q= %.3g Molec", Foot_Molec[Current_Peak]			Peak_Parameters_list+=Peak_Parameters_One		endif		i+=1	while(1)	TextBox/W=Main_window/A=RT/C/N=Peak_data/F=1/G=(0,0,52224) Peak_Parameters_listEndFunction Draw_lines_All(Current_Peak)	Variable Current_Peak	SetDataFolder $"root:Quanta"	SVAR Zoom_On=Zoom_On	NVAR Total_peaks_number=Total_peaks_number	Wave Working_trace_copy=Working_trace_copy	Wave T_Max=T_Max	Wave Peak_Half_H1=Peak_Half_H1	Wave Peak_Half_H2=Peak_Half_H2	Wave Peak_Imax=Peak_Imax	Wave T_Bkg1=T_Bkg1	Wave Peak_Split1=Peak_Split1	Wave Peak_Split2=Peak_Split2	Wave Peak_t05=Peak_t05	Wave T_Bkg2=T_Bkg2	Wave Rise_Midpoint=Rise_Midpoint	Wave Foot_Q=Foot_Q	Wave Foot_W=Foot_W	Wave Foot_end=Foot_end	Wave Rise_Lowpnt_X=Rise_Lowpnt_X	Wave Rise_Hipnt_X=Rise_Hipnt_X	DoWindow/F Main_Window	PauseUpdate; Silent 1	GetAxis /Q left	Variable Mark_Height=(V_max-V_min)*0.04	SetDrawLayer /K UserFront	Delete_Tags()	Variable Peak_pnt=0	String TagName	NVAR Show_Legend=Show_Legend	if(Show_Legend==1)		Generate_annotation(Current_Peak)	endif	Do		Variable Max_X=T_Max[Peak_pnt]		Variable Start_X=Peak_Half_H1[Peak_pnt]		Variable End_X=Peak_Half_H2[Peak_pnt]		Variable Bkg1_location=T_Bkg1[Peak_pnt]		Variable Tail_location=T_Bkg2[Peak_pnt]		Variable Bottom_Y=min(Working_trace_copy(Bkg1_location),Working_trace_copy(Tail_location))		TagName="Max"+num2str(Peak_pnt)		Tag/C/N=$TagName Working_trace_copy, (Max_X), num2str(Peak_pnt+1)		Tag/C/N=$TagName /F=0 /X=0.00/Y=8		If(Peak_pnt==Current_Peak)			Tag/C/N=$TagName/G=(0,15872,65280) 		else 			Tag/C/N=$TagName /G=(0,0,0)/I=1		endif		String First_and_Last=Check_for_Separated_peaks(Peak_pnt)		Variable First_separated_pnt=str2num(StringFromList(0,First_and_Last))		Variable Last_separated_pnt=str2num(StringFromList(1,First_and_Last))		String Extrap_Wave="Extrap_"+num2str(Peak_pnt+1)		String Extrap_Trace=TraceNameList("Main_window", ";", 1 )		If(First_separated_pnt!=Last_separated_pnt)			If(strsearch(Extrap_Trace,Extrap_Wave,0)==-1)				If(WaveExists($Extrap_Wave))					AppendToGraph/W=Main_window $Extrap_Wave					ModifyGraph lstyle($Extrap_Wave)=2,lsize($Extrap_Wave)=0.5,rgb($Extrap_Wave)=(0,15872,65280)				endif			endif			If(Peak_pnt==First_separated_pnt)				SetDrawEnv xcoord=bottom, ycoord=left, linefgc= (0,15872,65280), dash= 2				DrawLine Bkg1_location,Working_trace_copy(Bkg1_location),Tail_location,Working_trace_copy(Tail_location)			endif		else			If(WaveExists($Extrap_Wave))				if (CheckName("Zoom_Win", 6)!=0)					RemoveFromGraph/Z/W=Zoom_Win $Extrap_Wave				endif				RemoveFromGraph/Z/W=Main_window $Extrap_Wave				KillWaves/Z $Extrap_Wave			endif			SetDrawEnv xcoord=bottom, ycoord=left, linefgc= (0,15872,65280), dash= 2			DrawLine Bkg1_location,Working_trace_copy(Bkg1_location),Tail_location,Working_trace_copy(Tail_location)		endif		SetDrawEnv xcoord=bottom, ycoord=left, linefgc= (0,15872,65280), dash= 2		DrawLine Start_X,(Working_trace_copy(Max_X)-Peak_Imax[Peak_pnt]/2),End_X,(Working_trace_copy(Max_X)-Peak_Imax[Peak_pnt]/2)			if(cmpstr(Zoom_On,"On")==0 )			SetDrawEnv xcoord=bottom, ycoord=left,  linefgc= (34816,34816,34816), arrow= 1			DrawLine Bkg1_location,(Working_trace_copy(Bkg1_location)-Mark_Height),Bkg1_location,(Working_trace_copy(Bkg1_location))			SetDrawEnv xcoord=bottom, ycoord=left,  linefgc= (34816,34816,34816), arrow= 1			DrawLine Tail_location,(Working_trace_copy(Tail_location)-Mark_Height),Tail_location,(Working_trace_copy(Tail_location))		endif		If (Foot_Q[Peak_pnt]!=0)			Variable Low_Rise_Pnt_X=Rise_Lowpnt_X[Peak_pnt]			Variable Low_Rise_Pnt_Y=Working_trace_copy(Rise_Lowpnt_X[Peak_pnt])			Variable Offset_Foot=Y_offset(T_Bkg1[Peak_pnt], T_Bkg2[Peak_pnt], Foot_end[Peak_pnt])			SetDrawEnv xcoord=bottom, ycoord=left, linefgc= (0,39168,0), dash= 2			DrawLine Low_Rise_Pnt_X,Low_Rise_Pnt_Y,Foot_end[Peak_pnt],Offset_Foot		endif		If (Peak_Split1[Peak_pnt]!=0)			Variable Offset_Split1=Y_offset(T_Bkg1[Peak_pnt], T_Bkg2[Peak_pnt], Peak_Split1[Peak_pnt])			SetDrawEnv xcoord=bottom, ycoord=left, linefgc=(24576,24576,65280), dash=0			DrawLine Peak_Split1[Peak_pnt],Working_trace_copy(Peak_Split1[Peak_pnt]),Peak_Split1[Peak_pnt],Offset_Split1		endif		If (Peak_Split2[Peak_pnt]!=0)			Variable Offset_Split2=Y_offset(T_Bkg1[Peak_pnt], T_Bkg2[Peak_pnt], Peak_Split2[Peak_pnt])			SetDrawEnv xcoord=bottom, ycoord=left, linefgc=(24576,24576,65280), dash=0			DrawLine Peak_Split2[Peak_pnt],Working_trace_copy(Peak_Split2[Peak_pnt]),Peak_Split2[Peak_pnt],Offset_Split2		endif		Peak_pnt+=1	while(Peak_pnt<Total_peaks_number)	if((cmpstr(Zoom_On,"On")==0)%|(CheckName("Zoom_Win", 6)!=0))		Variable Slope_coeffs=Fit_Rise(Rise_Lowpnt_X[Current_Peak],Rise_Hipnt_X[Current_Peak])		Wave/T Fall_fit=Fall_fit		String Fit_Function=Fall_fit[Current_Peak]		Fit_Function=Fit_Fall_Decay(Current_Peak,Fit_Function)	endif		if(cmpstr(Zoom_On,"On")==0 )		If (Foot_W[Current_Peak])			Tag/C/N=FootLoc Working_trace_copy, (Foot_end[Current_Peak]), "Foot"			Tag/C/N=FootLoc/F=0 /X=-2.4/Y=11.2 		endif	endif	if (CheckName("Zoom_Win", 6)!=0)		Draw_lines_zoom_window(Current_Peak)	endifendFunction Draw_lines_zoom_window(Peak_pnt)	Variable Peak_pnt	SetDataFolder $"root:Quanta"	SVAR Zoom_On=Zoom_On	Wave Working_trace_copy=Working_trace_copy	Wave Rise_phase=Rise_phase	Wave Fall_phase=Fall_phase	Wave T_Max=T_Max	Wave Peak_Half_H1=Peak_Half_H1	Wave Peak_Half_H2=Peak_Half_H2	Wave Peak_Imax=Peak_Imax	Wave T_Bkg1=T_Bkg1	Wave Peak_Split1=Peak_Split1	Wave Peak_Split2=Peak_Split2	Wave Peak_t05=Peak_t05	Wave T_Bkg2=T_Bkg2	Wave Rise_Midpoint=Rise_Midpoint	Wave Foot_Q=Foot_Q	Wave Foot_W=Foot_W	Wave Foot_end=Foot_end	Wave Rise_Lowpnt_X=Rise_Lowpnt_X	Wave Rise_Hipnt_X=Rise_Hipnt_X	Wave Foot_I=Foot_I	Variable Max_X=T_Max[Peak_pnt]	Variable Start_X=Peak_Half_H1[Peak_pnt]	Variable End_X=Peak_Half_H2[Peak_pnt]	Variable Bkg1_location=T_Bkg1[Peak_pnt]	Variable Tail_location=T_Bkg2[Peak_pnt]	Variable Bottom_Y=min(Working_trace_copy(Bkg1_location),Working_trace_copy(Tail_location))	PauseUpdate; Silent 1	Dowindow/F Zoom_Win	GroupBox Separator3,size={189,25},disable=1	Button FootDelete disable=1	Button FootNew_H disable=1	Button New_Rise disable=1	PopupMenu Fall_fit_change disable=1	SetVariable Extrap_Tau_Set disable=1	PopupMenu Fall_Extrap_change disable=1	Variable L_edge=(Bkg1_location-0.3*(Tail_location-Max_X))	Variable R_edge=(Tail_location+0.3*(Tail_location-Max_X))	Duplicate/O/R=(L_edge,R_edge) Working_trace_copy, Zoomed_peak	SetAxis left (Bottom_Y-0.2*Peak_Imax[Peak_pnt]),(Zoomed_peak(Max_X)+0.1*Peak_Imax[Peak_pnt])	SetAxis/A Bottom	Cursor A Zoomed_peak Bkg1_location;Cursor B Zoomed_peak Tail_location	PauseUpdate; Silent 1	SetDrawLayer /K UserFront	Tag/C/N=MaxLoc Zoomed_peak, (Max_X), "Max"	Tag/C/N=MaxLoc/F=0 /X=0.00/Y=8 	Tag/C/N=StartLoc Zoomed_peak, (Bkg1_location), "Start"	Tag/C/N=StartLoc/F=0 /X=0.00/Y=-10.00 	Tag/C/N=EndLoc Zoomed_peak, (Tail_location), "End"	Tag/C/N=EndLoc/F=0 /X=0.00/Y=-10.00 	String All_Traces=TraceNameList("Zoom_Win",";", 1)	Variable i=0	Do		String Extrap_Traces=Stringfromlist(i,All_Traces)		If(stringmatch(Extrap_Traces, "Extrap*")==1)			RemoveFromGraph/Z/W=Zoom_Win $Extrap_Traces		endif		i+=1		If(strlen(Extrap_Traces)==0)			break		endif	while(1)	String First_and_Last=Check_for_Separated_peaks(Peak_pnt)	Variable First_separated_pnt=str2num(StringFromList(0,First_and_Last))	Variable Last_separated_pnt=str2num(StringFromList(1,First_and_Last))	i=First_separated_pnt	If(First_separated_pnt!=Last_separated_pnt)		If(Peak_pnt==First_separated_pnt)			SetDrawEnv xcoord=bottom, ycoord=left, linefgc= (0,15872,65280), dash= 2			DrawLine Bkg1_location,Zoomed_peak(Bkg1_location),Tail_location,Zoomed_peak(Tail_location)		endif		Do			String Extrapolation_name="Extrap_"+num2str(i+1)			If(WaveExists($Extrapolation_name))				AppendToGraph/W=Zoom_Win $Extrapolation_name				ModifyGraph lstyle($Extrapolation_name)=2,lsize($Extrapolation_name)=0.5,rgb($Extrapolation_name)=(0,15872,65280)			endif			i+=1		while(i<=Last_separated_pnt)		SetAxis bottom L_edge,R_edge	else		SetDrawEnv xcoord=bottom, ycoord=left, linefgc= (0,15872,65280), dash= 2		DrawLine Bkg1_location,Zoomed_peak(Bkg1_location),Tail_location,Zoomed_peak(Tail_location)	endif	SetDrawEnv xcoord=bottom, ycoord=left, linefgc= (0,15872,65280), dash= 2	DrawLine Start_X,(Zoomed_peak(Max_X)-Peak_Imax[Peak_pnt]/2),End_X,(Zoomed_peak(Max_X)-Peak_Imax[Peak_pnt]/2)	If (Peak_Split1[Peak_pnt]!=0)		Bkg1_location=Peak_Split1[Peak_pnt]		Variable Offset_Split1=Y_offset(T_Bkg1[Peak_pnt], T_Bkg2[Peak_pnt], Peak_Split1[Peak_pnt])		SetDrawEnv xcoord=bottom, ycoord=left, linefgc=(24576,24576,65280), dash=0		DrawLine Peak_Split1[Peak_pnt],Working_trace_copy(Peak_Split1[Peak_pnt]),Peak_Split1[Peak_pnt],Offset_Split1	endif	If (Peak_Split2[Peak_pnt]!=0)		Tail_location=Peak_Split2[Peak_pnt]		Variable Offset_Split2=Y_offset(T_Bkg1[Peak_pnt], T_Bkg2[Peak_pnt], Peak_Split2[Peak_pnt])		SetDrawEnv xcoord=bottom, ycoord=left, linefgc=(24576,24576,65280), dash=0		DrawLine Peak_Split2[Peak_pnt],Working_trace_copy(Peak_Split2[Peak_pnt]),Peak_Split2[Peak_pnt],Offset_Split2	endif		If (Foot_W[Peak_pnt])		Variable Low_Rise_Pnt_X=Rise_Lowpnt_X[Peak_pnt]		Variable Low_Rise_Pnt_Y=Zoomed_peak(Rise_Lowpnt_X[Peak_pnt])		Variable Offset_Foot=Y_offset(T_Bkg1[Peak_pnt], T_Bkg2[Peak_pnt], Foot_end[Peak_pnt])		SetDrawEnv xcoord=bottom, ycoord=left, linefgc= (0,39168,0), dash= 2,linethick= 2.00		DrawLine Low_Rise_Pnt_X,Low_Rise_Pnt_Y,Foot_end[Peak_pnt],Offset_Foot		Tag/K/N=FootStart		Tag/C/N=FootEnd Zoomed_peak, (Foot_end[Peak_pnt]), "Foot"		Tag/C/N=FootEnd/F=0 /X=-10/Y=8 		ControlInfo /W=Zoom_win Zoom_to_Foot		If(V_Value==1)			Variable Rise_25=(Rise_Midpoint[Peak_pnt]+Rise_Lowpnt_X[Peak_pnt])/2			SetAxis left (Bottom_Y-0.1*Peak_Imax[Peak_pnt]),Zoomed_peak(Rise_25)			SetAxis bottom Bkg1_location,Rise_25			Variable Foot_Start_X=Foot_end[Peak_pnt]-Foot_W[Peak_pnt]/1000			Offset_Foot=Y_offset(T_Bkg1[Peak_pnt], T_Bkg2[Peak_pnt], Foot_Start_X)			SetDrawEnv xcoord=bottom, ycoord=left, linefgc= (0,39168,0), dash= 1,linethick= 1.00			DrawLine Foot_Start_X,(Zoomed_peak(Bkg1_location)+Foot_I[Peak_pnt]),Foot_Start_X,Offset_Foot			SetDrawEnv xcoord=bottom, ycoord=left, linefgc= (0,39168,0), dash= 1,linethick= 1.00			DrawLine Foot_end[Peak_pnt],Zoomed_peak(Foot_end[Peak_pnt]),Foot_end[Peak_pnt],Offset_Foot						SetDrawEnv xcoord=bottom, ycoord=left, linefgc= (0,39168,0), dash= 2,linethick= 2.00			DrawLine Foot_Start_X,(Zoomed_peak(Bkg1_location)+Foot_I[Peak_pnt]),Foot_end[Peak_pnt],(Zoomed_peak(Bkg1_location)+Foot_I[Peak_pnt])			Tag/C/N=FootStart Zoomed_peak, (Foot_Start_X), "Foot Start"			Tag/C/N=FootStart/F=0 /X=0/Y=15 			Tag/C/N=FootEnd Zoomed_peak, (Foot_end[Peak_pnt]), "Foot End"			Tag/C/N=FootEnd/F=0 /X=0/Y=15 			Tag/K/N=MaxLoc			Tag/K/N=EndLoc			FindLevel /Q/R=(Bkg1_location,Max_X ) Zoomed_peak, (Zoomed_peak(Bkg1_location)+Foot_I[Peak_pnt])			Cursor A Zoomed_peak V_LevelX;Cursor B Zoomed_peak Foot_end[Peak_pnt]			GroupBox Separator3,size={127,23},disable=0			Button FootDelete disable=0			Button FootNew_H disable=0			Button New_Rise disable=1		endif	else		Tag/K/N=FootStart		Tag/K/N=FootEnd	endif	ControlInfo /W=Zoom_win Zoom_to_Rise	If(V_Value==1)		SetAxis bottom Bkg1_location,Max_X		Cursor A Zoomed_peak Rise_Lowpnt_X[Peak_pnt];Cursor B Zoomed_peak Rise_Hipnt_X[Peak_pnt]		GroupBox Separator3,size={127,23},disable=0		Button FootDelete disable=0		Button FootNew_H disable=1		Button New_Rise disable=0	endif	ControlInfo /W=Zoom_win Zoom_to_Fall	If(V_Value==1)		Wave Fall_ChiRatio=Fall_ChiRatio		Wave/T Fall_fit=Fall_fit		Wave/T Fall_fit_Extrap		String Fit_method=Fall_fit[Peak_pnt]		NVAR Fall_Tau_Extrap=Fall_Tau_Extrap		PopupMenu Fall_fit_change disable=0		If(cmpstr(Fit_method,"Line")==0)			PopupMenu Fall_fit_change,mode=1,popvalue="Line", win=Zoom_Win		else			If(cmpstr(Fit_method,"Exp")==0)				PopupMenu Fall_fit_change,mode=2,popvalue="Exp", win=Zoom_Win			else				PopupMenu Fall_fit_change,mode=3,popvalue="DblExp", win=Zoom_Win				Fit_method="Chi2 ratio= "+num2str(Fall_ChiRatio[Peak_pnt])				TextBox/W=Zoom_Win/A=RT/C/N=Peak_data/F=0/G=(0,0,52224) Fit_method//				DrawRect 1.056,0.128,0.41,-0.0575			endif		endif		String Fit_Extrap_method=StringByKey("Fit", Fall_fit_Extrap[Peak_pnt])		If(cmpstr(Fit_Extrap_method,"")!=0)			PopupMenu Fall_Extrap_change disable=0			if(cmpstr(Fit_Extrap_method,"Line")!=0)				PopupMenu Fall_Extrap_change,mode=2,popvalue="Exp", win=Zoom_Win				Fall_Tau_Extrap=str2num(StringByKey("Tau", Fall_fit_Extrap[Peak_pnt]))				SetVariable Extrap_Tau_Set disable=0			else				PopupMenu Fall_Extrap_change,mode=1,popvalue="Line", win=Zoom_Win			endif			DrawRect 1.05,0.36,0.41,-0.0575		else			DrawRect 1.05,0.128,0.41,-0.0575		endif				SetAxis bottom Max_X,Tail_location	else		TextBox/K/W=Zoom_Win/N=Peak_data	endifEndFunction Delete_Tags()	SetDataFolder $"root:Quanta"	NVAR Total_peaks_number=Total_peaks_number	String TagName	Variable i=0	Do		TagName="Max"+num2str(i)		Tag/W=Main_window/K/N=$TagName		i+=1	while(i<Total_peaks_number+1)	Tag/W=Main_window/K/N=FootLoc	if (CheckName("Zoom_Win", 6)!=0)		DoWindow/F Zoom_Win		SetDrawLayer /K UserFront		Tag/K/N=FootStart		Tag/K/N=FootEnd		Tag/K/N=MaxLoc		Tag/K/N=StartLoc		Tag/K/N=EndLoc		TextBox/K/N=Peak_data		DoWindow/F Main_Window	endifEndFunction Y_offset(Bkg1, Bkg2, Med)	Variable Bkg1, Bkg2, Med	SetDataFolder $"root:Quanta"	Wave Working_trace_copy=Working_trace_copy	Variable Bkg_H=(Working_trace_copy(Bkg2)-Working_trace_copy(Bkg1))	Variable Bkg_W=(Bkg2-Bkg1)	Variable Offset=(Med-Bkg1)*Bkg_H/Bkg_W+Working_trace_copy(Bkg1)	return OffsetEndFunction Zoom_Trace_In_Out(theTag) : ButtonControl	String theTag	SetDataFolder $"root:Quanta"	SVAR Zoom_On=Zoom_On	NVAR Peak_ID=Peak_ID	NVAR X_min=X_min	NVAR X_max=X_max	NVAR Y_min=Y_min	NVAR Y_max=Y_max	NVAR Peak_ID=Peak_ID	NVAR Total_peaks_number=Total_peaks_number	If(Total_peaks_number==0)		abort	endif	strswitch (theTag)		case "See_all":			Button See_all rename=See_zoomed, title="Zoom Out"			Zoom_On ="On"			GetAxis /Q bottom			X_min=V_min			X_max=V_max			GetAxis /Q left			Y_min=V_min			Y_max=V_max			AppendToGraph Rise_phase,Fall_phase			ModifyGraph rgb(Rise_phase)=(0,0,0),rgb(Fall_phase)=(0,0,0)			break		case "See_zoomed":			Button See_zoomed rename=See_all, title="Zoom In"			Zoom_On ="Off"			SetAxis bottom X_min,X_max			SetAxis left Y_min,Y_max			RemoveFromGraph/Z Rise_phase,Fall_phase			break		case "See_foot":			break	endswitch	DoWindow/F Main_window	Peak_locator(Peak_ID-1)EndFunction Zoom_Down_Q(ctrlName) : ButtonControl	String ctrlName	Variable Min_Max_Delta	GetAxis /Q left	Min_Max_Delta=V_max-V_min	SetAxis left (V_min-Min_Max_Delta/4),(V_max+Min_Max_Delta/4)EndFunction Zoom_Up_Q(ctrlName) : ButtonControl	String ctrlName	Variable Min_Max_Delta	GetAxis /Q left	Min_Max_Delta=V_max-V_min	SetAxis left (V_min+Min_Max_Delta/5),(V_max-Min_Max_Delta/5)EndFunction Zoom_In_Horiz_Q(ctrlName) : ButtonControl	String ctrlName	Variable Min_Max_Delta	GetAxis /Q bottom	Min_Max_Delta=V_max-V_min	SetAxis bottom (V_min+Min_Max_Delta/6),(V_max-Min_Max_Delta/6)	Slider_Reset(ctrlName)EndFunction Zoom_OutHoriz_Q(ctrlName) : ButtonControl	String ctrlName	Variable Min_Max_Delta	GetAxis /Q bottom	Min_Max_Delta=V_max-V_min	SetAxis bottom (V_min-Min_Max_Delta/5),(V_max+Min_Max_Delta/5)	Slider_Reset(ctrlName)EndFunction Move_Up_Q(ctrlName) : ButtonControl	String ctrlName	Variable Min_Max_Delta	GetAxis /Q left	Min_Max_Delta=V_max-V_min	SetAxis left (V_min+Min_Max_Delta/6),(V_max+Min_Max_Delta/6)EndFunction Move_Down_Q(ctrlName) : ButtonControl	String ctrlName	Variable Min_Max_Delta	GetAxis /Q left	Min_Max_Delta=V_max-V_min	SetAxis left (V_min-Min_Max_Delta/6),(V_max-Min_Max_Delta/6)EndFunction Move_Left_Q(ctrlName) : ButtonControl	String ctrlName	Variable Min_Max_Delta	GetAxis /Q bottom	Min_Max_Delta=V_max-V_min	SetAxis bottom (V_min-Min_Max_Delta/2),(V_max-Min_Max_Delta/2)	Slider_Reset(ctrlName)EndFunction Move_Right_Q(ctrlName) : ButtonControl	String ctrlName	Variable Min_Max_Delta	GetAxis /Q bottom	Min_Max_Delta=V_max-V_min	SetAxis bottom (V_min+Min_Max_Delta/2),(V_max+Min_Max_Delta/2)	Slider_Reset(ctrlName)EndFunction Slider_Reset(ctrlName) : ButtonControl	String ctrlName	SetDataFolder $"root:Quanta"	GetAxis/W=Main_window /Q bottom	Variable dx= (V_max+V_min)/2	NVAR T_Start=T_Start	NVAR T_End=T_End	Variable Slider_value=(dx-T_Start)/(T_End-T_Start)	Slider X_Slider,value= Slider_value,win=Main_windowEndFunction Slider_Horiz_Q(TheTag, Value, event)	String TheTag	Variable Value	Variable event	SetDataFolder $"root:Quanta"	String Traces_Names=TraceNameList("","",1)	String Trace_Name=StringFromList(0,Traces_Names)	Variable First_X=pnt2x($Trace_Name,0)	Variable Last_X=pnt2x($Trace_Name,numpnts($Trace_Name))	GetAxis/Q bottom	Variable Med_X= Value*(Last_X-First_X)+First_X	Variable dX= (V_max-V_min)/2	SetAxis bottom,Med_X-dX,Med_X+dX	return 0End//___________________________________//______Load, Save, Delete Traces________//___________________________________Window File_Q() : Panel	PauseUpdate; Silent 1	NewPanel /W=(4,50,729,76) as "Menu"	SetDrawLayer UserBack	SetDrawEnv fillfgc= (39168,0,0)	DrawRect 2,25,242,0	Button Load_File,pos={5,3},size={83,20},proc=File_macro_Q,title="Open File"	PopupMenu Recent_files,pos={89,2},size={72,21},proc=Recent_files_list_Q,title="Recent"	PopupMenu Recent_files,mode=0,value= #"root:Quanta:File_list"	Button Reuse,pos={170,3},size={70,20},proc=File_macro_Q,title="Revert"	SetDrawEnv fillfgc= (0,26112,0)	DrawRect 244,0,550,25	Button Menu_FiltersScales,pos={247,3},size={80,20},proc=Show_options_panel,title="Filters/Scales"	Button Menu_Cutoffs,pos={328,3},size={73,20},proc=Show_options_panel,title="Cutoffs"	Button Menu_Results,pos={402,3},size={73,20},proc=Show_options_panel,title="Results"	Button Menu_Stats,pos={475,3},size={73,20},proc=Show_options_panel,title="Stats"	SetDrawEnv fillpat= 0	DrawRect 552,0,723,25	Button Legend_On,pos={554,3},size={83,20},proc=Show_Extras,title="Hide Legend"	Button Zoom_Off,pos={638,3},size={83,20},proc=Show_Extras,title="Zoom Win"EndMacroMacro Load_single_file_Q(From_the_list)	Variable From_the_list	Close /A	SetDataFolder $"root:Quanta"	Variable/G Peak_ID	Variable/G Total_peaks_number	Variable/G T_Start_orig	Variable/G T_Delta_orig	String/G File_to_load	String/G Loaded_file_path	String/G Zoom_On	variable RefNumber	If(From_the_list==1)		KillWaves/Z Orig_trace_copy		LoadWave/Q/H/O File_to_load		Loaded_file_path=File_to_load	else		If(cmpstr(igorInfo(2),"Macintosh")==0)			DoAlert 2, "Is it a Macintosh file You are trying to open?\r Press 'No' if the recording was made on a PC"			If (V_flag==1)				Open/D/R/T="IGBW" RefNumber			else				If (V_flag==2)					Open/D/R/T="????" RefNumber				else					abort				endif			endif		else			Open/D/R/T="bwav" RefNumber		endif					if(strlen(S_filename)==0)			abort		endif		Loaded_file_path=S_fileName				KillWaves/Z Orig_trace_copy		LoadWave/Q/H/O Loaded_file_path	endif	Dowindow/F Main_window	De_novo()	String Orig_Trace_name=StringFromList(0,S_waveNames,";")	Duplicate/O $Orig_Trace_name Orig_trace_copy	KillWaves/Z $Orig_Trace_name	T_Start_orig=pnt2x(Orig_trace_copy,0)	T_Delta_orig=(pnt2x(Orig_trace_copy,1)-pnt2x(Orig_trace_copy,0))*1000	String info =WaveInfo(Orig_trace_copy, 0)	String X_scale_units=StringByKey("XUNITS", info)		If(cmpstr(X_scale_units,"ms")==0)		T_Delta_orig=T_Delta_orig/1000	endif		If(cmpstr(X_scale_units,"min")==0)		T_Delta_orig=T_Delta_orig*1000*60	endif		Copy_Orig_Wave()	String/G Loaded_file_name=S_filename	If(strlen(Loaded_file_name)>40)		Loaded_file_name=Long_Name_Cut(Loaded_file_name)	endif	DoWindow/T Main_window, Loaded_file_name	If (CheckName("See_zoomed", 15)!=0)		Button See_zoomed rename=See_all, title="Zoom In"		Zoom_On ="Off"	endif	If(Check_files_list_Q(Loaded_file_path)==0)		String/G File_list=File_list+";"+Loaded_file_path		String Too_much=StringFromList(21,File_list)		if( strlen(Too_much) != 0 )			string First_item=StringFromList(1,File_list)			File_list=RemoveFromList(First_item, File_list)		endif		if (CheckName("File",9)!=0)			PopupMenu Recent_files,mode=0,value= root:Quanta:File_list,win=File		endif	endifEndmacroFunction/S Long_Name_Cut(Name)	String Name	Prompt Name, "New name (less than 40 characters)"	DoPrompt "Window name is too long!", Name	Return NameEndFunction Copy_Orig_Wave()	SetDataFolder $"root:Quanta"	NVAR Gain=Gain	NVAR Gain_Temp=Gain_Temp	NVAR T_Start_orig=T_Start_orig	NVAR T_Delta_orig=T_Delta_orig	NVAR T_Start=T_Start	NVAR T_End=T_End	NVAR T_Delta=T_Delta	NVAR X_min=X_min	NVAR X_max=X_max	NVAR Y_min=Y_min	NVAR Y_max=Y_max	NVAR Bkg_noise_I=Bkg_noise_I	NVAR Bkg_noise_dI=Bkg_noise_dI	NVAR Bkg_noise_Start=Bkg_noise_Start	NVAR Bkg_noise_End=Bkg_noise_End	NVAR Overall_Filter=Overall_Filter	SVAR Zoom_On=Zoom_On	Wave Orig_trace_copy=Orig_trace_copy		Overall_Filter=0	Orig_trace_copy*=Gain	If(numpnts(Orig_trace_copy)==0)		abort "The trace you are trying to load has 0 datapoints!"	endif	Duplicate/O Orig_trace_copy Working_trace_copy	Make/O/N=1 Zoomed_peak, Fall_phase,Rise_phase	If(cmpstr(Zoom_On,"On")==0)		Button See_zoomed win=Main_window, rename=See_all, title="Zoom In"		Zoom_On ="Off"		RemoveFromGraph/W=Main_window/Z Rise_phase,Fall_phase	endif	T_Start=T_Start_orig	T_Delta=T_Delta_orig	SetScale/P x T_Start,T_Delta/1000,"s", Working_trace_copy	SetScale d 0,0,"pA", Working_trace_copy	T_End=pnt2x(Working_trace_copy, (numpnts(Working_trace_copy)-1))	X_min=T_Start	X_max=T_End	SetAxis/W=Main_window/A	DoUpdate	GetAxis/W=Main_window/Q left	Y_min=V_min	Y_max=V_max	Slider X_Slider, win=Main_window,value=0.5	GroupBox Bkg_HiLt, win=Main_window,disable=0	Filter_Limits()	Bkg_noise_I=0	Bkg_noise_dI=0	Bkg_noise_Start=0	Bkg_noise_End=0EndFunction File_macro_Q(theTag) : ButtonControl	String theTag	SetDataFolder $"root:Quanta"	NVAR Total_peaks_number=Total_peaks_number	NVAR Smoothing_Factor_diff1=Smoothing_Factor_diff1	NVAR Gain=Gain	NVAR T_Start_orig=T_Start_orig	NVAR T_delta_orig=T_delta_orig	if( cmpstr(theTag,"Load_File")==0 )		execute "load_single_file_Q(0)"	endif	if( cmpstr(theTag,"Reuse")==0 )		Wave Orig_trace_copy=Orig_trace_copy		Wave Working_trace_copy=Working_trace_copy		if(Total_peaks_number)			DoAlert 1, "All detected spikes will be deleted!!! \rDo you really wanna to kill them all!?"			If (V_Flag==2)				abort			endif		else			If(numpnts(Working_trace_copy)<2)				abort "No trace loaded or the wave is too short."			endif		endif		SetScale/P x T_Start_orig,T_Delta_orig/1000,"s", Orig_trace_copy		Orig_trace_copy/=Gain		De_novo()		Copy_Orig_Wave()	endifEndFunction Check_files_list_Q(File_name)	String File_name	SetDataFolder $"root:Quanta"	SVAR File_list=File_list	String File	Variable i=1	do		File=StringFromList(i,File_list)		if( strlen(File) == 0 )			return 0			break		else			If (cmpstr(File_name,File)==0)				return 1				break			endif		endif		i += 1	while (1)EndFunction Recent_files_list_Q(ctrlName,popNum,popStr) : PopupMenuControl	String ctrlName	Variable popNum	String popStr	SetDataFolder $"root:Quanta"	SVAR File_list=File_list	String/G File_to_load	File_to_load=popStr	execute "Load_single_file_Q(1)"EndFunction Save_zoomed_trace(ctrlName) : ButtonControl	String ctrlName	SetDataFolder $"root:Quanta"	Wave Working_trace_copy=Working_trace_copy	If(numpnts(Working_trace_copy)<2)		abort "No trace loaded or the wave is too short."	endif	SVAR Loaded_file_name=Loaded_file_name	String Name_to_save=Loaded_file_name[0,(strsearch(Loaded_file_name, ".", 0)-1)]	If(cmpstr(ctrlName,"Save_Avg")==0)		NVAR  Avg_Spike_Weight=Avg_Spike_Weight		Duplicate/O Avg_peak Avg_peak_saved		Name_to_save+="_Avg_N"+num2str(Avg_Spike_Weight)		Save/C/I Avg_peak_saved as Name_to_save		KillWaves/Z Avg_peak_saved		return 0	endif	GetAxis /Q bottom	variable Start_X=V_min	variable End_X=V_max	Duplicate/O/R=(Start_X,End_X) Working_trace_copy zoomed_trace	Name_to_save+="_Zoom"	Save/C/I zoomed_trace as Name_to_saveEndFunction De_novo()	SetDataFolder $"root:Quanta"	NVAR Peak_ID=Peak_ID	NVAR Total_peaks_number=Total_peaks_number	Delete_Tags()	TextBox/W=Main_window/N=Peak_data/K	Change_waves("Make",0)	Total_peaks_number=0	Peak_ID=0	Dowindow/F Main_window	RemoveFromGraph/Z/W=Main_window Rise_phase,Fall_phase	Make/O/N=1 Zoomed_peak, Fall_phase,Rise_phase	SetVariable ID,limits={1,(Total_peaks_number),1},win=Main_window	SetDrawLayer /K UserFront	if (CheckName("Zoom_Win", 6)!=0)		SetVariable ID,limits={1,(Total_peaks_number),1},win=Zoom_Win	endif	String Extrap_	String Extrap_waves=WaveList("Extrap*",";","")	Variable q=0	Do		Extrap_=Stringfromlist(q,Extrap_waves)		If(strlen(Extrap_)==0)			break		endif		if (CheckName("Zoom_Win", 6)!=0)			RemoveFromGraph/Z/W=Zoom_Win $Extrap_		endif		RemoveFromGraph/Z/W=Main_window $Extrap_		KillWaves /Z $Extrap_		q+=1	while(1)EndFunction Change_Waves(ToDo,pnt)	String ToDo	Variable pnt	SetDataFolder $"root:Quanta"	String/G All_waves=" Peak_Num,T_Max,Peak_Base,Peak_Imax,Peak_t05,Peak_Q,Peak_Molec,Rise_slope,Fall_slope,Fall_slope2,"	All_waves+="Peak_Half_H1,Peak_Half_H2, T_Bkg1,T_Bkg2,Rise_Midpoint,Rise_time,Fall_time,Interspike_interval,"	All_waves+="Peak_Split2, Peak_Split1,Foot_Q,Foot_Molec,Foot_W,Foot_end, Rise_Lowpnt_X, Rise_Hipnt_X, Foot_I, Fall_ChiRatio"	String Exe	if( cmpstr(ToDo,"Make")==0)		Exe="Make/O/N="+num2str(pnt)+All_waves		execute Exe		Exe="Make/O/T/N="+num2str(pnt)+"Fall_fit, Fall_Fit_Extrap"		execute Exe	endif	if( cmpstr(ToDo,"Redimension")==0 )		Exe="Redimension/N="+num2str(pnt)+All_waves		execute Exe		Exe="Redimension/N="+num2str(pnt)+"Fall_fit, Fall_Fit_Extrap"		execute Exe	endif	String New_Name="", Extrap_waves=""	Variable Name_Ln, Extrap_Num,q	if( cmpstr(ToDo,"Delete")==0 )		String Extrap_Exists="Extrap_"+num2str(pnt+1)		If(exists(Extrap_Exists)==1)			if (CheckName("Zoom_Win", 6)!=0)				RemoveFromGraph/Z/W=Zoom_Win $Extrap_Exists			endif			RemoveFromGraph/Z/W=Main_window $Extrap_Exists			KillWaves/Z $Extrap_Exists		endif		Extrap_Exists="Extrap_"+num2str(pnt)		If(exists(Extrap_Exists)==1)			if (CheckName("Zoom_Win", 6)!=0)				RemoveFromGraph/Z/W=Zoom_Win $Extrap_Exists			endif			RemoveFromGraph/Z/W=Main_window $Extrap_Exists			KillWaves/Z $Extrap_Exists		endif		Exe="DeletePoints "+num2str(pnt)+",1, "+All_waves		execute Exe		Exe="DeletePoints "+num2str(pnt)+",1, Fall_fit, Fall_Fit_Extrap"		execute Exe				Extrap_waves=WaveList("Extrap*",";","")		q=0		Do			Extrap_Exists=Stringfromlist(q,Extrap_waves)			Name_Ln=strlen(Extrap_Exists)			If(Name_Ln==0)				break			endif			Extrap_Num=str2num(Extrap_Exists[7,Name_Ln])				If(Extrap_Num>=pnt+1)				New_Name="Extrap_"+num2str(Extrap_Num-1)				Rename $Extrap_Exists, $New_Name			endif			q+=1		while(1)	endif	if(cmpstr(ToDo,"Insert")==0 )		Exe="InsertPoints "+num2str(pnt)+",1, "+All_waves		execute Exe		Exe="InsertPoints "+num2str(pnt)+",1, Fall_fit, Fall_Fit_Extrap"		execute Exe			Extrap_waves=WaveList("Extrap*",";","")		q=ItemsInList(Extrap_waves)-1		Do			Extrap_Exists=Stringfromlist(q,Extrap_waves)			Name_Ln=strlen(Extrap_Exists)			If(Name_Ln==0)				break			endif			Extrap_Num=str2num(Extrap_Exists[7,Name_Ln])				If(Extrap_Num>=pnt+1)				New_Name="Extrap_"+num2str(Extrap_Num+1)				Rename $Extrap_Exists, $New_Name			endif			q-=1		while(1)	endifEnd//__________________________________//___________Averaged Spike _________//__________________________________Function Stats_PopMenu(theTag,popNum,popStr) : PopupMenuControl	String theTag	Variable popNum	String popStr	SetDataFolder $"root:Quanta"	SVAR Norm_point=Norm_point	SVAR Population_Center=Population_Center	strswitch(theTag)		case "Stats_Population":			Population_Center=popStr			break		case "Stats_AvePeak":			Norm_point=popStr			break	endswitchEndFunction Average_peaks(ctrlName) : ButtonControl	String ctrlName	SetDataFolder $"root:Quanta"	NVAR Total_peaks_number=Total_peaks_number	Wave Rise_Midpoint=Rise_Midpoint	Wave T_Max=T_Max	Wave T_Bkg1=T_Bkg1	Wave T_Bkg2=T_Bkg2	Wave Peak_Split1=Peak_Split1	Wave Peak_Split2=Peak_Split2	Wave Fall_slope=Fall_slope	Wave Fall_slope2=Fall_slope2	Wave Working_Trace_Copy=Working_Trace_Copy	NVAR Bkg_noise_I=Bkg_noise_I	NVAR T_Delta=T_Delta	NVAR Overall_Filter=Overall_Filter	Variable/G Avg_Spike_Weight	if (CheckName("Avg_peak_graph", 6)==0)		execute "Avg_peak_graph()"	else		Dowindow/F Avg_peak_graph		MoveWindow 1, 1, 1, 1	endif	if (CheckName("Avg_peak_pnts", 6)==0)		PauseUpdate; Silent 1		Edit/K=1/W=(420,259.25,580.5,482.75) Avg_peak.xy as "Averaged wave"		String command="ModifyTable width(Point)=0,size(Avg_peak.xy)=9,width(Avg_peak.xy)=68"		execute command		DoWindow/C Avg_peak_pnts		AutoPositionWindow/E/M=1/R=Avg_peak_graph Avg_peak_pnts	else		Dowindow/F Avg_peak_pnts		MoveWindow 1, 1, 1, 1	endif	SetWindow Avg_peak_graph, hook=$"Killer_of_Hookers"	SetWindow Avg_peak_pnts, hook=Killer_of_Hookers	if(Total_peaks_number==0)		abort	endif	If(numpnts(Avg_peak)!=0)		DoAlert 1, "Discard existing averaged spike?"		if(V_flag==1)			Make/O/N=0 Avg_peak		else			abort		endif	endif	Variable Binomial_coeff=Gaussian_to_Binomial_Calc(Overall_Filter)	Variable S=0	SVAR Norm_point=Norm_point	If(cmpstr(Norm_point,"Max")==0)		Wave Norm_data=T_Max	else		Wave Norm_data=Rise_Midpoint	endif	Variable Start_P_longest, End_P_longest	Duplicate/O T_Max, Limit_L, Limit_R	Do		If(Peak_Split1[S])			Limit_L[S]=x2pnt(Working_Trace_Copy,Norm_data[S])-x2pnt(Working_Trace_Copy,Peak_Split1[S])		else			Limit_L[S]=x2pnt(Working_Trace_Copy,Norm_data[S])-x2pnt(Working_Trace_Copy,T_Bkg1[S])		endif		Start_P_longest=max(Start_P_longest,Limit_L[S])		If(Peak_Split2[S])			Limit_R[S]=x2pnt(Working_Trace_Copy,Peak_Split2[S])-x2pnt(Working_Trace_Copy,Norm_data[S])		else			If(S==Total_peaks_number-1)				Limit_R[S]=x2pnt(Working_Trace_Copy,T_Bkg2[S])-x2pnt(Working_Trace_Copy,Norm_data[S])			else				Limit_R[S]=x2pnt(Working_Trace_Copy,min(T_Bkg2[S], T_Bkg1[S+1]))-x2pnt(Working_Trace_Copy,Norm_data[S])			endif		endif		End_P_longest=max(End_P_longest,Limit_R[S])		S+=1	while(S<Total_peaks_number)		Variable Norm_pnt=x2pnt(Working_Trace_Copy,Norm_data[1])	Duplicate/O/R=[Norm_pnt-Start_P_longest,Norm_pnt+End_P_longest] Working_trace_copy, Avg_peak	SetScale/P x -Start_P_longest*(T_Delta/1000),(T_Delta/1000), "s", Avg_peak	Avg_peak=0	S=0	Variable Bkg_level	String Extrap_Name	Do		Norm_pnt=x2pnt(Working_Trace_Copy,Norm_data[S])		Duplicate/O/R=[Norm_pnt-Start_P_longest-1,Norm_pnt+End_P_longest] Working_trace_copy, Temp_wave		CopyScales/P Avg_peak Temp_wave		Bkg_level=Temp_wave[Start_P_longest-1-Limit_L[S]]		Temp_wave-=Bkg_level			Temp_wave[0,Start_P_longest-1-Limit_L[S]]=0		Duplicate/O/R=[0,Start_P_longest-1-Limit_L[S]] Temp_wave Noizy_Inset		Noizy_Inset+=gnoise(Bkg_noise_I*2)		if(numpnts(Noizy_Inset)>Binomial_coeff+1)			Smooth Binomial_coeff, Noizy_Inset		endif		Temp_wave[0,Start_P_longest-1-Limit_L[S]]+=Noizy_Inset(x)		Temp_wave[Start_P_longest-1+Limit_R[S],numpnts(Temp_wave)]=0		Duplicate/O/R=[Start_P_longest-1+Limit_R[S],numpnts(Temp_wave)] Temp_wave Noizy_Inset		Noizy_Inset+=gnoise(Bkg_noise_I*2)		if(numpnts(Noizy_Inset)>Binomial_coeff+1)			Smooth Binomial_coeff, Noizy_Inset		endif		Temp_wave[Start_P_longest-1+Limit_R[S],numpnts(Temp_wave)]+=Noizy_Inset(x)		Extrap_Name="Extrap_"+num2str(S+1)		If(exists(Extrap_Name)==1)			Duplicate/O $Extrap_Name, qqq			qqq-=Bkg_level			SetScale/P x pnt2x(Temp_wave,(Start_P_longest-1+Limit_R[S])),(T_Delta/1000), "s", qqq			Temp_wave[Start_P_longest-1+Limit_R[S],Start_P_longest-1+Limit_R[S]+numpnts(qqq)]+=qqq(x)		endif		CopyScales/P Avg_peak Temp_wave		Extrap_Name="Extrap_"+num2str(S)		If(exists(Extrap_Name)==1)			Duplicate/O $Extrap_Name, qqq			Bkg_level=max(qqq(0),qqq(numpnts(qqq)))			qqq-=Bkg_level			CopyScales/P Temp_wave qqq			Temp_wave[0,numpnts(qqq)]-=qqq(x)		endif//		string namen="Temp_wave"+num2str(S)//		Duplicate/O Temp_wave $namen		Avg_peak+=Temp_wave(x)		S+=1	While(S<Total_peaks_number)	Avg_peak/=S	Avg_Spike_Weight=S		Killwaves/Z Limit_L, Limit_R,qqqEndMacro Avg_peak_graph() : Graph	PauseUpdate; Silent 1	SetDataFolder $"root:Quanta"	Display/K=1/W=(419.25,37.25,579.75,228.5) Avg_peak as "Averaged Spike"	DoWindow/C Avg_peak_graph	ControlBar 45	Button Smooth,pos={1,3},size={50,20},proc=SmoothBtn_Q,title="Smooth"	SetVariable Smooth_F,pos={157,5},size={53,16},title=" "	SetVariable Smooth_F,limits={1,32767,10},value= root:Quanta:Smoothing_Factor	PopupMenu Smoth_meth,pos={56,2},size={76,21},proc=Smooth_method_Q	PopupMenu Smoth_meth,mode=3,popvalue="Binomial sm. ",value= #"\"LP Gaussian ;HP Gaussian ;Binomial sm. ;Boxcar sm.   ;Sav.-Gol. sm.\""	Button Add_Avg,pos={1,25},size={65,18},proc=Add_saved_avg,title="Add"	Button Add_Avg,help={"You can sum up averaged peaks from several experiments later!"}	Button Del_Avg,pos={68,25},size={65,18},proc=Delete_Avg,title="Clear"	Button Save_Avg,pos={144,48},size={65,18},proc=Save_zoomed_trace,title="Save"	Button Save_Avg,help={"You can sum up averaged spikes from several experiments later!"}	ValDisplay Avg_total_W title="N=",pos={138,26},size={70,15},value=root:Quanta:Avg_Spike_Weight	ModifyGraph zero(bottom)=2	AutoPositionWindow/E/M=0/R=File_Q Avg_peak_graphEndmacroFunction Delete_Avg(ctrlName) : ButtonControl	String ctrlName	SetDataFolder $"root:Quanta"	Wave Avg_peak=Avg_peak	NVAR  Avg_Spike_Weight=Avg_Spike_Weight	DoAlert 1, "Delete averaged spike?"	if(V_flag==2)		abort	endif	Make/O/N=0 Avg_peak	Avg_Spike_Weight=0EndProc Get_weights(Weight_Added)	Variable Weight_Added=gWeight_Added	Prompt Weight_Added, "Enter the number of averaged spikes in the Added trace:" 	gWeight_Added=Weight_AddedEndFunction Add_saved_avg(ctrlName) : ButtonControl	String ctrlName	SetDataFolder $"root:Quanta"	Variable/G gWeight_Added	Wave Avg_peak=Avg_peak	NVAR  Avg_Spike_Weight=Avg_Spike_Weight	Close /A	variable RefNumber	Open/D/R/T="IGBW" RefNumber	if(strlen(S_filename)==0)		abort	endif	String Loaded_wave=S_fileName	LoadWave/Q/H/O Loaded_wave	String Added_Trace_name=StringFromList(0,S_waveNames,";")	Execute "Get_weights()"	Wave Added_wave=$Added_Trace_name		If(numpnts(Avg_peak)==0)		Duplicate/O Added_wave Avg_peak		Avg_Spike_Weight+=gWeight_Added		abort	endif			Avg_peak*=Avg_Spike_Weight	Added_wave*=gWeight_Added		Variable Org_DeltaT=pnt2x(Avg_peak,1)-pnt2x(Avg_peak,0)	Variable Added_DeltaT=pnt2x(Added_wave,1)-pnt2x(Added_wave,0)	Variable Org_Npnts=x2pnt(Avg_peak,0)	Variable Added_Npnts=x2pnt(Added_wave,0)	If(Org_Npnts>Added_Npnts)		SetScale/P x pnt2x(Avg_peak,0)+(Org_Npnts-Added_Npnts)*Org_DeltaT,Org_DeltaT, "s", Avg_peak		DeletePoints 0,(Org_Npnts-Added_Npnts), Avg_peak	else		SetScale/P x pnt2x(Added_wave,0)+(Added_Npnts-Org_Npnts)*Added_DeltaT,Added_DeltaT, "s", Added_wave		DeletePoints 0,(Added_Npnts-Org_Npnts), Added_wave	endif			Org_Npnts=numpnts(Avg_peak)-x2pnt(Avg_peak,0)	Added_Npnts=numpnts(Added_wave)-x2pnt(Added_wave,0)	If(Org_Npnts>Added_Npnts)		DeletePoints numpnts(Added_wave),(Org_Npnts-Added_Npnts), Avg_peak	else		DeletePoints numpnts(Avg_peak),(Added_Npnts-Org_Npnts), Added_wave	endif		Avg_peak+=Added_wave	Avg_peak/=(Avg_Spike_Weight+gWeight_Added)	Avg_Spike_Weight+=gWeight_Added	KillWaves/Z Added_waveEndFunction Killer_of_Hookers(infoStr)	String infoStr		Variable somethingDone=0	String win = StringByKey("WINDOW",infoStr)	String event = StringByKey("EVENT",infoStr)		if (CmpStr(event, "kill") != 0)		return 0	endif	if (CmpStr(win, "Avg_peak_graph") != 0)		DoWindow/K Avg_peak_graph		somethingDone=1	endif	if (CmpStr(win, "Avg_peak_pnts") != 0)		DoWindow/K Avg_peak_pnts		somethingDone=1	endif	return somethingDoneend//__________Stats Notebook____________Function Show_stats(ctrlName) : ButtonControl	String ctrlName	if (CheckName("Stats", 10)==0)		NewNotebook/N=Stats/F=1/V=1/W=(2.4,121.4,604.2,305) as "Data analysis"		Generate_notebook()	else		DoWindow /F Stats 		MoveWindow 1,1,1,1		Notebook Stats selection={startOfFile, endOfFile}		Generate_notebook()	endifEndFunction Generate_notebook()	SetDataFolder $"root:Quanta"	Wave T_Max=T_Max	SVAR Loaded_file_path=Loaded_file_path	String Row_to_Print	String/G One_wave	String Stat_result	NVAR Total_peaks_number=Total_peaks_number	NVAR Overall_Filter=Overall_Filter	NVAR Bkg_noise_I=Bkg_noise_I	NVAR Bkg_noise_dI=Bkg_noise_dI	NVAR Detection_Mult=Detection_Mult	NVAR Detection_Foot_Mult=Detection_Foot_Mult	NVAR Smoothing_Factor_Add=Smoothing_Factor_Add	NVAR Smoothing_Factor_diff1=Smoothing_Factor_diff1	NVAR Smooth_more=Smooth_more	NVAR Smooth_Derivative=Smooth_Derivative	NVAR Spike_Min_Imax=Spike_Min_Imax	NVAR Spike_Max_T05=Spike_Max_T05	NVAR Spike_Max_Trise=Spike_Max_Trise	NVAR Foot_Min_W=Foot_Min_W	NVAR Foot_Min_H=Foot_Min_H	NVAR SSFoot_Do=SSFoot_Do	NVAR Native_Foot_Del=Native_Foot_Del	NVAR Baseline_Drift=Baseline_Drift	NVAR Overlap_Prc=Overlap_Prc	SVAR Overlaps=Overlaps	SVAR Values_to_show=Values_to_show	SVAR Stats_names=Stats_names	SVAR Population_Center=Population_Center		String nb = "Stats"	Notebook $nb defaultTab=36, statusWidth=238, pageMargins={72,72,72,72}	Notebook $nb showRuler=1, rulerUnits=1, updating={1, 3600}	Notebook $nb newRuler=Normal, justification=0, margins={0,0,468}, spacing={0,0,0}, tabs={}, rulerDefaults={"Arial",7,0,(0,0,0)}	Notebook $nb ruler=Normal, specialChar={3,0,""}	Notebook $nb text="\r"	Notebook $nb text=Loaded_file_path+"\r"	Notebook $nb text="Trace filtering (-3dB Gaussian):\r"	Notebook $nb text="\tCurrent trace - "+num2str(Overall_Filter)+" Hz\r"	If(Smooth_more)		Notebook $nb text="\tAdditional trace filtering - "+num2str(Smoothing_Factor_Add)+" Hz\r"	endif	If(Smooth_Derivative)		Notebook $nb text="\tDifferentiated trace filtering - "+num2str(Smoothing_Factor_diff1)+" Hz\r"	endif	Notebook $nb text="Noise level: SD(I) = "+num2str(Bkg_noise_I)+";  SD(dI/dt) = "+num2str(Bkg_noise_dI)	Notebook $nb text="\rDetection threshold: Spikes - SD(dI/dt)*"+num2str(Detection_Mult)+"; Foot -  SD(I)*"+num2str(Detection_Foot_Mult)	Notebook $nb text="\rCutoffs used:\r"	If(Spike_Min_Imax)		Notebook $nb text="\tMin I(max) - "+num2str(Spike_Min_Imax)+" pA\r"	endif	If(Spike_Max_T05)		Notebook $nb text="\tMax T(1/2) - "+num2str(Spike_Max_T05)+" ms\r"	endif	If(Spike_Max_Trise)		Notebook $nb text="\tMax T(rise) - "+num2str(Spike_Max_Trise)+" ms\r"	endif	If(Foot_Min_H)		Notebook $nb text="\tMin I(foot) - "+num2str(Foot_Min_H)+" pA\r"	endif	If(SSFoot_Do)		Notebook $nb text="\tOnly PSF with steady states longer than "+num2str(Foot_Min_W)+" ms were analized\r"	else		If(Foot_Min_W)			Notebook $nb text="\tMin T(foot) - "+num2str(Foot_Min_W)+" ms\r"		endif	endif	If(Native_Foot_Del)		Notebook $nb text="\t'Native' PSF were deleted\r"	endif	Notebook $nb text="\tAllowed baseline drift - "+num2str(Baseline_Drift)+"%\r"	Notebook $nb text="\tAllowed maximal overlap - "+num2str(Overlap_Prc)+"%\r"	Notebook $nb text="\tThe remaining overlaps - "+Overlaps	Notebook $nb text="\r_______________________________________"		Notebook $nb text="\r\rFound "+num2str(Total_peaks_number)+" events\r"	Notebook $nb ruler=Normal; Notebook $nb  margins={0,0,720}, rulerDefaults={"Arial",7,1,(0,0,0)}, tabs={36,72,108,144,180,216,252,288,324,360,396,432,468,504,541,576}	Notebook $nb text=Stats_names	If(Total_peaks_number==0)		abort 	endif			String Waves_to_show="Interspike_interval,"+Values_to_show	Make/O/N=(Total_peaks_number-1) Interspike_interval	Variable i=0	do		If(i>0)			Interspike_interval[i-1]=(T_Max[i]-T_Max[i-1])*1000		endif		i+=1	while (i<Total_peaks_number)	Variable ii=0	String Mean_line	If(cmpstr(Population_Center, "Mean" )==0)		Mean_line="Mean\t"	else		Mean_line="Median\t"	endif			String SD_line="SD\t"	String SE_line="SE\t"	String N_line="N\t"	Stat_result=Mean_SD("Interspike_interval")	Mean_line+=StringFromList(0, Stat_result ,";")+"\t\t"	SD_line+=StringFromList(1, Stat_result ,";")+"\t\t"	SE_line+=StringFromList(2, Stat_result ,";")+"\t\t"	N_line+=StringFromList(3, Stat_result ,";")+"\t\t"	ii=2	Variable exclude	do		exclude=0		One_wave=StringFromList(ii, Waves_to_show ,",")		if( strlen(One_wave) == 0 )			break		endif		If((cmpstr(One_wave,"T_Max")==0)%|(cmpstr(One_wave,"T_Bkg1")==0))			exclude=1		else			If((cmpstr(One_wave,"T_Bkg2")==0)%|(cmpstr(One_wave,"Fall_fit")==0))				exclude=1			endif		endif		If(exclude==1)			Mean_line+="\t"			SD_line+="\t"			SE_line+="\t"			N_line+="\t"		else			Stat_result=Mean_SD(One_wave)			Mean_line+=StringFromList(0, Stat_result ,";")+"\t"			SD_line+=StringFromList(1, Stat_result ,";")+"\t"			SE_line+=StringFromList(2, Stat_result ,";")+"\t"			N_line+=StringFromList(3, Stat_result ,";")+"\t"		endif		ii+=1	while(ii)	InsertPoints 0,1, Interspike_interval	Notebook $nb text=Mean_line+"\r"	Notebook $nb text=SD_line+"\r"	Notebook $nb text=SE_line+"\r"	Notebook $nb text=N_line+"\r"	Notebook $nb text="\r"	Notebook $nb text=Stats_names	i=0	do		Row_to_Print=""		 ii=0		do			if(strlen(StringFromList(ii, Waves_to_show ,",")) == 0 )				break			endif						If(cmpstr(StringFromList(ii, Waves_to_show ,","),"Fall_fit")==0)				Wave/T Fall_fit=Fall_fit				Row_to_Print+=Fall_fit[i]+"\t"			else				One_wave="One_wave="+"num2str("+StringFromList(ii, Waves_to_show ,",")+"["+num2str(i)+"])"				execute/Z One_wave				Row_to_Print+=One_wave+"\t"			endif					ii+=1		while(ii)		Row_to_Print+="\r"		Notebook $nb text="\t"+Row_to_Print		i+=1	while (i<Total_peaks_number)		Notebook $nb selection={startOfFile, endOfFile}	return 0EndFunction/S Mean_SD(name)	String name	SetDataFolder $"root:Quanta"	SVAR Population_Center=Population_Center	String Stat_result	Duplicate/o $name, Stat_wave	Sort Stat_wave Stat_wave	Do		If (Stat_wave[0]<0.0000001)			// the smallest possible number to consider. Change if nassesary.			Deletepoints 0, 1, Stat_wave		else			break		endif	While (1)	If(numpnts(Stat_wave)<=1)		Stat_result=" ; ; ; ; ;"	else		wavestats/Q Stat_wave		If(cmpstr(Population_Center, "Mean" )==0)			Stat_result=num2str(V_avg)+";"		else			SetScale/P x 0,1,Stat_wave			Variable Median = Stat_wave((numpnts(Stat_wave)-1)/2)			Stat_result=num2str(Median)+";"		endif		Stat_result+=num2str(V_sdev)+";"+num2str(V_sdev/sqrt(V_npnts))+";"+num2str(V_npnts)	endif	return Stat_resultend//_________Detection Limits___________//	Returns 1 if a spike has invalide shape, and 2 if it does not pass a cutoff.Function Detection_limits(Peak_pnt)	Variable Peak_pnt	SetDataFolder $"root:Quanta"	NVAR Spike_Max_T05=Spike_Max_T05	NVAR Spike_Max_Trise=Spike_Max_Trise	NVAR Spike_Min_Imax=Spike_Min_Imax	Wave Peak_Q=Peak_Q	Wave Peak_Imax=Peak_Imax	Wave Peak_t05=Peak_t05	Wave Fall_slope=Fall_slope	Wave Rise_slope=Rise_slope	Wave Rise_time=Rise_time	If ((Peak_Q[Peak_pnt]<=0)%|(Peak_t05[Peak_pnt]<=0))		return 1	endif	If ((Fall_slope[Peak_pnt]<=0)%|(Rise_slope[Peak_pnt]<=0))		return 1	endif	If ((Spike_Max_Trise>0)&(Rise_time[Peak_pnt]>Spike_Max_Trise))		return 2	endif	If ((Spike_Max_T05>0)&(Peak_t05[Peak_pnt]>Spike_Max_T05))		return 2	endif	If (Peak_Imax[Peak_pnt]<Spike_Min_Imax)		return 2	endif	return 0EndFunction Detection_limits_Foot(Peak_pnt)	Variable Peak_pnt	SetDataFolder $"root:Quanta"	NVAR Foot_Min_W=Foot_Min_W	NVAR Foot_Min_H=Foot_Min_H	Wave Foot_Q=Foot_Q	Wave Foot_I=Foot_I	Wave Foot_W=Foot_W	If ((Foot_W[Peak_pnt]<=Foot_Min_W)%|(Foot_I[Peak_pnt]<=Foot_Min_H))		return 1	endif	if(Foot_Q[Peak_pnt]<=0)		return 1	endif	Wavestats/Q Foot_W	If(V_numNans!=0)		return 1	endif	return 0EndFunction Change_Detection_Limits(ctrlName,checked) : CheckBoxControl	String ctrlName	Variable checked	SetDataFolder $"root:Quanta"	NVAR Spike_Min_Imax=Spike_Min_Imax	NVAR Spike_Min_Imax_Last=Spike_Min_Imax_Last	NVAR Spike_Max_T05=Spike_Max_T05	NVAR Spike_Max_T05_Last=Spike_Max_T05_Last	NVAR Spike_Max_Trise=Spike_Max_Trise	NVAR Spike_Max_Trise_Last=Spike_Max_Trise_Last	NVAR Foot_Min_H=Foot_Min_H	NVAR Foot_Min_H_Last=Foot_Min_H_Last	strswitch(ctrlName)		case "Detection_Spike_Imax":			If(checked==1)				Spike_Min_Imax=Spike_Min_Imax_Last			else				Spike_Min_Imax_Last=Spike_Min_Imax				Spike_Min_Imax=0			endif			break		case "Detection_Spike_T05":			If(checked==1)				Spike_Max_T05=Spike_Max_T05_Last			else				Spike_Max_T05_Last=Spike_Max_T05				Spike_Max_T05=0			endif			break		case "Detection_Spike_Trise":			If(checked==1)				Spike_Max_Trise=Spike_Max_Trise_Last			else				Spike_Max_Trise_Last=Spike_Max_Trise				Spike_Max_Trise=0			endif			break		case "Detection_Foot_H":			If(checked==1)				Foot_Min_H=Foot_Min_H_Last			else				Foot_Min_H_Last=Foot_Min_H				Foot_Min_H=0			endif			break	endswitchEnd//__________________________________//___________Other  controls___________//__________________________________Function Foot_Min_W_chk(ctrlName,checked) : CheckBoxControl	String ctrlName	Variable checked	SetDataFolder $"root:Quanta"	NVAR Foot_Min_W=Foot_Min_W	NVAR Foot_Min_W_Last=Foot_Min_W_Last	NVAR SSFoot_Do=SSFoot_Do	NVAR Native_Foot_Del=Native_Foot_Del	strswitch (ctrlName)		case "Detection_Foot_W":			If(checked==1)				Foot_Min_W=Foot_Min_W_Last			else				CheckBox Detection_SSFoot_Chk,value= 0				Foot_Min_W_Last=Foot_Min_W				Foot_Min_W=0				SSFoot_Do=0			endif		break		case "Detection_SSFoot_Chk":			If(checked==1)				CheckBox Detection_Foot_W,value= 1				Foot_Min_W=Foot_Min_W_Last				SSFoot_Do=1			else				CheckBox Detection_Foot_W,value= 0				Foot_Min_W_Last=Foot_Min_W				Foot_Min_W=0				SSFoot_Do=0			endif		break		case "Detection_Native_Foot_Chk":			If(checked==1)				Native_Foot_Del=1			else				Native_Foot_Del=0			endif		break	endswitchEndFunction Check_the_Box(ctrlName,varNum,varStr,varName) : SetVariableControl	String ctrlName	Variable varNum	String varStr	String varName	NVAR Spike_Min_Imax=Spike_Min_Imax	NVAR Spike_Max_T05=Spike_Max_T05	NVAR Spike_Max_Trise=Spike_Max_Trise	NVAR Foot_Min_W=Foot_Min_W	NVAR Foot_Min_H=Foot_Min_H	NVAR Smooth_more=Smooth_more	NVAR Smooth_Derivative=Smooth_Derivative	strswitch (varName)		case "Smoothing_Factor_Add":			Smooth_more=1			CheckBox Scales_Smooth_Add,value=1			break		case "Smoothing_Factor_diff1":			Smooth_Derivative=1			CheckBox Scales_Smooth_Diff,value=1			break		case "Spike_Min_Imax":			If(Spike_Min_Imax>0)				CheckBox Detection_Spike_Imax,value=1			else				CheckBox Detection_Spike_Imax,value=0			endif			break		case "Spike_Max_T05":			If(Spike_Max_T05>0)				CheckBox Detection_Spike_t05,value=1			else				CheckBox Detection_Spike_t05,value=0			endif			break		case "Spike_Max_Trise":			If(Spike_Max_Trise>0)				CheckBox Detection_Spike_Trise,value=1			else				CheckBox Detection_Spike_Trise,value=0			endif			break		case "Foot_Min_H":			If(Foot_Min_H>0)				CheckBox Detection_Foot_H,value=1			else				CheckBox Detection_Foot_H,value=0			endif			break		case "Foot_Min_W":			If(Foot_Min_W>0)				CheckBox Detection_Foot_W,value=1			else				CheckBox Detection_Foot_W,value=0				CheckBox Detection_SSFoot_Chk,value= 0			endif			break	endswitchEndFunction Close_Options(ctrlName) : ButtonControl	String ctrlName	SetDataFolder $"root:Quanta"	String Win_name=WinName(0,64)	NVAR T_Start=T_Start	NVAR T_End=T_End	WAVE Working_trace_copy=Working_trace_copy	NVAR T_Delta=T_Delta	NVAR Gain=Gain	NVAR Gain_Temp=Gain_Temp	T_Start=pnt2x(Working_trace_copy,0)	T_End=pnt2x(Working_trace_copy,(numpnts(Working_trace_copy)-1))	T_Delta=(pnt2x(Working_trace_copy,1)-pnt2x(Working_trace_copy,0))*1000	Gain_Temp=Gain	Dowindow/K $Win_nameEndFunction Show_Extras(theTag) : ButtonControl	String theTag	SetDataFolder $"root:Quanta"	NVAR Peak_ID=Peak_ID	NVAR Show_Legend=Show_Legend	NVAR Total_peaks_number=Total_peaks_number	Variable Peak_pnt=Peak_ID-1	strswitch (theTag)		case "Zoom_Off":			Button Zoom_Off rename=Zoom_On, title="Hide Zoom"//			MoveWindow /W=Main_window 3,80,549,394.25			execute "Zoom_Win()"			AutoPositionWindow/E/M=0/R=Main_window Zoom_Win			SetVariable ID,limits={1,(Total_peaks_number),1},win=Zoom_Win			If(Peak_ID)				Draw_lines_zoom_window(Peak_pnt)			endif			Dowindow/F Zoom_Win			break		case "Zoom_On":			Button Zoom_On rename=Zoom_Off, title="Zoom Win"			Dowindow/K Zoom_Win//			MoveWindow /W=Main_window 3,80,762,394.25			break		case "Legend_Off":			Show_Legend=1			Button Legend_Off rename=Legend_On, title="Hide Legend"			Generate_annotation(Peak_pnt)			break		case "Legend_On":			Show_Legend=0			TextBox/W=Main_window/N=Peak_data/K			Button Legend_On rename=Legend_Off, title="Show Legend"			break	endswitchEndFunction Show_options_panel(ctrlName) : ButtonControl	String ctrlName	if (CheckName("Options_Tab_Panels", 6)==0)		execute "Options_Tab_Panels(0)"	else		Dowindow/F Options_Tab_Panels	endif	strswitch (ctrlName)		case "Menu_FiltersScales":			Redraw_Tabs(ctrlName,0)			break		case "Menu_Cutoffs":			TabControl Tab_thing, value= 1			Redraw_Tabs(ctrlName,1)			break		case "Menu_Results":			TabControl Tab_thing, value= 2			Redraw_Tabs(ctrlName,2)			break		case "Menu_Stats":			TabControl Tab_thing, value= 3			Redraw_Tabs(ctrlName,3)			break	endswitchEndFunction Redraw_Tabs(name,tabNumber)	String name	Variable tabNumber	SetDataFolder $"root:Quanta"	String Existing_controls=ControlNameList("")	NVAR T_Start=T_Start	NVAR T_End=T_End	WAVE Working_trace_copy=Working_trace_copy	NVAR T_Delta=T_Delta	NVAR Gain=Gain	NVAR Gain_Temp=Gain_Temp	T_Start=pnt2x(Working_trace_copy,0)	T_End=pnt2x(Working_trace_copy,(numpnts(Working_trace_copy)-1))	T_Delta=(pnt2x(Working_trace_copy,1)-pnt2x(Working_trace_copy,0))*1000	Gain_Temp=Gain		Options_Tab_Panels_controls(tabNumber)	Button Recalculate_btn,win=Options_Tab_Panels, fColor=(0,0,0)	If(tabNumber==0)		NVAR Smooth_more=Smooth_more		If(Smooth_more==1)			CheckBox Scales_Smooth_Add,value=1		endif		NVAR Smooth_Derivative=Smooth_Derivative		If(Smooth_Derivative==1)			CheckBox Scales_Smooth_Diff,value=1		endif	endif	If(tabNumber==1)		NVAR Spike_Min_Imax=Spike_Min_Imax		NVAR Spike_Max_T05=Spike_Max_T05		NVAR Spike_Max_Trise=Spike_Max_Trise		NVAR Foot_Min_W=Foot_Min_W		NVAR SSFoot_Do=SSFoot_Do		NVAR Native_Foot_Del=Native_Foot_Del		NVAR Foot_Min_H=Foot_Min_H		SVAR Overlaps=Overlaps		If(Spike_Min_Imax>0)			CheckBox Detection_Spike_Imax,value=1		endif		If(Spike_Max_T05>0)			CheckBox Detection_Spike_t05,value=1		endif		If(Spike_Max_Trise>0)			CheckBox Detection_Spike_Trise,value=1		endif		If(Foot_Min_H>0)			CheckBox Detection_Foot_H,value=1		endif		If(Foot_Min_W>0)			CheckBox Detection_Foot_W,value=1		endif		If(SSFoot_Do==1)			CheckBox Detection_SSFoot_Chk,value=1		endif		If(Native_Foot_Del==1)			CheckBox Detection_Native_Foot_Chk,value=1		endif				If(cmpstr(Overlaps,"Ignore")==0)			PopupMenu Detection_Overlaps,mode=1, win=Options_Tab_Panels		else			If(cmpstr(Overlaps,"Separate")==0)				PopupMenu Detection_Overlaps,mode=2, win=Options_Tab_Panels			else				PopupMenu Detection_Overlaps,mode=3, win=Options_Tab_Panels			endif		endif	endif		If(tabNumber==2)		NVAR Show_Time=Show_Time		NVAR Show_Base=Show_Base		NVAR Show_Width=Show_Width		NVAR Show_H=Show_H		NVAR Show_Q=Show_Q		NVAR Show_Molec=Show_Molec		NVAR Show_Rise_t=Show_Rise_t		NVAR Show_Rise_r=Show_Rise_r		NVAR Show_Fall_t=Show_Fall_t		NVAR Show_Fall_r=Show_Fall_r		NVAR Show_Ft_H=Show_Ft_H		NVAR Show_Ft_width=Show_Ft_width		NVAR Show_Ft_Q=Show_Ft_Q		NVAR Show_Ft_molec=Show_Ft_molec		SVAR Fit_method=Fit_method			SetDrawEnv fname= "Arial"		DrawText 115,191,"%"		If(Show_Time==1)			CheckBox Results_Show1,value=1		endif		If(Show_Base==1)			CheckBox Results_Show2,value=1		endif		If(Show_Width==1)			CheckBox Results_Show4,value=1		endif		If(Show_H==1)			CheckBox Results_Show5,value=1		endif		If(Show_Q==1)			CheckBox Results_Show6,value=1		endif		If(Show_Molec==1)		CheckBox Results_Show7,value=1			endif		If(Show_Rise_t==1)			CheckBox Results_Show8,value=1		endif		If(Show_Rise_r==1)			CheckBox Results_Show9,value=1		endif		If(Show_Fall_t==1)			CheckBox Results_Show10,value=1		endif		If(Show_Fall_r==1)			CheckBox Results_Show11,value=1		endif		If(Show_Ft_H==1)			CheckBox Results_Show12,value=1		endif		If(Show_Ft_width==1)			CheckBox Results_Show13,value=1		endif		If(Show_Ft_Q==1)			CheckBox Results_Show14,value=1		endif		If(Show_Ft_molec==1)			CheckBox Results_Show15,value=1		endif		If(cmpstr(Fit_method,"Line")==0)			PopupMenu Results_Fallfit,mode=1,popvalue="Line", win=Options_Tab_Panels		else			If(cmpstr(Fit_method,"Exp")==0)				PopupMenu Results_Fallfit,mode=2,popvalue="Exp", win=Options_Tab_Panels			else				PopupMenu Results_Fallfit,mode=3,popvalue="DblExp", win=Options_Tab_Panels				SetVariable Results_Fall_Chi, win=Options_Tab_Panels, disable=0			endif		endif	endif	If(tabNumber==3)		SVAR Population_Center=Population_Center		SVAR Norm_point=Norm_point		If(cmpstr(Population_Center,"Mean")==0)			PopupMenu Stats_Population,mode=2,popvalue="Mean", win=Options_Tab_Panels		endif		If(cmpstr(Norm_point,"Rise")==0)			PopupMenu Stats_AvePeak,mode=2,popvalue="Rise", win=Options_Tab_Panels		endif	endifendFunction Overlaps_PopMenu(theTag,popNum,popStr) : PopupMenuControl	String theTag	Variable popNum	String popStr	SetDataFolder $"root:Quanta"	SVAR Overlaps=Overlaps	Overlaps=popStr	Redraw_Tabs(theTag,1)EndFunction Scales_Switch(ctrlName,checked) : CheckBoxControl	String ctrlName	Variable checked	SetDataFolder $"root:Quanta"	Variable Radio_On=1	strswitch (ctrlName)		case "Scales_Change_Delta":			Radio_On= 1			SetVariable Scales_End_time,disable=2			SetVariable Scales_Delta_Time,disable=0			break		case "Scales_Change_End":			Radio_On= 2			SetVariable Scales_End_time,disable=0			SetVariable Scales_Delta_Time,disable=2			break	endswitch	CheckBox Scales_Change_Delta,mode=1, value= Radio_On==1	CheckBox Scales_Change_End,mode=1, value= Radio_On==2EndWindow Options_Tab_Panels(Tab) : Panel	Variable Tab	PauseUpdate; Silent 1	NewPanel /K=1 /W=(235,104,506,401) as "Detection Options"	TabControl Tab_thing,pos={6,6},size={260,261},proc=Redraw_Tabs	TabControl Tab_thing,tabLabel(0)="Filters/Scales",tabLabel(1)="Cutoffs",tabLabel(2)="Results",tabLabel(3)="Stats"	TabControl Tab_thing,value= tabEndFunction Options_Tab_Panels_controls(Tab)	Variable Tab	SetDrawLayer/K UserBack	NVAR T_Delta=T_Delta	Button Close_window,pos={65,273},size={70,20},proc=Close_Options,title="Close"	Button Recalculate_btn,pos={138,273},size={70,20},proc=Recalculate_Peaks_Btn,title="Recalc"		Variable Min_Freq=Binomial_to_Gaussian_Calc(32767)	GroupBox Scales_Smooth_Box,pos={18,27},size={235,85},fColor=(26112,0,10240), title="Filters",disable= (tab!=0)	Button Scales_Estimate_Filters,pos={80,46},size={109,18},proc=Filter_estimate,title="Estimate Filters",disable=(tab!=0)	Button Scales_Estimate_Filters,help={"Estimates the corner frequencies of the three filters used during trace preconditioning."}	CheckBox Scales_Smooth_Add,pos={25,70},size={124,14},proc=Additional_Filters,title="Filter trace additionally"	CheckBox Scales_Smooth_Add,value= 0,disable= (tab!=0)	CheckBox Scales_Smooth_Add,help={"Additional trace filtering (Gaussian). Increases signal-to-noise, but does not affect spike parameters."}	CheckBox Scales_Smooth_Diff,pos={25,90},size={124,14},proc=Additional_Filters,title="Filter the 1st derivative"	CheckBox Scales_Smooth_Diff,value= 0,disable= (tab!=0)	CheckBox Scales_Smooth_Diff,help={"Gaussian filtering of differentiated trace. Increases signal-to-noise, but does not affect spike parameters."}	SetVariable Scales_Smooth_F,pos={156,70},size={84,16},title=" ",format="%.1W1PHz", proc=Check_the_Box,disable= (tab!=0)	SetVariable Scales_Smooth_F,limits={Min_Freq+1,(1/(T_Delta/1000))/2,50},value= root:Quanta:Smoothing_Factor_Add	SetVariable Scales_Diff_smooth,pos={156,90},size={84,16},title=" ",format="%.1W1PHz", proc=Check_the_Box,disable= (tab!=0)	SetVariable Scales_Diff_smooth,limits={Min_Freq+1,(1/(T_Delta/1000))/2,50},value= root:Quanta:Smoothing_Factor_diff1	GroupBox Scales_Y_Box,pos={18,116},size={235,40},title="Y scale (pA)",fColor=(26112,0,10240),disable= (tab!=0)	SetVariable Scales_Mult,pos={47,134},size={193,16},title="Multiply the current by:",disable= (tab!=0)	SetVariable Scales_Mult,fSize=10,limits={0,1e+18,10},value= root:Quanta:Gain_Temp,proc=Adjust_Trace_Scales	SetVariable Scales_Mult,help={"Gain. The current has to be in pA!"}	GroupBox Scales_X_Box,pos={18,160},size={235,98}, title="X scale (seconds)",disable= (tab!=0),fColor=(26112,0,10240),disable= (tab!=0)	SetVariable Scales_Start_time,pos={54,181},size={136,16},title="Start (s)",fSize=10,disable= (tab!=0)	SetVariable Scales_Start_time,limits={0,Inf,1},value= root:Quanta:T_Start,proc=Adjust_Trace_Scales	SetVariable Scales_Start_time,help={"Set the time of the first datapoint (seconds)."}	SetVariable Scales_End_time,pos={57,203},size={133,16},fSize=10,title="End (s)",disable= (tab!=0)	SetVariable Scales_End_time,help={"Set the time of the last datapoint (seconds)."}	SetVariable Scales_End_time,limits={0,Inf,1},value= root:Quanta:T_End,proc=Adjust_Trace_Scales	SetVariable Scales_Delta_Time,pos={43,224},size={147,16},fSize=10,fstyle=0, title="Delta (ms)",disable= (tab!=0),format="%g"	SetVariable Scales_Delta_Time,limits={0.001,Inf,0.01},value= root:Quanta:T_Delta,proc=Adjust_Trace_Scales	SetVariable Scales_Delta_Time,help={"Set the sampling interval (milli-seconds)."}	ValDisplay Scales_Delta_Hz,pos={80,240},size={74,16},fSize=10,fstyle=0,format="%.1W1PHz",mode=2	ValDisplay Scales_Delta_Hz,limits={0,0,0},barmisc={0,1000},disable= (tab!=0)	ValDisplay Scales_Delta_Hz,help={"Sampling frequency."},value= #"1/(root:Quanta:T_Delta/1000)"	CheckBox Scales_Change_End,pos={198,205},size={109,16},proc=Scales_Switch,title=" ",mode=1,value= 0,disable= (tab!=0)	CheckBox Scales_Change_Delta,pos={198,226},size={103,16},proc=Scales_Switch,title=" ",mode=1,value= 1,disable= (tab!=0)	If(tab==0)		SetVariable Scales_End_Time,disable=2		Button Recalculate_btn,proc=Change_trace_scales,title="Change"	endif	GroupBox Detection_Box_Spike,pos={18,27},size={235,77},fColor=(26112,0,10240), title="Spike cutoffs",disable= (tab!=1)	CheckBox Detection_Spike_Imax,pos={31,45},size={97,14},proc=Change_Detection_Limits,title="Min Spike I(max), (pA)"	CheckBox Detection_Spike_Imax,value= 0,disable= (tab!=1)	SetVariable Detection_Set_Spike_Imax,pos={163,45},size={75,16},title=" ", proc=Check_the_Box	SetVariable Detection_Set_Spike_Imax,limits={0,Inf,1},value= root:Quanta:Spike_Min_Imax,disable= (tab!=1)	CheckBox Detection_Spike_t05,pos={31,64},size={100,14},proc=Change_Detection_Limits,title="Max Spike T(1/2), (ms)"	CheckBox Detection_Spike_t05,value= 0,disable= (tab!=1)	SetVariable Detection_Set_Spike_t05,pos={163,64},size={75,16},title=" ", proc=Check_the_Box	SetVariable Detection_Set_Spike_t05,limits={0,Inf,0.5},value= root:Quanta:Spike_Max_T05,disable= (tab!=1)	CheckBox Detection_Spike_trise,pos={31,83},size={100,14},proc=Change_Detection_Limits,title="Max Spike T(rise), (ms)"	CheckBox Detection_Spike_trise,value= 0,disable= (tab!=1)	SetVariable Detection_Set_Spike_trise,pos={163,83},size={75,16},title=" ", proc=Check_the_Box	SetVariable Detection_Set_Spike_trise,limits={0,Inf,0.5},value= root:Quanta:Spike_Max_Trise,disable= (tab!=1)	GroupBox Detection_Box_Foot,pos={18,106},size={235,73},fColor=(26112,0,10240), title="Foot cutoffs",disable= (tab!=1)	CheckBox Detection_Foot_H,pos={31,122},size={90,14},proc=Change_Detection_Limits,title="Min Foot Height (pA)"	CheckBox Detection_Foot_H,value= 0,disable= (tab!=1)	SetVariable Detection_Set_Min_Foot_H,pos={163,123},size={75,16},title=" ", proc=Check_the_Box	SetVariable Detection_Set_Min_Foot_H,limits={0,Inf,1},value= root:Quanta:Foot_Min_H,disable= (tab!=1)	CheckBox Detection_Foot_W,pos={31,141},size={96,14},proc=Foot_Min_W_chk,title="Min Foot Width (ms)"	CheckBox Detection_Foot_W,value= 0,disable= (tab!=1)	SetVariable Detection_Set_Min_Foot_W,pos={163,141},size={75,16},title=" ", proc=Check_the_Box	SetVariable Detection_Set_Min_Foot_W,limits={0,Inf,0.5},value= root:Quanta:Foot_Min_W,disable= (tab!=1)	CheckBox Detection_SSFoot_Chk,pos={31,160},size={96,14},proc=Foot_Min_W_chk,title="Steady-state feet only"	CheckBox Detection_SSFoot_Chk,value= 0,disable= (tab!=1)	CheckBox Detection_SSFoot_Chk, help={"Only feet with steady-states that persist for 'Min Foot Width' duration will be considered."}	CheckBox Detection_Native_Foot_Chk,pos={162,160},size={96,14},proc=Foot_Min_W_chk,title="Delete 'native'"	CheckBox Detection_Native_Foot_Chk,value= 0,disable= (tab!=1)	CheckBox Detection_Native_Foot_Chk, help={"'Native' foot width=0.33*(50-90%)risetime. (Chow, 95)."}	GroupBox Detection_Bad_Peaks,pos={18,180},size={235,78},fColor=(26112,0,10240), title="'Bad' spikes",disable= (tab!=1)	SetVariable Detection_Baseline_Drift,pos={71,197},size={167,18},title="Allowed Baseline Drift",format="%.0W0P%"	SetVariable Detection_Baseline_Drift,limits={1,100,5},value= root:Quanta:Baseline_Drift,disable= (tab!=1)	SetVariable Detection_Baseline_Drift, help={"Baseline drift is the delta current between spike beginning and end."}	SetVariable Detection_Overlap_Prc,pos={33,216},size={205,16},title="Delete spike overlapping by >",format="%.0W0P%"	SetVariable Detection_Overlap_Prc,limits={0,100,5},value= root:Quanta:Overlap_Prc,disable= (tab!=1)	SetVariable Detection_Overlap_Prc, help={"% overlap is the ratio of the current at the minimum between two spikes and the I(max) of the smaller spike."}	PopupMenu Detection_Overlaps,pos={57,234},proc=Overlaps_PopMenu,title="Remaining Overlaps:",disable= (tab!=1)	PopupMenu Detection_Overlaps,mode=1,popvalue="Ignore",value= #"\"Ignore;Separate;Split\""	PopupMenu Detection_Overlaps help={"Spikes overlap if the duration between the end of the 1st and the beginning of the 2nd spike is less then 2*t1/2."}	GroupBox Results_Peak_Box, pos={18,27},size={115,231},fColor=(26112,0,10240), title="Spike Param",disable= (tab!=2)	CheckBox Results_show1,pos={25,45},size={150,14},title="T(max), (s)",value= 0,proc=Change_Table,disable= (tab!=2)	CheckBox Results_show1 help={"Time (seconds) at spike Maximum."}	CheckBox Results_show2,pos={25,64},size={150,14},title="T(base), (ms)",value= 0,proc=Change_Table,disable= (tab!=2)	CheckBox Results_show2 help={"Duration from the Start to the End of a spike."}	CheckBox Results_show4,pos={25,83},size={150,14},title="T(1/2), (ms)",value= 0,proc=Change_Table,disable= (tab!=2)	CheckBox Results_show4 help={"Spike width (ms) at 50% of its height."}	CheckBox Results_show5,pos={25,102},size={150,14},title="I(max), (pA)",value= 0,proc=Change_Table,disable= (tab!=2)	CheckBox Results_show5 help={"Spike height (pA) from its maximum to the background under the maximum."}	CheckBox Results_show6,pos={25,120},size={150,14},title="Q (pC)",value= 0,proc=Change_Table,disable= (tab!=2)	CheckBox Results_show6 help={"Spike charge (pA/s)."}	CheckBox Results_show7,pos={25,140},size={150,14},title="Q (molecules)",value= 0,proc=Change_Table,disable= (tab!=2)	CheckBox Results_show7 help={"Peak charge (pA/s) multiplied by 3.121*10^6."}	CheckBox Results_show8,pos={25,159},size={150,14},title="T(rise), (ms)",value= 0,proc=Change_Table,disable= (tab!=2)	CheckBox Results_show8 help={"Time between 2 points on spike rising phase. Foot is excluded!"}	SetVariable Results_Rise_Low,pos={26,177},size={36,20},title=" ",limits={0,50,5},proc=Change_Table_RiseTime,value=root:Quanta:Rise_Low_Prc,frame=1,disable= (tab!=2)	SetVariable Results_Rise_Low, help={"Lower point (excluding the foot). Calculated using rise phase linear extrapolation. 0-50% of Imax."}	SetVariable Results_Rise_Hi,pos={65,177},size={48,20},title="to",limits={55,100,5},proc=Change_Table_RiseTime,value=root:Quanta:Rise_Hi_Prc,frame=1,disable= (tab!=2)	SetVariable Results_Rise_Hi, help={"Upper point : 55-100% of Imax."}	CheckBox Results_show9,pos={25,198},size={150,14},title="Rise (pA/ms)",value= 0,proc=Change_Table,disable= (tab!=2)	CheckBox Results_show9 help={"Linear slope of spike rising phase. The middle of the slope is found at dI/dt maximum"}	CheckBox Results_show10,pos={25,217},size={150,14},title="Fall time (ms)",value= 0,proc=Change_Table,disable= (tab!=2)	CheckBox Results_show10 help={"Time between 75 and 25% of the Imax on peak falling phase."}	CheckBox Results_show11,pos={25,236},size={150,14},title="Fall (...ms)",value= 0,proc=Change_Table,disable= (tab!=2)	CheckBox Results_show11 help={"Linear slope (pA/ms) between 75 and 25% of the Imax or tau (ms) of the exp fit between 75% and the End of the peak."}	GroupBox Results_Foot_Box, pos={138,27},size={115,98},fColor=(26112,0,10240), title="Foot Param",disable= (tab!=2)	CheckBox Results_show12,pos={148,45},size={150,14},title="I(foot), (pA)",value= 0,proc=Change_Table,disable= (tab!=2)	CheckBox Results_show12 help={"Foot current"}	CheckBox Results_show13,pos={148,64},size={150,14},title="T(foot), (ms)",value= 0,proc=Change_Table,disable= (tab!=2)	CheckBox Results_show13 help={"Foot duration. Time from the spike start to foot end."}	CheckBox Results_show14,pos={148,83},size={150,14},title="Q(foot), (pC)",value= 0,proc=Change_Table,disable= (tab!=2)	CheckBox Results_show14 help={"Foot area (pC)."}	CheckBox Results_show15,pos={148,102},size={150,14},title="Q(foot), (molec)",value= 0,proc=Change_Table,disable= (tab!=2)	CheckBox Results_show15 help={"Foot area multiplied by 3.121*10^6 (molecules/pC)."}	GroupBox Results_FallFit_Box, pos={138,130},size={115,70},fColor=(26112,0,10240), title="Spike fall",disable= (tab!=2)	PopupMenu Results_Fallfit,pos={150,150},size={103,21},title="Fit",disable= (tab!=2)	PopupMenu Results_Fallfit,mode=3,popvalue="DblExp",value= #"\"Line;Exp;DblExp\"",proc=Fall_fit_PopMenu	PopupMenu Results_Fallfit, help={"Choose the function to fit spike falling phase."}	SetVariable Results_Fall_Chi, pos={150,177},size={86,16},title="Chi2 ratio",limits={0,1e+03,1},value= Fall_ChiRatio_Cutoff,disable=1	SetVariable Results_Fall_Chi, help={"DblExp fit will be used only if Chi2s ratio from Exp to DblExp fits > that this threshold. Set to zero to force DblExp!"}	GroupBox Stats_Stat_Box, pos={18,50},size={235,55},fColor=(26112,0,10240), title="Statistics",disable= (tab!=3)	PopupMenu Stats_Population,pos={28,74},size={103,21},title="Stats are calculated using the",disable= (tab!=3)	PopupMenu Stats_Population,mode=1,popvalue="Median",value= #"\"Median;Mean\"",proc=Stats_PopMenu	PopupMenu Stats_Population, help={"Choose 'mean' for normally distributed data. Use 'Stats' button on the Main window."}	GroupBox Stats_AvePeak_Box, pos={18,120},size={235,55},fColor=(26112,0,10240), title="Average Spike",disable= (tab!=3)	PopupMenu Stats_AvePeak,pos={67,143},size={103,21},title="Line up the spikes by",disable= (tab!=3)	PopupMenu Stats_AvePeak,mode=1,popvalue="Max",value= #"\"Max;Rise\"",proc=Stats_PopMenu	PopupMenu Stats_AvePeak, help={"The point by which the spikes will be lined up during averaging. Use 'Avg peak' button on the Main window."}Endmacro Window Main_window() : Graph	PauseUpdate; Silent 1	String fldrSav= GetDataFolder(1)	SetDataFolder root:Quanta:	Display /W=(3,80,549,394.25) Working_trace_copy	SetDataFolder fldrSav	ModifyGraph margin(left)=45,gfSize=8,cbRGB=(48896,65280,48896)	ModifyGraph lblLatPos(left)=-80,lblLatPos(bottom)=150	Cursor/P A Working_trace_copy 0;Cursor/P B Working_trace_copy 0	ControlBar 70	GroupBox Left_Axis_Controls,pos={1,178},size={22,103},labelBack=(48896,65280,48896)	Button Zoom_V_in,pos={4,212},size={15,15},proc=Zoom_Up_Q,title="+"	Button Zoom_V_out,pos={4,235},size={15,15},proc=Zoom_Down_Q,title="-"	Button Move_V_up,pos={4,183},size={15,15},proc=Move_Up_Q,title="A"	Button Move_V_down,pos={4,261},size={15,15},proc=Move_Down_Q,title="V"	GroupBox Bottom_Axis_Controls,pos={381,69},size={102,22},labelBack=(48896,65280,48896)	Button Move_H_L,pos={385,73},size={15,15},proc=Move_Left_Q,title="<"	Button Zoom_H_out,pos={413,73},size={15,15},proc=Zoom_OutHoriz_Q,title="-"	Button Zoom_H_in,pos={436,73},size={15,15},proc=Zoom_In_Horiz_Q,title="+"	Button Move_H_R,pos={464,73},size={15,15},proc=Move_Right_Q,title=">"	Variable Min_Freq=Binomial_to_Gaussian_Calc(32767)	GroupBox Separator1,pos={1,0},size={108,69}	Button Smooth,pos={58,45},size={48,21},proc=SmoothBtn_Q,title="Smooth"	PopupMenu Smoth_meth,pos={4,3},size={103,21},proc=Smooth_method_Q	PopupMenu Smoth_meth,mode=3,popvalue="Binomial sm. ",value= #"\"LP Gaussian ;HP Gaussian ;Binomial sm. ;Boxcar sm.   ;Sav.-Gol. sm.\""	SetVariable Smooth_F,pos={5,27},size={100,16},title="Hz"	SetVariable Smooth_F,limits={Min_Freq+1,(1/(T_Delta/1000))/2,50},value= root:Quanta:Smoothing_Factor	SetVariable Smooth_F,help={"-3dB Cutoff frequency"}	Button Show_orig,pos={3,45},size={54,21},proc=Show_original_rec,title="Show Orig"	GroupBox Overall_smooth,pos={0,69},size={109,22},labelBack=(48896,65280,48896)	ValDisplay Display_Overall_Filter,pos={3,72},size={100,15}, value=root:Quanta:Overall_Filter,title="Final fc:",mode=1	ValDisplay Display_Overall_Filter,format="%.1W1PHz",fsize=9,labelBack=(48896,65280,48896)	ValDisplay Display_Overall_Filter,help={"Overall cutoff frequency of several Gaussian filters in a series. Does not work for boxcar and polynomial smoothings."}	GroupBox Separator201,pos={111,1},size={145,68}	GroupBox Bkg_HiLt,pos={114,3},size={37,25},labelBack=(39168,0,0),frame=0,disable=0	Button Bkg,pos={117,5},size={32,22},proc=Bkg_noise_Set,title="Bkg"	Button Bkg,help={"Position cursor A (round) at the beginning and cursor B (square) at the end of the trace's part, which does not have any spikes."}	Button Find_Pk,pos={153,5},size={98,22},proc=Peak_finder,title="Analyse Trace"	SetVariable Set_threshold,pos={118,30},size={133,16},title="Peak threshold=",help={"A spike is detected if dI/dt>=SD(dI)*threshold"}	SetVariable Set_threshold,limits={-Inf,Inf,0.5},value= root:Quanta:Detection_Mult	SetVariable Foot_threshold,pos={118,48},size={133,16},title="Foot threshold =",help={"Foot is detected if I(foot) >SD(I)*threshold"}	SetVariable Foot_threshold,limits={-Inf,Inf,0.5},value= root:Quanta:Detection_Foot_Mult	GroupBox Separator2,pos={258,1},size={147,53}	ValDisplay No_found,pos={338,6},size={59,15},title="of"	ValDisplay No_found,limits={0,0,0},barmisc={0,1000}	ValDisplay No_found,value= #"root:Quanta:Total_peaks_number"	SetVariable ID,pos={265,6},size={67,16},proc=Peak_locator_ID,title="ID"	SetVariable ID,limits={1,0,1},value= root:Quanta:Peak_ID	Button See_all,pos={298,26},size={65,22},proc=Zoom_Trace_In_Out,title="Zoom In"	Button Last,pos={264,26},size={33,22},proc=Peak_surf,title="<<"	Button Next,pos={364,26},size={33,22},proc=Peak_surf,title=">>"	GroupBox Separator3,pos={408,1},size={228,46},labelBack=(39168,0,0),frame=0	Button Del_All,pos={411,3},size={80,20},proc=Delete_all_peaks,title="Delete All"	Button Del_All,help={"Delete all spikes"}	Button Del,pos={411,25},size={80,20},proc=Delete_peak,title="Del Peak"	Button Del,help={"Currently selected spike will be deleted. If this peak is a part of splitted spikes, it will be added to the peak on its left."}	Button Spllit,pos={492,3},size={80,20},proc=Split,title="Split"	Button Spllit,help={"Use cursor A (round) to mark the split point."}	Button New_pk,pos={492,25},size={80,20},proc=Add_Peak_Manually,title="Add Peak"	Button New_pk,help={"Cursor A (round) should be at the beginning and cursor B (square) at the end of the spike."}	Button NewBase,pos={574,25},size={60,20},proc=New_Baseline,title="New Base"	Button NewBase,help={"Cursor A (round) should be at the beginning and cursor B (square) at the end of a spike."}	Button NewMax,pos={574,3},size={60,20},proc=New_T_Max,title="New Max"	Button NewMax,help={"Use cursor A (round) to mark a new spike Maximum."}	GroupBox Separator4,pos={638,0},size={86,49},labelBack=(48896,65280,65280)	Button Statistics,pos={641,3},size={80,24},proc=Show_stats,title="Stats"	Button Statistics,help={"Calculates Means, SD and SE for all parameters"}	Button Avg_Peak,pos={641,28},size={80,18},proc=Average_peaks,title="Avg peak"	Button Avg_Peak,help={"Averages the detected spikes"}	Slider X_Slider,pos={258,55},size={349,13},proc=Slider_Horiz_Q	Slider X_Slider,limits={0,1,0},value= 0.654716981132076,side= 0,vert= 0,ticks= 0	Button Reset_Slider_Btn,pos={610,50},size={25,18},proc=Slider_Reset,title="res"	Button Reset_Slider_Btn,help={"If slider does not work push here!"}	Button Save_zoom,pos={641,50},size={80,18},proc=Save_zoomed_trace,title="Save Zoom"	Button Save_zoom,help={"Saves recording in the Main window. Don't forget to include some area containing noise for future analysis."}EndMacro//___Zoom window controls____Window Zoom_Win() : Graph	PauseUpdate; Silent 1	SetDataFolder root:Quanta:	Variable Resolution=ScreenResolution	Variable Zoom_Win_width=207*96/Resolution	Display/K=1 /W=(555,80,555+Zoom_Win_width,299.75) root:Quanta:Zoomed_peak,root:Quanta:Fall_phase,root:Quanta:Rise_phase	ModifyGraph rgb(Zoomed_peak)=(0,0,0),rgb(Fall_phase)=(65280,0,0),rgb(Rise_phase)=(65280,0,0)	ModifyGraph gfSize=7, axOffset(left)=-3, lblLatPos(left)=-90, lblRot(left)=-90	Label left "\\f01\\Z07pA"	Label bottom "\\f01\\Z07s"	SetAxis/A	Cursor/P A Zoomed_peak 0;Cursor/P B Zoomed_peak 0	ControlBar 41	GroupBox Separator2,pos={1,0},size={77,40}	SetVariable ID,pos={6,3},size={67,16},proc=Peak_locator_ID,title="ID"	SetVariable ID,limits={1,0,1},value= root:Quanta:Peak_ID	Button Last,pos={6,20},size={33,18},proc=Peak_surf,title="<<"	Button Next,pos={41,20},size={33,18},proc=Peak_surf,title=">>"	CheckBox Zoom_to_Foot,pos={80,0},size={39,14},proc=Zoom_to_foot_proc,title="Foot"	CheckBox Zoom_to_Rise,pos={142,0},size={39,14},proc=Zoom_to_foot_proc,title="Rise"	CheckBox Zoom_to_Fall,pos={204,0},size={34,14},proc=Zoom_to_foot_proc,title="Fall"	GroupBox Separ_WholePeak,pos={79,14},size={189,25},labelBack=(39168,0,0),frame=0	Button Del,pos={82,17},size={60,20},proc=Delete_peak,title="Del Peak", disable=0	Button Del,help={"Currently selected spike will be deleted. If this peak is a part of splitted spikes, it will be added to the peak on its left."}	Button NewBase,pos={144,17},size={60,20},proc=New_Baseline,title="New Base", disable=0	Button NewBase,help={"Cursor A (round) should be at the beginning and cursor B (square) at the end of a Peak."}	Button NewMax,pos={206,17},size={60,20},proc=New_T_Max,title="New Max", disable=0	Button NewMax,help={"Use cursor A (round) to mark a new spike Maximum."}	GroupBox Separator3,pos={79,41},size={189,25},labelBack=(39168,0,0),frame=0,disable=1	Button FootDelete,pos={82,44},size={60,18},proc=Delete_Foot,title="Del Foot",disable=1	Button FootDelete,help={"Will delete the Foot of currently selected Peak."}	Button FootNew_H,pos={144,44},size={60,18},proc=New_Foot_I,title="Foot H",disable=1	Button FootNew_H,help={"Use cursor A (round) to set new Foot current."}	Button New_Rise,pos={144,44},size={60,18},proc=New_Rise,title="New Rise",disable=1	Button New_Rise,help={"Cursor A (round) should be on the lower and cursor B (square) on the higher part of peak's rise."}	PopupMenu Fall_fit_change,pos={173,43},size={103,21},title="Fit",disable=1	PopupMenu Fall_fit_change,mode=3,popvalue=root:Quanta:Fit_method,value= #"\"Line;Exp;DblExp\"",proc=Fall_fit_PopMenu_Single	PopupMenu Fall_fit_change, help={"Choose the function to fit the falling phase of the selected spike"}	PopupMenu Fall_Extrap_change,pos={131,78},size={103,21},title="Extrapolate",disable=1	PopupMenu Fall_Extrap_change,mode=2,popvalue=root:Quanta:Fit_method,value= #"\"Line;Exp\"",proc=Fall_Extrap_PopMenu_Single	PopupMenu Fall_Extrap_change, help={"Choose the function to extrapolate the falling phase of selected peak."}	SetVariable Extrap_Tau_Set title="Extrap Tau",pos={133,104},size={110,16},proc=Extrap_Tau_SetVar, disable=1	SetVariable Extrap_Tau_Set value=Fall_Tau_Extrap, format="%.2f",labelBack=(65535,65535,65535)EndMacroFunction Extrap_Tau_SetVar(ctrlName,varNum,varStr,varName) : SetVariableControl	String ctrlName	Variable varNum	String varStr	String varName	SetDataFolder root:Quanta:	Wave/T Fall_fit_Extrap=Fall_fit_Extrap	NVAR Peak_ID=Peak_ID	Fall_fit_Extrap[Peak_ID-1]=ReplaceStringByKey("Tau", Fall_fit_Extrap[Peak_ID-1], varStr)	String First_and_Last=Check_for_Separated_peaks(Peak_ID-1)	Variable First_separated_pnt=str2num(StringFromList(0,First_and_Last))	Variable Last_separated_pnt=str2num(StringFromList(1,First_and_Last))	Calc_Separated_peak_param(First_separated_pnt,Last_separated_pnt)	Draw_Lines_All(Peak_ID-1)EndFunction Zoom_to_Foot_Proc (theTag,checked) : CheckBoxControl	String theTag	Variable checked	NVAR Peak_ID=Peak_ID	NVAR Total_peaks_number=Total_peaks_number		strswitch (theTag)		case "Zoom_to_Foot":			CheckBox Zoom_to_Rise value=0			CheckBox Zoom_to_Fall value=0			break		case "Zoom_to_Rise":			CheckBox Zoom_to_Foot value=0			CheckBox Zoom_to_Fall value=0			break		case "Zoom_to_Fall":			CheckBox Zoom_to_Foot value=0			CheckBox Zoom_to_Rise value=0			break	endswitch	If(Total_peaks_number==0)		abort	endif	Draw_lines_zoom_window(Peak_ID-1)EndFunction Change_Table (ctrlName,checked) : CheckBoxControl	String ctrlName	Variable checked	SetDataFolder $"root:Quanta"	NVAR Show_Time=Show_Time	NVAR Show_Base=Show_Base	NVAR Show_Width=Show_Width	NVAR Show_H=Show_H	NVAR Show_Q=Show_Q	NVAR Show_Molec=Show_Molec	NVAR Show_Rise_t=Show_Rise_t	NVAR Show_Rise_r=Show_Rise_r	NVAR Show_Fall_t=Show_Fall_t	NVAR Show_Fall_r=Show_Fall_r	NVAR Show_Ft_H=Show_Ft_H	NVAR Show_Ft_width=Show_Ft_width	NVAR Show_Ft_Q=Show_Ft_Q	NVAR Show_Ft_molec=Show_Ft_molec	NVAR Rise_Low_Prc=Rise_Low_Prc	NVAR Rise_Hi_Prc=Rise_Hi_Prc	SVAR Values_to_show=Values_to_show	Values_to_show="Peak_Num"	SVAR Stats_names=Stats_names	Stats_names="\tIntSp[ms]\tID#\t"	if(CheckName("Options_Tab_Panels", 9)!=0)		ControlInfo/W=Options_Tab_Panels Results_show1; Show_Time=V_Value	endif	If(Show_Time==1)		Values_to_show+=",T_Max"		Stats_names+="Max[s]\t"	endif	if(CheckName("Options_Tab_Panels", 9)!=0)		ControlInfo/W=Options_Tab_Panels Results_Show2; Show_Base=V_Value	endif	If(Show_Base==1)		Values_to_show+=",Peak_Base"		Stats_names+="Base[ms]\t"	endif	if(CheckName("Options_Tab_Panels", 9)!=0)		ControlInfo/W=Options_Tab_Panels Results_Show4; Show_Width=V_Value	endif	If(Show_Width==1)		Values_to_show+=",Peak_t05"		Stats_names+="t1/2[ms]\t"	endif	if(CheckName("Options_Tab_Panels", 9)!=0)		ControlInfo/W=Options_Tab_Panels Results_Show5; Show_H=V_Value	endif	If(Show_H==1)		Values_to_show+=",Peak_Imax"		Stats_names+="Imax [pA]\t"	endif	if(CheckName("Options_Tab_Panels", 9)!=0)		ControlInfo/W=Options_Tab_Panels Results_Show6; Show_Q=V_Value	endif	If(Show_Q==1)		Values_to_show+=",Peak_Q"		Stats_names+="Q [pC]\t"	endif	if(CheckName("Options_Tab_Panels", 9)!=0)		ControlInfo/W=Options_Tab_Panels Results_Show7; Show_Molec=V_Value	endif	If(Show_Molec==1)		Values_to_show+=",Peak_Molec"		Stats_names+="Molec\t"	endif	if(CheckName("Options_Tab_Panels", 9)!=0)		ControlInfo/W=Options_Tab_Panels Results_Show8; Show_Rise_t=V_Value	endif	If(Show_Rise_t==1)		Values_to_show+=",Rise_time"		Stats_names+="Rise("+num2str(Rise_Low_Prc)+"-"+num2str(Rise_Hi_Prc)+")[ms]\t"	endif	if(CheckName("Options_Tab_Panels", 9)!=0)		ControlInfo/W=Options_Tab_Panels Results_Show9; Show_Rise_r=V_Value	endif	If(Show_Rise_r==1)		Values_to_show+=",Rise_slope"		Stats_names+="Rise[pA/ms]\t"	endif	if(CheckName("Options_Tab_Panels", 9)!=0)		ControlInfo/W=Options_Tab_Panels Results_Show10; Show_Fall_t=V_Value	endif	If(Show_Fall_t==1)		Values_to_show+=",Fall_time"		Stats_names+="Fall(75-25) [ms]\t"	endif	if(CheckName("Options_Tab_Panels", 9)!=0)		ControlInfo/W=Options_Tab_Panels Results_Show11; Show_Fall_r=V_Value	endif	If(Show_Fall_r==1)		Values_to_show+=",Fall_fit"		Stats_names+="Fall Fit\t"		SVAR Fit_method=Fit_method		If (cmpstr(Fit_method,"Line")==0)			Values_to_show+=",Fall_slope"			Stats_names+="Fall slope[pA/ms]\t"		else			Values_to_show+=",Fall_slope,Fall_slope2"			Stats_names+="Fall tau1[ms]\tFall tau2[ms]\t"		endif	endif	if(CheckName("Options_Tab_Panels", 9)!=0)		ControlInfo/W=Options_Tab_Panels Results_Show12; Show_Ft_H=V_Value	endif	If(Show_Ft_H==1)		Values_to_show+=",Foot_I"		Stats_names+="I(foot) [pA]\t"	endif	if(CheckName("Options_Tab_Panels", 9)!=0)		ControlInfo/W=Options_Tab_Panels Results_Show13; Show_Ft_width=V_Value	endif	If(Show_Ft_width==1)		Values_to_show+=",Foot_W"		Stats_names+="T(foot) [ms]\t"	endif	if(CheckName("Options_Tab_Panels", 9)!=0)		ControlInfo/W=Options_Tab_Panels Results_Show14; Show_Ft_Q=V_Value	endif	If(Show_Ft_Q==1)		Values_to_show+=",Foot_Q"		Stats_names+="Q(foot) [pC]\t"	endif	if(CheckName("Options_Tab_Panels", 9)!=0)		ControlInfo/W=Options_Tab_Panels Results_Show15; Show_Ft_molec=V_Value	endif	If(Show_Ft_molec==1)		Values_to_show+=",Foot_Molec"		Stats_names+="Q(Foot) [Molec]"	endif	Stats_names+="\r\r"	Dowindow/K Peak_stats_Table1	Peak_stats_Table()	AutoPositionWindow/E/M=0/R=Main_window Peak_stats_Table1	Dowindow/F Options_Tab_Panels	NVAR Total_peaks_number=Total_peaks_number	NVAR Peak_ID=Peak_ID	NVAR Show_Legend=Show_Legend	If((Total_peaks_number)&(Show_Legend==1))		Generate_annotation(Peak_ID-1)	endifEndFunction Change_Table_RiseTime(ctrlName,varNum,varStr,varName) : SetVariableControl	String ctrlName	Variable varNum	String varStr	String varName	Change_Table ("q",1)EndFunction Peak_stats_Table()	SetDataFolder $"root:Quanta"	SVAR Values_to_show=Values_to_show	NVAR Scrn_hight_Points=Scrn_hight_Points	Variable Resolution=ScreenResolution	Variable Table_Top=Scrn_hight_Points-180*96/Resolution	Variable Table_Bottom=Scrn_hight_Points-75*96/Resolution	String Things_to_go="Edit/K=1/W=(2,"+num2str(Table_Top)+",50,"+num2str(Table_Bottom)+") "+ Values_to_show//	String Things_to_go="Edit/K=1/W=(2,415,50,415+"+num2str(Table_hight)+") "+ Values_to_show	execute Things_to_go	DoWindow/C Peak_stats_Table1	DoWindow/T Peak_stats_Table1,"Spikes statistics"	execute "Peak_stats_Table_formats()"	NVAR Table_L=Table_L	MoveWindow 2,Table_Top,Table_L,Table_Bottom	KillVariables /Z Table_LEndWindow Peak_stats_Table_formats()	SetDataFolder $"root:Quanta"	Variable/G Table_L=40	ModifyTable size(Point)=8,width(Point)=0,size(Peak_Num)=8,style(Peak_Num)=1,width(Peak_Num)=20, title(Peak_Num)="Id#",rgb(Peak_Num)=(65280,0,0),font(Peak_Num)="Arial"	if(strsearch(Values_to_show,"T_Max",0)!=-1)		ModifyTable size(T_Max)=7,font(T_Max)="Arial",style(T_Max)=1,format(T_Max)=3,digits(T_Max)=3,width(T_Max)=35,title(T_Max)="Max [s]",trailingZeros(T_Max)=1		Table_L+=35	endif	if(strsearch(Values_to_show,"Peak_Base",0)!=-1)		ModifyTable size(Peak_Base)=8,font(Peak_Base)="Arial",style(Peak_Base)=1,format(Peak_Base)=3,digits(Peak_Base)=3,width(Peak_Base)=40, title(Peak_Base)="Base [ms]",trailingZeros(Peak_Base)=1,rgb(Peak_Base)=(0,12800,52224)		Table_L+=40	endif	if(strsearch(Values_to_show,"Peak_t05",0)!=-1)		ModifyTable size(Peak_t05)=8,font(Peak_t05)="Arial",style(Peak_t05)=1,format(Peak_t05)=3,digits(Peak_t05)=3,width(Peak_t05)=40, title(Peak_t05)="t1/2 [ms]",trailingZeros(Peak_t05)=1,rgb(Peak_t05)=(0,12800,52224)		Table_L+=40	endif	if(strsearch(Values_to_show,"Peak_Imax",0)!=-1)		ModifyTable style(Peak_Imax)=1,font(Peak_Imax)="Arial",digits(Peak_Imax)=2,width(Peak_Imax)=40,title(Peak_Imax)="Imax [pA]",size(Peak_Imax)=8,rgb(Peak_Imax)=(0,12800,52224),trailingZeros(Peak_Imax)=1		Table_L+=40	endif	if(strsearch(Values_to_show,"Peak_Q",0)!=-1)		ModifyTable size(Peak_Q)=8,font(Peak_Q)="Arial",style(Peak_Q)=1,width(Peak_Q)=40,title(Peak_Q)="Q [pC]",rgb(Peak_Q)=(0,12800,52224),trailingZeros(Peak_Q)=1		Table_L+=40	endif	if(strsearch(Values_to_show,"Peak_Molec",0)!=-1)		ModifyTable size(Peak_Molec)=7,font(Peak_Molec)="Arial",style(Peak_Molec)=1,sigDigits(Peak_Molec)=4,width(Peak_Molec)=40,title(Peak_Molec)="Molecules",rgb(Peak_Molec)=(0,12800,52224),trailingZeros(Peak_Molec)=1		Table_L+=40	endif	if(strsearch(Values_to_show,"Rise_time",0)!=-1)		Variable/G Rise_Low_Prc,Rise_Hi_Prc		String Column_name="Rise("+num2str(Rise_Low_Prc)+"-"+num2str(Rise_Hi_Prc)+")[ms]\t"		ModifyTable size(Rise_time)=8,font(Rise_time)="Arial", style(Rise_time)=1,sigDigits(Rise_time)=3,width(Rise_time)=40,title(Rise_time)=Column_name,rgb(Rise_time)=(0,12800,52224),trailingZeros(Rise_time)=1		Table_L+=40	endif	if(strsearch(Values_to_show,"Rise_slope",0)!=-1)		ModifyTable size(Rise_slope)=8,font(Rise_slope)="Arial",style(Rise_slope)=1,sigDigits(Rise_slope)=3,width(Rise_slope)=40,title(Rise_slope)="Rise[pA/ms]",rgb(Rise_slope)=(0,12800,52224),trailingZeros(Rise_slope)=1		Table_L+=40	endif	if(strsearch(Values_to_show,"Fall_time",0)!=-1)		ModifyTable sigDigits(Fall_time)=3,font(Fall_time)="Arial",width(Fall_time)=40,title(Fall_time)="Fall(75-25)[ms]",size(Fall_time)=8,style(Fall_time)=1,rgb(Fall_time)=(0,12800,52224),trailingZeros(Fall_time)=1		Table_L+=40	endif	if(strsearch(Values_to_show,"Fall_fit",0)!=-1)		ModifyTable size(Fall_fit)=7,font(Fall_fit)="Arial",style(Fall_fit)=1,format(Fall_fit)=3,digits(Fall_fit)=3,width(Fall_fit)=35,title(Fall_fit)="Fall fit"		Table_L+=35	endif	if(strsearch(Values_to_show,"Fall_slope",0)!=-1)		String/G Fit_method		ModifyTable size(Fall_slope)=8,font(Fall_slope)="Arial",style(Fall_slope)=1,sigDigits(Fall_slope)=3,width(Fall_slope)=40,rgb(Fall_slope)=(0,12800,52224),trailingZeros(Fall_slope)=1		If (cmpstr(Fit_method,"Line")==0)			ModifyTable title(Fall_slope)="Slope[pA/ms]"		else			ModifyTable title(Fall_slope)="Tau1 [ms]"		endif		Table_L+=40	endif	if(strsearch(Values_to_show,"Fall_slope2",0)!=-1)		ModifyTable size(Fall_slope2)=8,font(Fall_slope2)="Arial",style(Fall_slope2)=1,sigDigits(Fall_slope2)=3,width(Fall_slope2)=40,title(Fall_slope2)="Tau2 [ms]",rgb(Fall_slope2)=(0,12800,52224),trailingZeros(Fall_slope2)=1		Table_L+=40	endif	if(strsearch(Values_to_show,"Foot_I",0)!=-1)		ModifyTable width(Foot_I)=40,font(Foot_I)="Arial",title(Foot_I)="I(foot) [pA]",rgb(Foot_I)=(26112,0,0),size(Foot_I)=8,style(Foot_I)=1,digits(Foot_I)=2,trailingZeros(Foot_I)=1		Table_L+=40	endif	if(strsearch(Values_to_show,"Foot_W",0)!=-1)		ModifyTable width(Foot_W)=40,font(Foot_W)="Arial",title(Foot_W)="T(foot) [ms]",rgb(Foot_W)=(26112,0,0),size(Foot_W)=8,style(Foot_W)=1,digits(Foot_W)=2,trailingZeros(Foot_W)=1		Table_L+=40	endif	if(strsearch(Values_to_show,"Foot_Q",0)!=-1)		ModifyTable size(Foot_Q)=8,font(Foot_Q)="Arial",style(Foot_Q)=1,width(Foot_Q)=40,title(Foot_Q)="Q(foot) [pC]",rgb(Foot_Q)=(26112,0,0),trailingZeros(Foot_Q)=1		Table_L+=40	endif	if(strsearch(Values_to_show,"Foot_Molec",0)!=-1)		ModifyTable size(Foot_Molec)=8,font(Foot_Molec)="Arial",style(Foot_Molec)=1,sigDigits(Foot_Molec)=4,width(Foot_Molec)=40,title(Foot_Molec)="Q(Foot) [Molec]",rgb(Foot_Molec)=(26112,0,0),trailingZeros(Foot_Molec)=1		Table_L+=40	endif//	MoveWindow 2,415,Table_L,510EndMacroFunction Recalculate_All_Peaks(Do_what)	String Do_what	SetDataFolder $"root:Quanta"	Wave Working_trace_copy=Working_trace_copy	Wave T_Max=T_Max	Wave T_Bkg1=T_Bkg1	Wave T_Bkg2=T_Bkg2	Wave Foot_end=Foot_end	NVAR Total_peaks_number=Total_peaks_number	NVAR Peak_ID=Peak_ID	NVAR Bkg_noise_Start=Bkg_noise_Start	NVAR Bkg_noise_End=Bkg_noise_End	Variable/G Bkg_noise_Start_p	Variable/G Bkg_noise_End_p	If(cmpstr(Do_what,"Save_old")==0)		Duplicate/O T_Max T_Max_pnt		Duplicate/O T_Bkg1 T_Bkg1_pnt		Duplicate/O T_Bkg2 T_Bkg2_pnt		T_Max_pnt[0,]=x2pnt(Working_trace_copy,T_Max[p])		T_Bkg1_pnt[0,]=x2pnt(Working_trace_copy,T_Bkg1[p])		T_Bkg2_pnt[0,]=x2pnt(Working_trace_copy,T_Bkg2[p])		Bkg_noise_Start_p=x2pnt(Working_trace_copy,Bkg_noise_Start)		Bkg_noise_End_p=x2pnt(Working_trace_copy,Bkg_noise_End)	endif	If(cmpstr(Do_what,"Recalc_new")==0)		Variable i, Max_X, Start_X, End_X		Bkg_noise_Start=pnt2x(Working_trace_copy,Bkg_noise_Start_p)		Bkg_noise_End=pnt2x(Working_trace_copy,Bkg_noise_End_p)		Bkg_noise_Calc()		T_Max[0,]=pnt2x(Working_trace_copy,T_Max_pnt[p])		T_Bkg1[0,]=pnt2x(Working_trace_copy,T_Bkg1_pnt[p])		T_Bkg2[0,]=pnt2x(Working_trace_copy,T_Bkg2_pnt[p])		for(i=0;i<Total_peaks_number;i+=1)			Calc_Peak_Parameters(i,T_Bkg1[i],T_Bkg2[i])		endfor		KillVariables /Z Bkg_noise_Start_p,Bkg_noise_End_p		Killwaves/Z T_Max_pnt,T_Bkg1_pnt,T_Bkg2_pnt		Peak_ID=1		Draw_lines_All(0)	endifEndFunction Recalculate_Peaks_Btn(ctrlName) : ButtonControl	String ctrlName	SetDataFolder $"root:Quanta"	Wave Rise_Midpoint=Rise_Midpoint	Wave Rise_time=Rise_time	Wave/T Fall_fit=Fall_fit	NVAR Total_peaks_number=Total_peaks_number	Fall_fit=""	Rise_Midpoint=0	Rise_time=0	If (Total_peaks_number)		Recalculate_All_Peaks("Save_old")		Recalculate_All_Peaks("Recalc_new")	endif	Dowindow/F Options_Tab_PanelsEndFunction Adjust_Trace_Scales(ctrlName,varNum,varStr,varName) : SetVariableControl	String ctrlName	Variable varNum	String varStr	String varName	WAVE Working_trace_copy=Working_trace_copy	NVAR T_Start=T_Start	NVAR T_End=T_End	NVAR T_Delta=T_Delta	NVAR Gain=Gain	NVAR Gain_Temp=Gain_Temp	Variable N_pnts=numpnts(Working_trace_copy)	strswitch(ctrlName)		case "Scales_Start_time":				ControlInfo Scales_Change_End				If(V_Value)					T_Delta=((T_End-T_Start)/N_pnts)*1000				else					T_End=T_Start+T_delta/1000*N_pnts				endif			break		case "Scales_End_time":			T_Delta=((T_End-T_Start)/N_pnts)*1000			break		case "Scales_Delta_time":			T_End=T_Start+T_delta/1000*N_pnts			break	endswitch	Button Recalculate_btn fColor=(0,0,0),win=Options_Tab_Panels	Variable Old_T_Delta=(pnt2x(Working_trace_copy,1)-pnt2x(Working_trace_copy,0))*1000	If(Old_T_Delta!=T_Delta)		Button Recalculate_btn fColor=(0,39168,0),win=Options_Tab_Panels	endif	If((T_Start!=pnt2x(Working_trace_copy,0))%|(T_End!=pnt2x(Working_trace_copy,(numpnts(Working_trace_copy)-1))))		Button Recalculate_btn fColor=(0,39168,0),win=Options_Tab_Panels	endif	If(Gain_Temp!=Gain)		Button Recalculate_btn fColor=(0,39168,0),win=Options_Tab_Panels	endifEndFunction Change_trace_scales(ctrlName) : ButtonControl	String ctrlName	SetDataFolder $"root:Quanta"	WAVE Working_trace_copy=Working_trace_copy	NVAR Total_peaks_number=Total_peaks_number	NVAR Gain=Gain	NVAR Gain_Temp=Gain_Temp	NVAR Bkg_noise_I=Bkg_noise_I	NVAR Bkg_noise_dI=Bkg_noise_dI	NVAR Bkg_noise_Start=Bkg_noise_Start	NVAR Bkg_noise_End=Bkg_noise_End	NVAR T_Start=T_Start	NVAR T_End=T_End	NVAR T_Delta=T_Delta	Variable Changed=0	If(Gain_Temp!=Gain)		if(Total_peaks_number)			String Massage="Do You want to recalculate the parameters of the existing spikes? \r Press 'Yes' to Recalculate.\r Press 'No' to Delete the spikes.\rNote: Will not work on Separated and Split spikes!"			DoAlert 2, Massage			switch(V_Flag)				case 1:					Recalculate_All_Peaks("Save_old")					Change_trace_Y_scale()					Recalculate_All_Peaks("Recalc_new")					break				case 2:					De_novo()					GroupBox Bkg_HiLt, win=Main_window,disable=0					Bkg_noise_I=0					Bkg_noise_dI=0					Bkg_noise_Start=0					Bkg_noise_End=0					Change_trace_Y_scale()					break				case 3:					abort					break			endswitch		else			Change_trace_Y_scale()		endif	endif	Variable Old_T_Delta=(pnt2x(Working_trace_copy,1)-pnt2x(Working_trace_copy,0))*1000	If(Old_T_Delta!=T_Delta)		Change_trace_X_scale()	endif	If((T_Start!=pnt2x(Working_trace_copy,0))%|(T_End!=pnt2x(Working_trace_copy,(numpnts(Working_trace_copy)-1))))		Change_trace_X_scale()	endif	Button Recalculate_btn fColor=(0,0,0),win=Options_Tab_PanelsEndFunction Change_trace_X_scale()	SetDataFolder $"root:Quanta"	NVAR T_Start=T_Start	NVAR T_End=T_End	NVAR Peak_ID=Peak_ID	NVAR Bkg_noise_I=Bkg_noise_I	NVAR Bkg_noise_dI=Bkg_noise_dI	NVAR Bkg_noise_Start=Bkg_noise_Start	NVAR Bkg_noise_End=Bkg_noise_End	NVAR Total_peaks_number=Total_peaks_number	WAVE Working_trace_copy=Working_trace_copy	WAVE Orig_trace_copy=Orig_trace_copy	NVAR T_Delta=T_Delta	NVAR Gain=Gain	NVAR Gain_Temp=Gain_Temp	Variable N_pnts=numpnts(Working_trace_copy)	if(Total_peaks_number)		String Massage="All spikes will be Deleted! \r Press 'Yes' to proceed or 'No' to to Cancel"		DoAlert 1, Massage		If(V_Flag==1)			De_novo()		else			abort		endif	endif		Bkg_noise_I=0	Bkg_noise_dI=0	Bkg_noise_Start=0	Bkg_noise_End=0	GroupBox Bkg_HiLt, win=Main_window,disable=0	Dowindow/F Main_window	GetAxis /Q bottom	Variable P_min=x2pnt(Working_trace_copy,V_min)	Variable P_max=x2pnt(Working_trace_copy,V_max)	ControlInfo/W=Options_Tab_Panels Scales_Change_End	If(V_Value)		SetScale/I x T_Start,T_End,"s", Working_trace_copy		SetScale/I x T_Start,T_End,"s", Orig_trace_copy		T_Delta=((T_End-T_Start)/N_pnts)*1000	else		SetScale/P x T_Start,T_Delta/1000,"s", Working_trace_copy		SetScale/P x T_Start,T_Delta/1000,"s", Orig_trace_copy		T_End=T_Start+T_delta/1000*N_pnts	endif	Filter_Limits()	SetAxis bottom pnt2x(Working_trace_copy,P_min),pnt2x(Working_trace_copy,P_max)	Dowindow/F Options_Tab_PanelsEndFunction Change_trace_Y_scale()	SetDataFolder $"root:Quanta"	NVAR Total_peaks_number=Total_peaks_number	NVAR Gain=Gain	NVAR Gain_Temp=Gain_Temp	Wave Working_trace_copy=Working_trace_copy	Wave Orig_trace_copy=Orig_trace_copy		Working_trace_copy*=Gain_Temp/Gain	Orig_trace_copy*=Gain_Temp/Gain	SetAxis/A left	Gain=Gain_Temp	Dowindow/F Options_Tab_PanelsEndFunction Show_original_rec(ctrlName) : ButtonControl	String ctrlName	SetDataFolder $"root:Quanta"	If(exists("Orig_trace_copy")!=1)		abort	endif	If(cmpstr(ctrlName, "Show_orig")==0)		AppendToGraph Orig_trace_copy		ModifyGraph rgb(Orig_trace_copy)=(52224,52224,52224)		ReorderTraces Working_trace_copy,{Orig_trace_copy}		Button Show_orig rename=Hide_orig, title="Hide Orig"	else		RemoveFromGraph/Z Orig_trace_copy		Button Hide_orig rename=Show_orig, title="Show Orig"	endifEnd//________Digital Filters___________Function SmoothBtn_Q(ctrlname) : ButtonControl	String ctrlname	SetDataFolder $"root:Quanta"	If(exists("Orig_trace_copy")!=1)		abort	endif	Wave diff1=diff1	NVAR Smoothing_Factor=Smoothing_Factor	NVAR Overall_Filter=Overall_Filter	String Traces_Names=TraceNameList("","",1)	String One_trace	String TheTrace_Name=StringFromList(0,Traces_Names)	Variable q=0	Do		One_trace=Stringfromlist(q,Traces_Names)		If(strlen(One_trace)==0)			break		endif		If(stringmatch(One_trace, "Working_Trace_copy")==1)			TheTrace_Name=One_trace			break		endif		q+=1	while(1)	ControlInfo Smoth_meth	if(V_value==1)		//	Low-Pass Gaussian filter		Gaussian_Filter(TheTrace_Name,"Low")		If(Overall_Filter)			Overall_Filter=SQRT(((Overall_Filter*Smoothing_Factor)^2)/(Overall_Filter^2+Smoothing_Factor^2))		else			Overall_Filter=Smoothing_Factor		endif	endif	if(V_value==2)		//	High-Pass Gaussian filter		Gaussian_Filter(TheTrace_Name,"High")	endif	if(V_value==3)		//	Binomial (Gaussian) smoothing		Variable Binomial_coeff=Gaussian_to_Binomial_Calc(Smoothing_Factor)		Variable Real_cutoff=Binomial_to_Gaussian_Calc(Binomial_coeff)		Print "Trace was filtered with "+num2str(Real_cutoff)+"Hz (Binomial "+num2str(Binomial_coeff)+") -3dB Gaussian filter."		Smooth Binomial_coeff, $TheTrace_Name		If(Overall_Filter)			Overall_Filter=SQRT(((Overall_Filter*Smoothing_Factor)^2)/(Overall_Filter^2+Smoothing_Factor^2))		else			Overall_Filter=Smoothing_Factor		endif	endif	if(V_value==4)		//	Boxcar (sliding average) smoothing		Smooth/B Smoothing_Factor,$TheTrace_Name	endif	if(V_value==5)		//	Savitzky-Golay (polynomial) smoothing		Smooth/S=2 Smoothing_Factor, $TheTrace_Name	endif	//	Wave Working_Trace_copy=Working_Trace_copy	//	Duplicate/O Orig_trace_copy PostFilter_Residuals	//	PostFilter_Residuals-=Working_Trace_copy		SetDrawLayer /K UserFrontEndFunction Smooth_method_Q(ctrlName,popNum,popStr) : PopupMenuControl	String ctrlName	Variable popNum	String popStr	SetDataFolder $"root:Quanta"	NVAR Smoothing_Factor=Smoothing_Factor	NVAR T_Delta=T_Delta	Filter_Limits()	if(popNum==1)		Smoothing_Factor=500	endif	if(popNum==2)		Smoothing_Factor=0.1	endif	if(popNum==3)		Variable Min_Freq=Binomial_to_Gaussian_Calc(32767)		Smoothing_Factor=max(500,Min_Freq+1)	endif	if(popNum==4)		Smoothing_Factor=25	endif	if(popNum==5)		Smoothing_Factor=5	endifEndFunction Filter_Limits()	SetDataFolder $"root:Quanta"	NVAR T_Delta=T_Delta	ControlInfo/W=Main_window Smoth_meth	if(V_value==1)		SetVariable Smooth_F,win=Main_window, title="Hz",limits={1,(1/(T_Delta/1000))/2,10}		SetVariable Smooth_F,win=Main_window, help={"Low-pass cutoff frequency (-3dB) for Gaussian filter."}	endif	if(V_value==2)		SetVariable Smooth_F,win=Main_window, title="Hz",limits={0.0000001,(1/(T_Delta/1000))/2,0.01}		SetVariable Smooth_F,win=Main_window, help={"High-pass cutoff frequency (-3dB) for Gaussian filter."}	endif	if(V_value==3)		Variable Min_Freq=Binomial_to_Gaussian_Calc(32767)		SetVariable Smooth_F,win=Main_window, title="Hz",limits={Min_Freq+1,(1/(T_Delta/1000))/2,50}		SetVariable Smooth_F,win=Main_window, help={"Low-pass cutoff frequency (-3dB) for Gaussian filter."}	endif	if(V_value==4)		SetVariable Smooth_F,win=Main_window, title="Points",limits={1,32767,10}		SetVariable Smooth_F,win=Main_window, help={"The number of points in the smoothing window for Boxcar smoothing."}	endif	if(V_value==5)		SetVariable Smooth_F,win=Main_window, title="Points",limits={5,25,2}		SetVariable Smooth_F,win=Main_window, help={"The number of points in the smoothing window for Savitzky-Golay smoothing."}	endifEndFunction Additional_Filters(ctrlName,checked) : CheckBoxControl	String ctrlName	Variable checked	SetDataFolder $"root:Quanta"	NVAR Smooth_more=Smooth_more	NVAR Smooth_Derivative=Smooth_Derivative	strswitch(ctrlName)		case "Scales_Smooth_Add":				If(checked==1)					Smooth_more=1				else					Smooth_more=0				endif			break		case "Scales_Smooth_Diff":				If(checked==1)					Smooth_Derivative=1				else					Smooth_Derivative=0				endif			break	endswitchEndFunction Gaussian_Filter(Filtered_Trace_Name,Type)	String Filtered_Trace_Name	String Type	SetDataFolder $"root:Quanta"	Wave FFT_wave=$Filtered_Trace_Name	Variable npnts= numpnts(FFT_wave)	Variable Start_X=pnt2x(FFT_wave, 0)	Variable End_X=pnt2x(FFT_wave,npnts)	If(npnts>1e+6)		DoAlert 1, "During Fourier transforms of large traces (such as this one) the computer may freeze for a few seconds. As an alternative use Binomial smoothing.\rTo continue with Gaussian filter press 'Yes' or press 'No' to cancel."		If(V_Flag!=1)			return 0		endif	endif	NVAR Smoothing_Factor=Smoothing_Factor	Variable Cutoff_Amplitude=1/(sqrt(2))		// 50% power	Variable Gauss_Width= Smoothing_Factor/sqrt(-ln(Cutoff_Amplitude))	Redimension/N=(npnts*2) FFT_wave		// the wave has to have an EVEN number of rows	FFT FFT_wave	WAVE/C FFT_wave_complex=FFT_wave	If(cmpstr(Type,"Low")==0)		FFT_wave_complex*=cmplx(exp(-(x^2/(Gauss_Width^2))),0)	//	Low-pass Gaussian	else		FFT_wave_complex*=cmplx(1-exp(-(x^2/(Gauss_Width^2))),0)	//	High-pass Gaussian	endif	IFFT FFT_wave	Redimension/N=(npnts) FFT_wave	SetScale/I x Start_X,End_X,"s", FFT_waveEndFunction Gaussian_to_Binomial_Calc(Frequency_Hz)	Variable Frequency_Hz	SetDataFolder $"root:Quanta"	NVAR T_Delta=T_Delta	Variable Binomial_coeff=0.02873*(Frequency_Hz * T_Delta/1000) ^(-2.0764)	Return max(round(Binomial_coeff),1)endFunction Binomial_to_Gaussian_Calc(Binomial_coeff)	Variable Binomial_coeff	SetDataFolder $"root:Quanta"	NVAR T_Delta=T_Delta	Variable Frequency_Hz=0.1809/(T_Delta/1000)*Binomial_coeff ^(-0.4815)	Return trunc(Frequency_Hz)endFunction Filter_Estimate(ctrlName) : ButtonControl	String ctrlName	SetDataFolder $"root:Quanta"	Wave Working_trace_copy=Working_trace_copy	Variable Point1,Point2,Freq	If(numpnts(Working_trace_copy)<=1)		print "No data loaded!"		abort	endif	Duplicate/O Working_trace_copy diff1_nosmooth	Differentiate diff1_nosmooth	WaveStats/Q diff1_nosmooth		Variable dIMax=V_max		FindLevel/Q /R=(V_maxloc,0) diff1_nosmooth,0	Point1=V_LevelX	FindLevel/Q /R=(V_maxloc,) diff1_nosmooth,0	Point2=V_LevelX		Variable Imax=Working_trace_copy(Point2)-Working_trace_copy(Point1)	Freq=dIMax/Imax/2		Duplicate/O Working_trace_copy diff1_nosmooth	Variable Binomial_coeff=Gaussian_to_Binomial_Calc(Freq*3)	Smooth Binomial_coeff, diff1_nosmooth	Differentiate diff1_nosmooth	WaveStats/Q diff1_nosmooth	dIMax=V_max	FindLevel/Q /R=(V_maxloc,0) diff1_nosmooth,0	Point1=V_LevelX	FindLevel/Q /R=(V_maxloc,) diff1_nosmooth,0	Point2=V_LevelX	Freq=dIMax/Imax/2	MoveWindow/C 1,1,1,1	Print "\r"	String Formated_String	sprintf Formated_String, "%.0W1PHz", Freq	Print "Signal frequency is ~",Formated_String	Print "Recommended filters:"	sprintf Formated_String, "%.0W1PHz", 2*Freq	Print "\tSignal smoothing: Binomial",Formated_String	sprintf Formated_String, "%.0W1PHz", Freq/2	Print "\tAdditional smoothing: Binomial",Formated_String	sprintf Formated_String, "%.0W1PHz", Freq	Print "\t1st derrivative smoothing: Binomial",Formated_Stringend//	Fall_fit_Extrap[i]=//	StringByKey("N", Fall_fit_Extrap[i]) 		Spike number in a series of separated spikes//	StringByKey("Total", Fall_fit_Extrap[i]) 	Total number of separated spikes//	StringByKey("Fit", Fall_fit_Extrap[i])	Function used to extrapolate the falling phase of the spike (Exp or Line)//	StringByKey("Tau", Fall_fit_Extrap[i])	Time constant of exponential decay (user-controlled). Equals -1 if linear fit is used.//	Also://	ReplaceStringByKey("Fit", Fall_fit_Extrap[i], "Exp")//	HAPPY RECORDINGS!
