# Supplementary material for: Cell-type-specific regulation of neuronal intrinsic excitability by macroautophagy
Source: eLife. 2020 Jan 8;9:e50843. doi: 10.7554/eLife.50843 (PMC6984822; doi:10.7554/eLife.50843)
Supplement: Supplementary file 1. [file elife-50843-supp1.docx]

Cell-type specific regulation of neuronal intrinsic excitability by macroautophagy.

**Authors:** O. Lieberman, M. Frier, A McGuirt, C Griffey, E Rafikian, M Yang, A Yamamoto, A Borgkvist, E Santini, D Sulzer.

**Supplemental Material**

Tables S1-S3

Supplemental References

***Table S1***

| Primer | Sequence | Notes |
| --- | --- | --- |
| MF3 | AAAAAACTCGAGATGGGCAGTGTGAGAACCAAC | 5' Kir cloning XhoI |
| MF4 | AAAAAAGGATCCGTTATCTCCGATTCTCGCCTTA | 3' Kir cloning BamHI |
| MF12 fwd | ccagtgctctttgaagagaGacactactataaagta | K334R fwd SDM |
| MF12 rev | tactttatagtagtgtCtctcttcaaagagcactgg | K334R rev SDM |
| MF13 Fwd | gagaaacactactataGagtagactattcaagattc | K338R fwd SDM |
| MF13 Rev | gaatcttgaatagtctactCtatagtagtgtttctc | K338R rev SDM |
| MF14 Fwd | agactattcaagattccataGgacttatgaagtacctaa | K346R fwd SDM |
| MF14 Rev | ttaggtacttcataagtcCtatggaatcttgaatagtct | K346R rev SDM |
| MF23 | AAAAAAAAGCTTatgggcagtgtgagaaccaac | 5' Kir2.1 --> pcDNA HINDIII |
| MF41 Rev | tactttatagtagtgttGctcttcaaagagcactgg | K334Q rev SDM |
| MF41 Fwd | ccagtgctctttgaagagCaacactactataaagta | K334Q fwd SDM |

***Table S2***

| Plasmid | Notes | Source |
| --- | --- | --- |
| FUGW-VAMP2-FLAG-SNAP |  | (Sheehan et al., 2016) |
| pAAV-mmKir2.1 |  | Gift of C. Kellendonk |
| FUGW-mmKir2.1-FLAG-SNAP | PCR-subcloning of mmKir2.1 from pAAV-mmKir2.1 to FUGW-VAMP2-FLAG-SNAP using XhoI BamHI sites | This study |
| FUGW-mmKir2.1-ExtHA-FLAG-SNAP | HA tag (YPYDVPDYA) inserted at amino acid 114 of Kir2.1 | This study; genewiz custom synthesis |
| FUGW-mmKir2.1-K334R-ExtHA-FLAG-SNAP | Site-directed mutagenesis of mmKir2.1 🡪 K334R | This study |
| FUGW-mmKir2.1-K338R-ExtHA-FLAG-SNAP | Site-directed mutagenesis of mmKir2.1 🡪 K338R | This study |
| FUGW-mmKir2.1-K346R-ExtHA-FLAG-SNAP | Site-directed mutagenesis of mmKir2.1 🡪 K346R | This study |
| FUGW-mmKir2.1-K334R-FLAG-SNAP | Site-directed mutagenesis of mmKir2.1 🡪 K334R | This study |
| FUGW-mmKir2.1-K338R-FLAG-SNAP | Site-directed mutagenesis of mmKir2.1 🡪 K338R | This study |
| FUGW-mmKir2.1-K346R-FLAG-SNAP | Site-directed mutagenesis of mmKir2.1 🡪 K346R | This study |
| pcDNA3-EGFP |  | Addgene 13031 |
| pcDNA3-mmKir2.1-EGFP | PCR-subcloning of mmKir2.1 from **FUGW-mmKir2.1-FLAG-SNAP** to pcDNA3-EGFP using HindIII BamHI sites | This study |
| pcDNA3-mmKir2.1-ExtHA-EGFP | PCR-subcloning of mmKir2.1 from **FUGW-mmKir2.1-ExtHA-FLAG-SNAP** to pcDNA3-EGFP using HindIII BamHI sites | This study |
| pcDNA3-mmKir2.1-K334R-EGFP | PCR-subcloning of mmKir2.1-K334R from **FUGW-mmKir2.1-K334R-FLAG-SNAP** to pcDNA3-EGFP using HindIII BamHI sites | This study |
| pcDNA3-mmKir2.1-K338R-EGFP | PCR-subcloning of mmKir2.1-K338R from **FUGW-mmKir2.1-K338R-FLAG-SNAP** to pcDNA3-EGFP using HindIII BamHI sites | This study |
| pcDNA3-mmKir2.1-K346R-EGFP | PCR-subcloning of mmKir2.1-K346R from **FUGW-mmKir2.1-K346R-FLAG-SNAP** to pcDNA3-EGFP using HindIII BamHI sites | This study |
| pcDNA3-mmKir2.1-K334R-ExtHA-EGFP | PCR-subcloning of mmKir2.1-K334R from **FUGW-mmKir2.1-K334R-ExtHA-FLAG-SNAP** to pcDNA3-EGFP using HindIII BamHI sites | This study |
| pEGFP-LC3 |  | (Kabeya et al., 2000) |
| Rab7GFP/Rab5GFP |  |  |
|  |  |  |

***Table S3***

| Antibody | Source | Catalog Number | IF concentration/notes | WB concentration/notes |
| --- | --- | --- | --- | --- |
| Rabbit anti-Red fluorescent protein polyclonal | Rockland | Cat # 600-401-379 | 1:500 | N/A |
| Rabbit anti-DARPP32 monoclonal | Cell Signaling Technology | Cat # 2306S | 1:500 | 1:2000-5000 |
| Mouse anti-beta actin monoclonal | Novus Biologicals | Cat # NB600-501 | N/A | 1:5000 |
| Mouse anti-Kir2.1 monoclonal | Antibodies Incorporated | Item # 73-210  RRID:AB_11000720 | N/A | 1:1000 |
| Mouse anti-Kir2.3 monoclonal | Antibodies Incorporated | Item # 75-069  RRID:AB_2130742 | N/A | 1:1000 |
| Mouse anti-K_v_1.2_­­_ monoclonal | Antibodies Incorporated | Item # 75-008  RRID:AB_2296313 | N/A | 1:1000 |
| Rabbit anti-PSD95 polyclonal | Abcam | Cat # Ab18258 | N/A | 1:1000 |
| Guinea pig anti p62 polyclonal | American Research Products | Cat # 03-GP62-C | 1:100-1:200 | N/A |
| Rabbit anti p62 polyclonal | MBL | Cat # PM045 | N/A | 1:1000 |
| Rabbit anti LC3B polyclonal | Novus Biologicals | Cat # NB600-1384 | N/A | 1:1000 |
| Chicken anti GFP polyclonal | Abcam | Cat # Ab13970 | 1:1000 | N/A |
| Rabbit anti EEA1 polyclonal | Cell Signaling Technology | Cat # 2411 | 1:100 | N/A |
| Rat anti Lamp1 monoclonal | Iowa Hybridoma Bank | Cat # 1D4B | 1:100 | N/A |
| Rabbit anti Kir2.1 polyclonal | Alomone Labs | Cat # APC-026 | N/A | 1:1000 |
| Mouse anti ubiquitin antibody monoclonal VU-1 | LifeSensors | Cat # VU101 | N/A | 1:1000 |
| Mouse anti-acetyl-lysine monoclonal, clone 4G12 | Millipore | Cat # 05-515 | N/A | 1:1000 |
| Mouse Anti-Flag M2 monoclonal | Sigma | Cat # F1804 | 1:100-200 | 1:1000 |
| Rabbit anti-HA polyclonal | Abcam | Cat # ab9110 | 1:100-200 | 1:1000 |
| Rabbit anti Ctip2 monoclonal | Abcam | Cat # Ab18465 | 1:1000 | N/A |
| Rabbit anti Met-enk polyclonal | Immunostar | Cat # 20065 | 1:500 | N/A |
| Mouse anti-NeuN, monoclonal clone A60 | Millipore | Cat # MAB377 | 1:1000 | N/A |
| Goat anti Guinea Pig IgG (H+L) Secondary antibody, Alexa 488 | Invitrogen | Cat # A-11073 | 1:500 | N/A |
| Goat anti Guinea Pig IgG (H+L) Secondary antibody, Alexa 647 | Invitrogen | Cat # A-24150 | 1:500 | N/A |
| Donkey anti-Rabbit IgG (H+L) Secondary Antibody, Alexa 488 | Invitrogen | Cat # A-21206 | 1:500 | N/A |
| Donkey anti-Rabbit IgG (H+L) Secondary Antibody, Alexa 594 | Invitrogen | Cat # A-21207 | 1:500 | N/A |
| Goat anti-Mouse IgG1 (H+L) Secondary Antibody, Alexa 488 | Invitrogen | Cat # A-21121 |  |  |
| Donkey anti-Mouse IgG1 (H+L) Secondary Antibody, Alexa 594 | Invitrogen | Cat # A-21125 | 1:500 | N/A |
| Goat anti-Chicken IgY (H+L) Secondary Antibody, Alexa 488 | Invitrogen | Cat # A-11039 | 1:500 | N/A |
| Goat anti-Rat IgG1 (H+L) Secondary Antibody, Alexa 647 | Invitrogen | Cat # A-21248 | 1:1000 | N/A |
| Donkey anti-Rabbit IgG (H+L) Secondary IRDye 680LT | LI-COR | P/N 925-68023 | N/A | 1:5000 |
| Streptavidin, Alexa 488 conjugate | Invitrogen | S11223 | 1:200 | N/A |
| Donkey anti-Mouse IgG (H+L) conjugated to HRP | Jackson Immunoresearch | Code: 715-035-151 | N/A | 1:5000 |
| Donkey anti-Rabbit IgG (H+L) conjugated to HRP | Jackson Immunoresearch | Code: 715-035-152 | N/A | 1:5000 |

Bibliography

Kabeya, Y., Mizushima, N., Ueno, T., et al. 2000. LC3, a mammalian homologue of yeast Apg8p, is localized in autophagosome membranes after processing. *The EMBO Journal* 19(21), pp. 5720–5728.

Sheehan, P., Zhu, M., Beskow, A., Vollmer, C. and Waites, C.L. 2016. Activity-Dependent Degradation of Synaptic Vesicle Proteins Requires Rab35 and the ESCRT Pathway. *The Journal of Neuroscience* 36(33), pp. 8668–8686.
